# Supplementary figures and images for: Dating genomic variants and shared ancestry in population-scale sequencing data
Source: PLoS Biol. 2020 Jan 17;18(1):e3000586. doi: 10.1371/journal.pbio.3000586 (PMC6992231; doi:10.1371/journal.pbio.3000586)

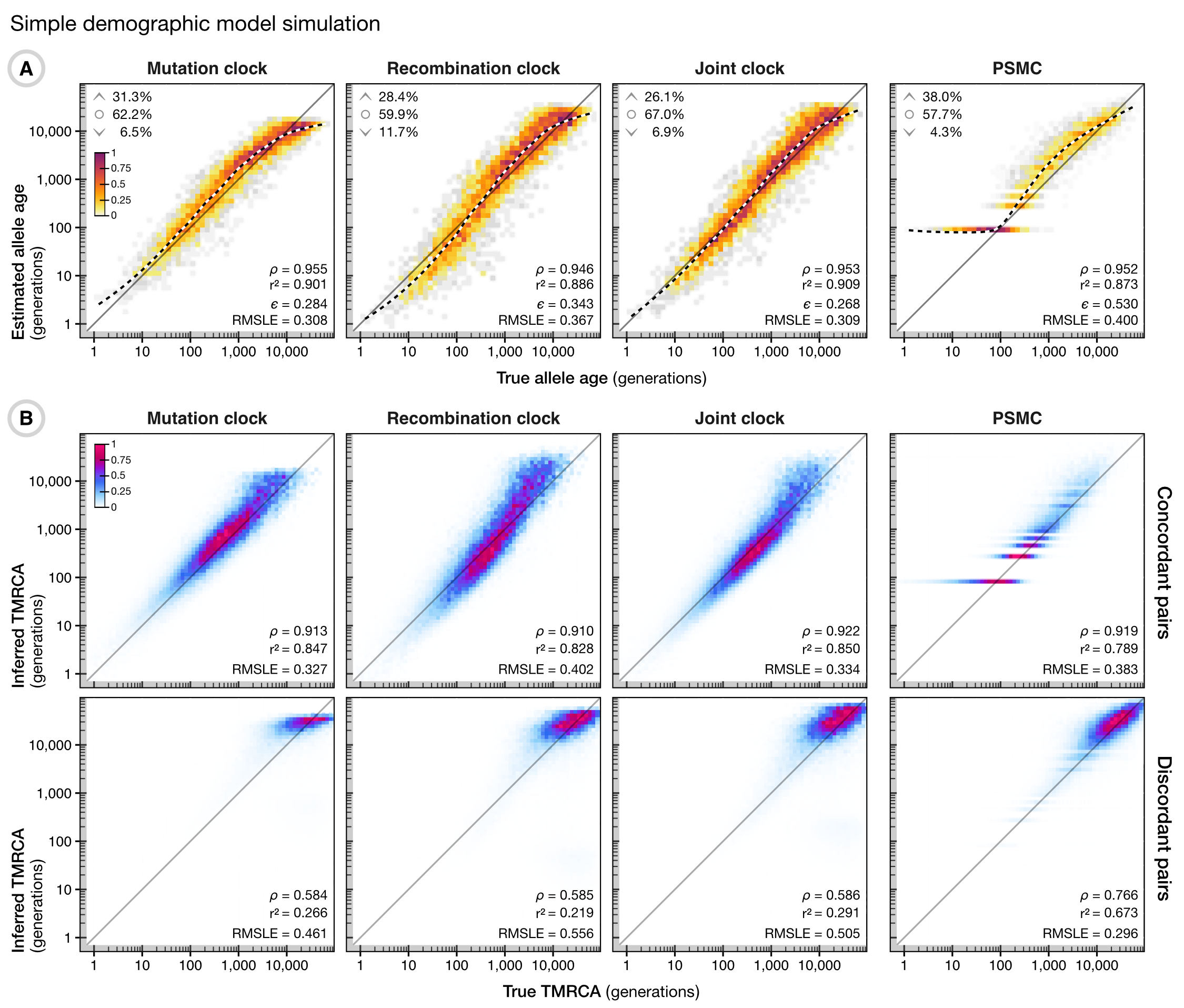

Supplement: S1 Fig — Allele age estimation and TMRCA inference in a simulation of a 100-Mb region with sample size N = 1,000, effective population size Ne = 10,000, constant mutation rate (μ = 1 × 10^−8^ per site per generation), and constant recombination rate (r = 1 × 10^−8^ per site per generation). (A) Relationship between the true allele age (geometric mean of lower and upper age of the branch on which a mutation occurred; x axis) and estimated allele age (y axis), estimated using the mutation clock, recombination clock, and joint clock models (left) and PSMC (right) for the same set of 5,000 variants, randomly sampled at allele count 1 < x < N. Colors indicate the density scaled by the maximum per panel. Upper inserts indicate the fraction of sites where the point estimate (mode of the composite posterior distribution) of allele age lies above the upper age of the branch on which the mutation occurred (^), below the lower age (˅), or within the range of the branch (∘). Lower inserts indicate the Spearman rank correlation statistic ρ, the square of the Pearson correlation coefficient (on log scale) r2, the interval-adjusted error metric ε, and the RMSLE. Also shown is an LOESS fit (second-degree polynomials, neighborhood proportion α = 0.25; dashed line). (B) Relationship between true TMRCA for a haplotype pair at a given site and corresponding inferred TMRCA (mean of posterior distribution), shown separately for concordant and discordant pairs. The same sets of haplotype pairs were analyzed under each clock model in GEVA (left) and PSMC (right). Colors indicate the density scaled by the maximum per panel. Lower inserts indicate the Spearman rank correlation statistic ρ, the square of the Pearson correlation coefficient (on log scale) r2, and the RMSLE. GEVA, Genealogical Estimation of Variant Age; LOESS, locally estimated scatterplot smoothing; PSMC, pairwise sequentially Markovian coalescent; RMSLE, root mean-square log10 error; TMRCA, time to the most recent common ancestor. (TIF) [file pbio.3000586.s001.tif]

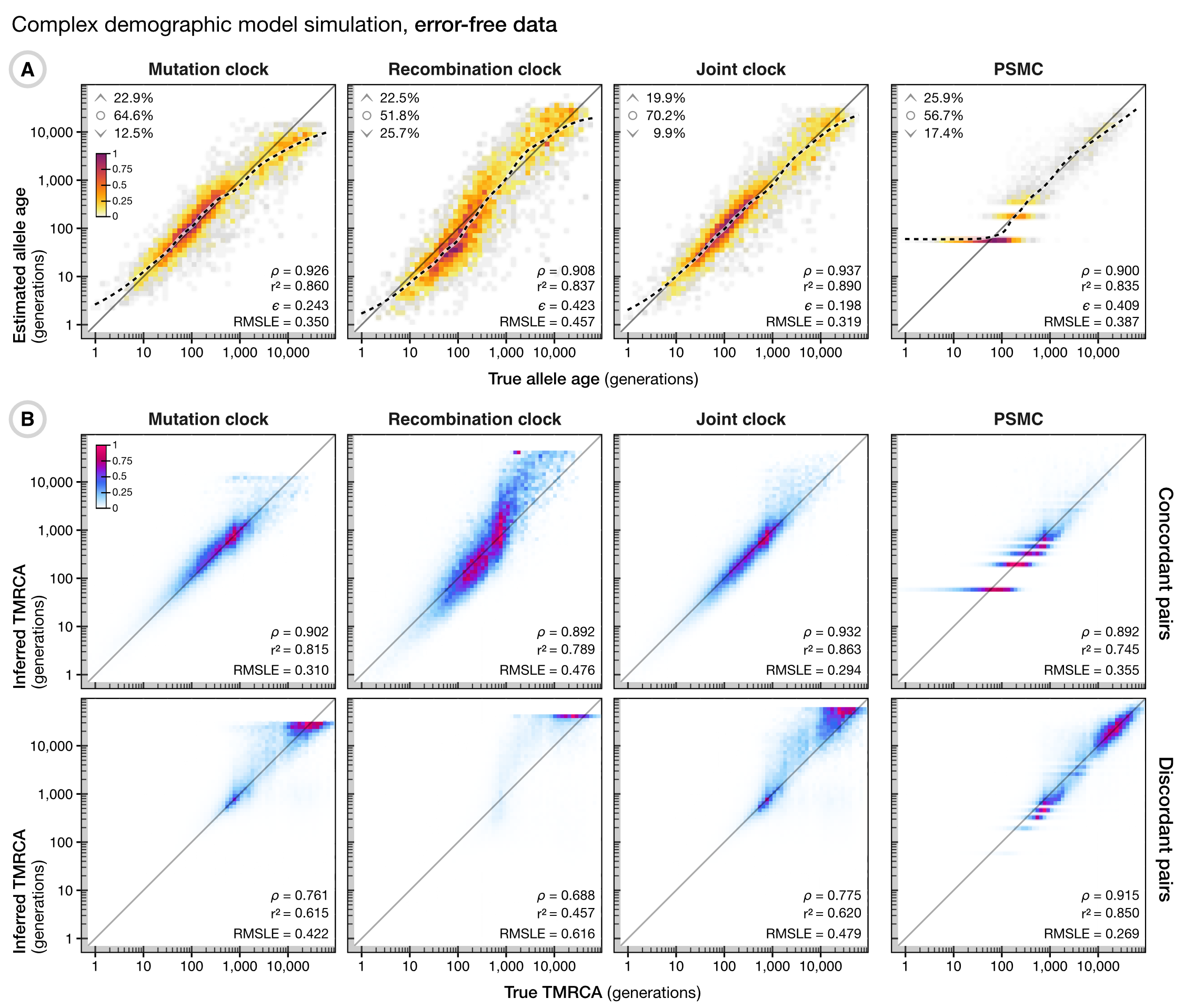

Supplement: S2 Fig — Allele age estimation and TMRCA inference in a simulation that recapitulates the human expansion out of Africa [77], with N = 5,000, Ne = 7,300, constant mutation rate μ = 2.35 × 10^−8^, and variable recombination rates from HapMap (Phase 2, GRCh37) [78] for chromosome 20 (63 Mb). Allele age was estimated for 5,000 variants sampled uniformly from the intersection of sites available at allele count 1 < x < N in data without error and after data were modified with error; see S3 Fig and S4 Fig. Description of plots as in S1 Fig. GRCh37, Genome Reference Consortium Human Build 37; TMRCA, time to the most recent common ancestor. (TIF) [file pbio.3000586.s002.tif]

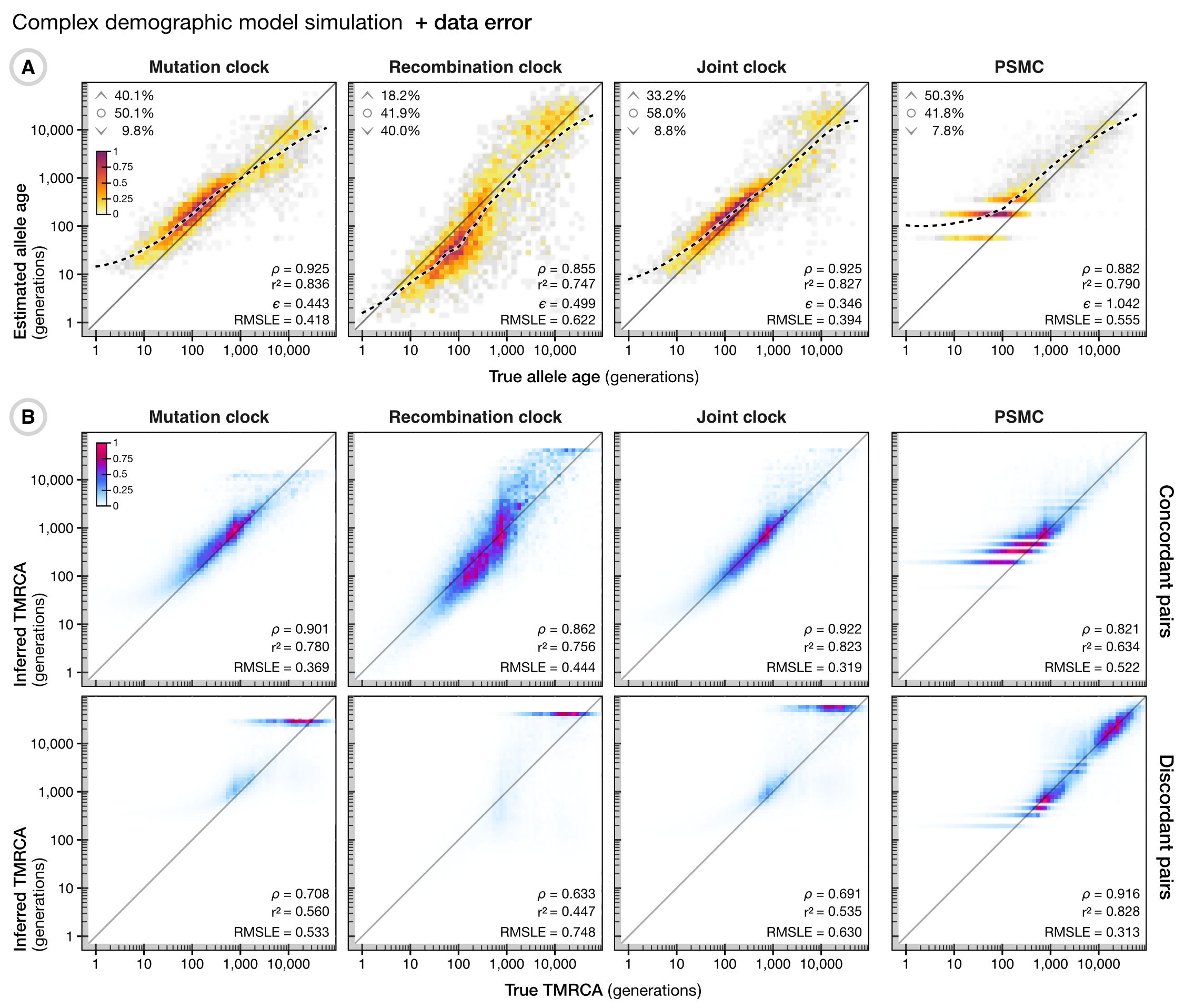

Supplement: S3 Fig — Allele age estimation and TMRCA inference from simulated data in which haplotype data were modified with realistic error rates; calibrated from empirical estimates of genotype errors in TGP data [2], by comparison to corresponding genotype data from the IPG [79]. Allele age was estimated for the same set of 5,000 variants as analyzed in S2 Fig and S4 Fig. Description of plots as in S1 Fig. IPG, Illumina Platinum Genomes Project; TGP, 1000 Genomes Project; TMRCA, time to the most recent common ancestor. (TIF) [file pbio.3000586.s003.tif]

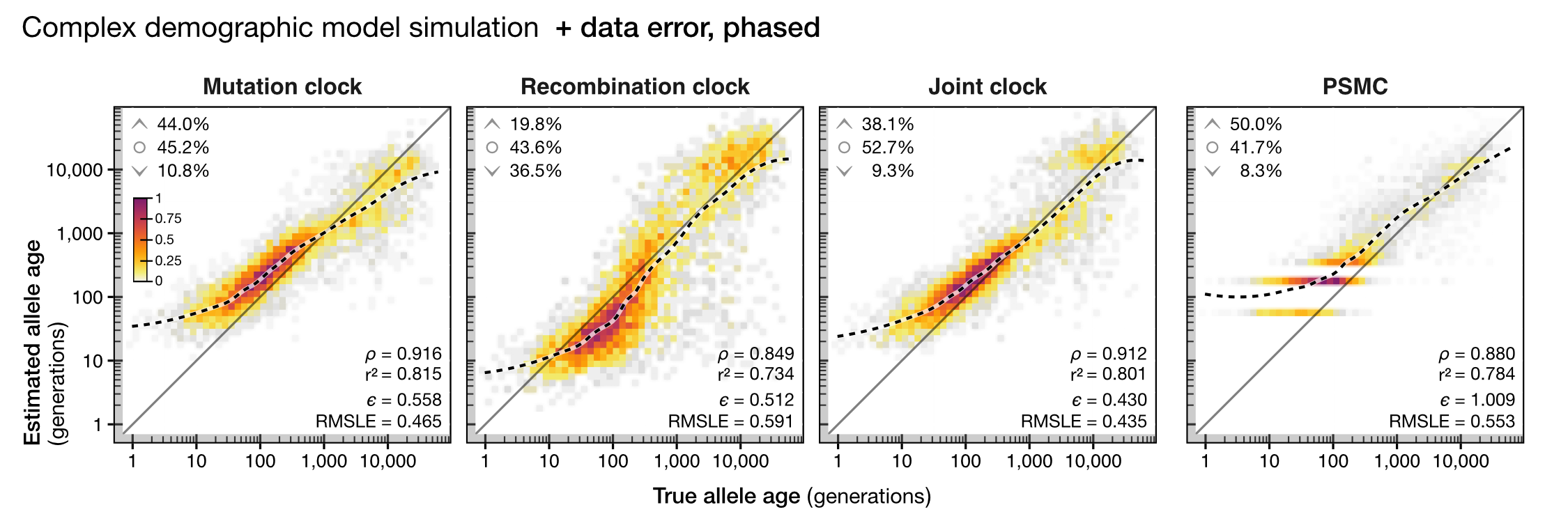

Supplement: S4 Fig — Allele age estimation from simulated data in which haplotype data were modified with realistic error rates, calibrated from empirical estimates of genotype errors in TGP data [2], by comparison to data from the IPG [79]. Haplotype data were additionally phased using SHAPEIT2 [80] after the introduction of data error. Allele age was estimated for the same set of 5,000 variants as analyzed in S2 Fig and S3 Fig. Description of plots as in S1 Fig. Note that the relationship between true and inferred TMRCA per haplotype pair cannot be ascertained conclusively after phasing of haplotype data. IPG, Illumina Platinum Genomes Project; TGP, 1000 Genomes Project; TMRCA, time to the most recent common ancestor. (TIF) [file pbio.3000586.s004.tif]

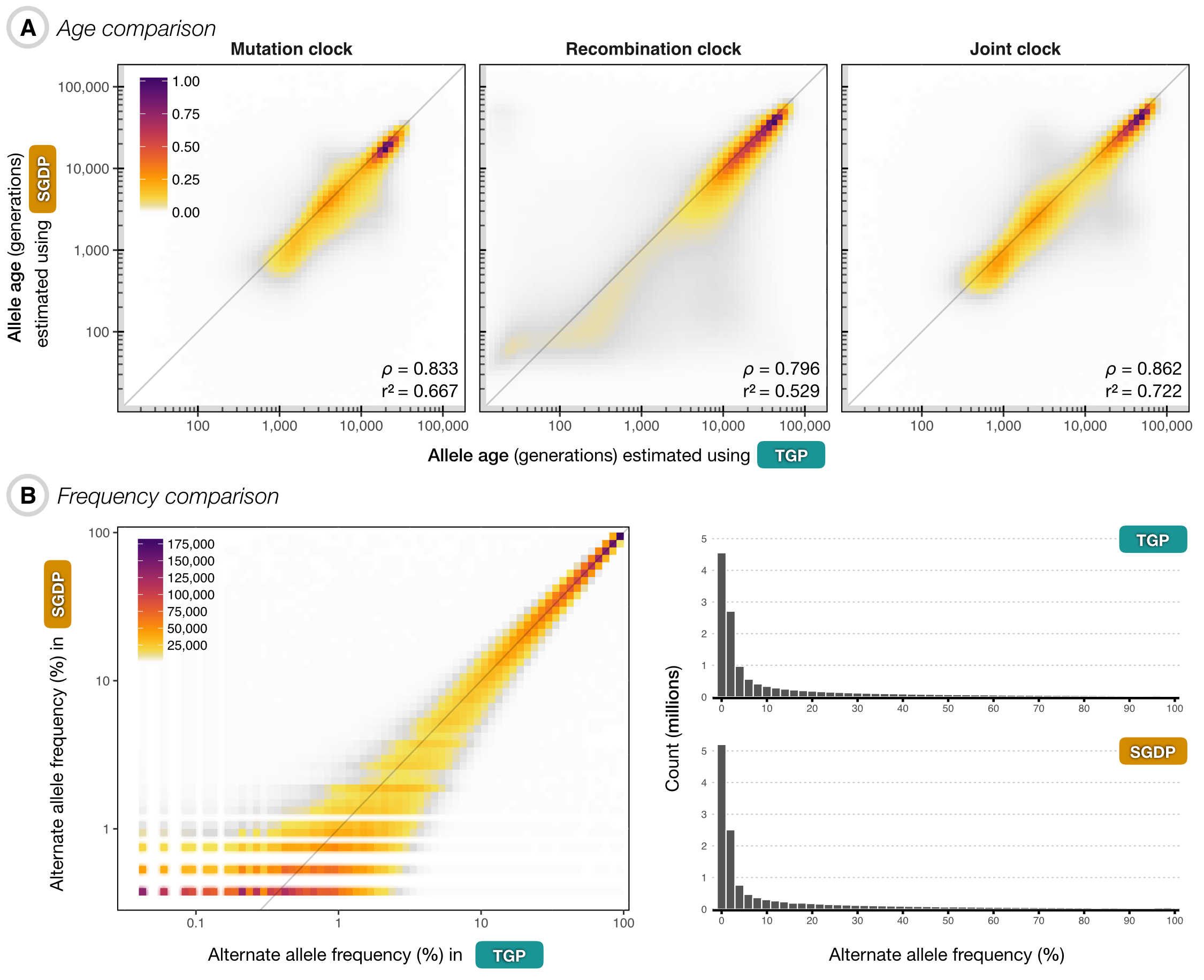

Supplement: S5 Fig — (A) The relationship between allele age using data from the TGP (x axis) and the SGDP (y axis), estimated from the mutation clock (left), recombination clock (center), and the joint clock model (right), for 13.7 million variants dated in both data sources. Colors indicate the density scaled by the maximum per panel. Lower inserts indicate the Spearman rank correlation statistic ρ and the square of the Pearson correlation coefficient (calculated on log-scaled allele ages) r2. (B) Differences in allele frequency of the variants compared (left); the histograms (right) show the frequencies as observed in the TGP (top) and the SGDP (bottom) for corresponding sets of variants. SGDP, Simons Genome Diversity Project; TGP, 1000 Genomes Project. (TIF) [file pbio.3000586.s005.tif]

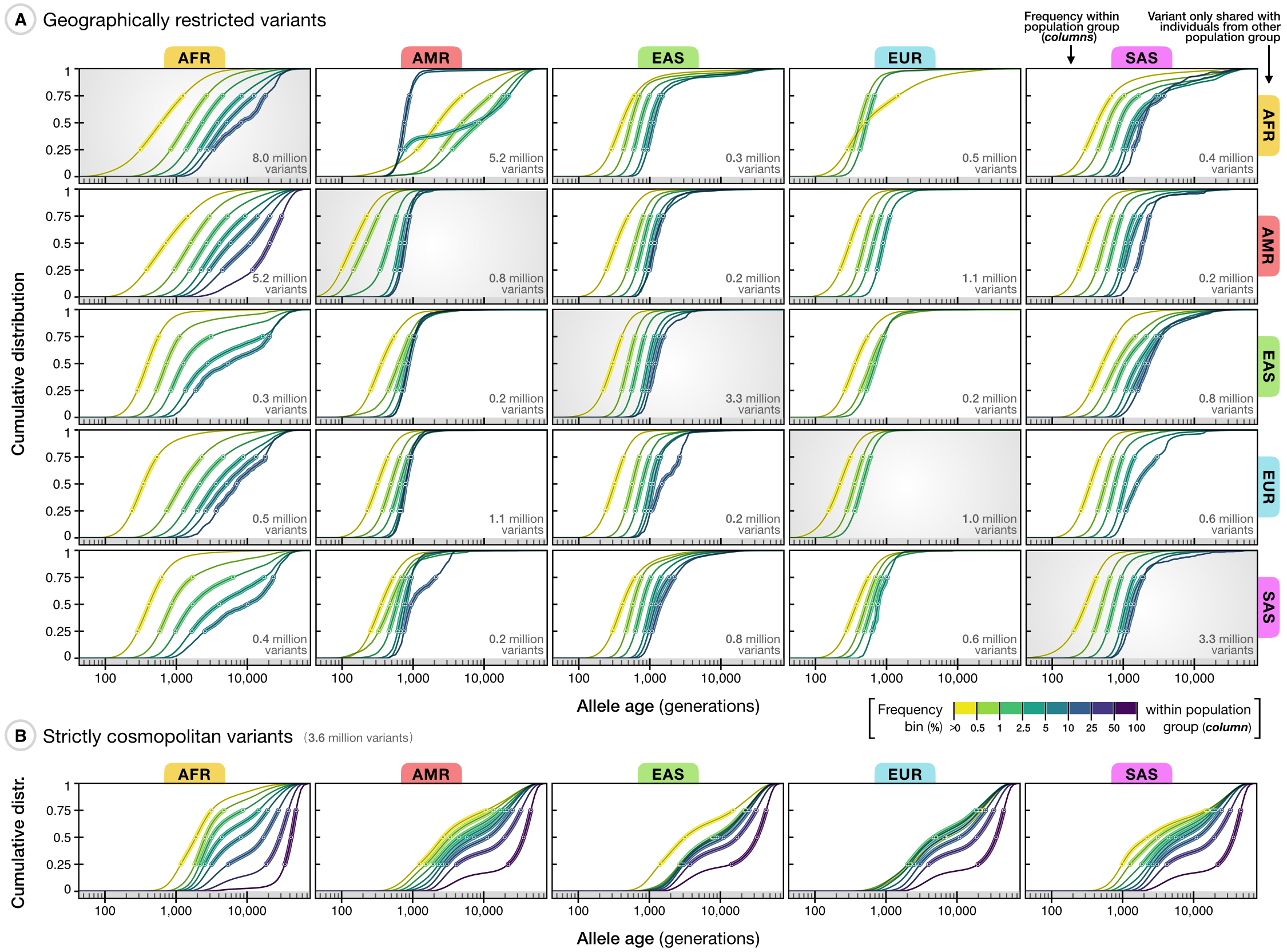

Supplement: S6 Fig — The relationship between allele age and frequency for variants dated in the TGP. Allele age was estimated under the joint clock model using TGP data (whole sample). Of the 43.2 million variants dated in the TGP (chromosomes 1–22), we excluded those with low estimation quality and inconsistent ancestral allele information, which retained 34.4 million variants. Allele frequencies were calculated within subsamples of AFR, AMR, EAS, EUR, and SAS ancestry groups, as defined in TGP sample data. Lines in each panel show the cumulative age distribution of variants within a given frequency bin (see legend), with frequencies as observed within the population group indicated at the top (columns); circles indicate median and interquartile range (25th, 50th, and 75th percentiles). (A) The subset of variants observed in only two population groups, referring to sites that have nonzero frequencies in either of the two populations considered and zero frequency in all other groups (white background color), and geographically restricted variants that are isolated within only a single population group, referring to sites that have nonzero frequencies in the population considered and zero frequency in all other groups (diagonal panels; gray background color). Panels that are diagonal opposites show results for the same set of variants but with frequencies as observed in the population considered (by column). The number of variants retained is shown in each panel (bottom right). (B) The age distribution of strictly cosmopolitan variants (nonzero frequencies in every group) by frequency as seen within a population group. The distributions shown in each panel were obtained on same set of 3,634,716 variants. AFR, African; AMR, American; EAS, East Asian; EUR, European; SAS, South Asian; TGP, 1000 Genomes Project. (TIF) [file pbio.3000586.s006.tif]

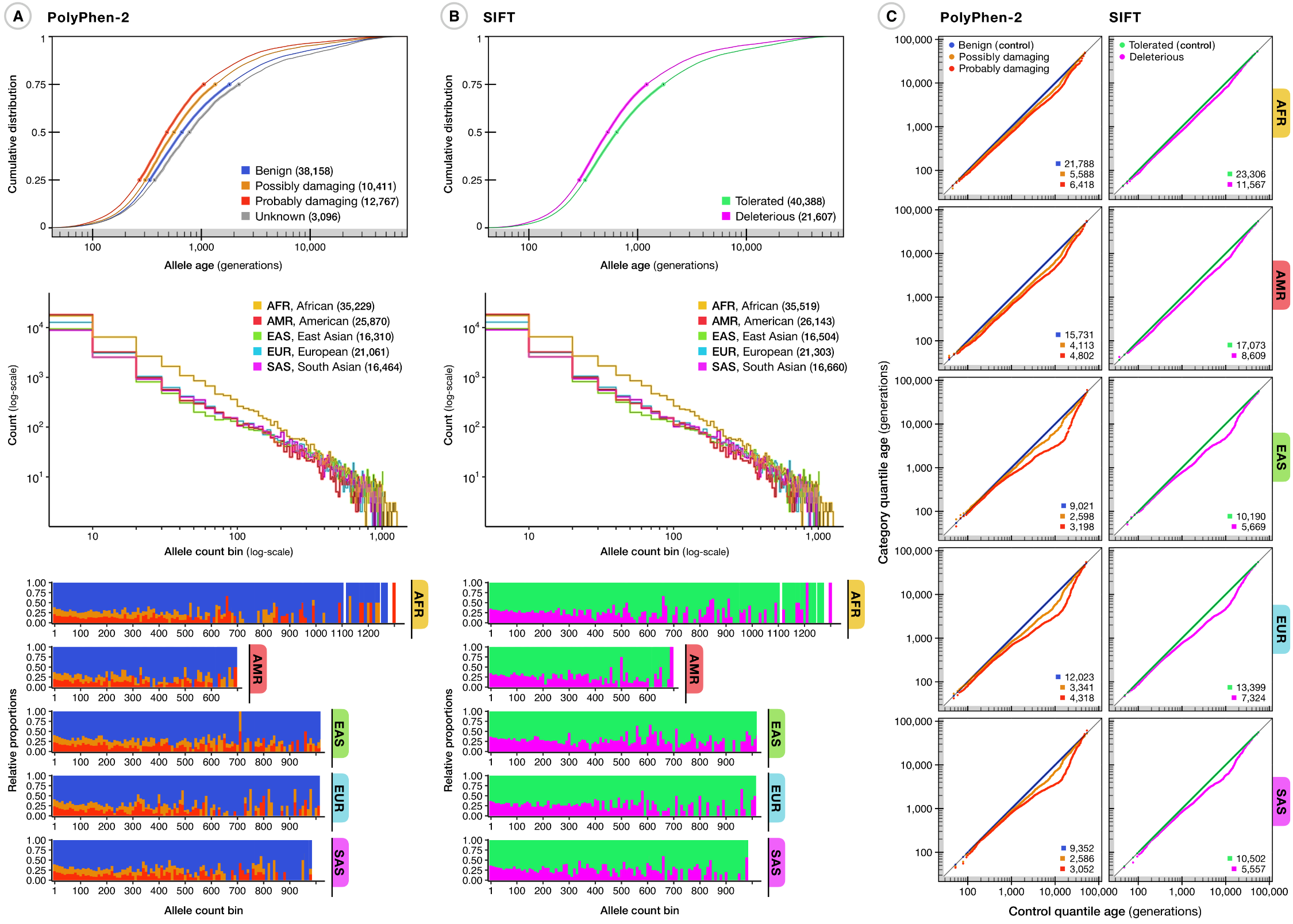

Supplement: S7 Fig — Allele age was estimated (joint clock) using sample data from the TGP for variants annotated by the Ensembl VEP [81]. We excluded variants with low age estimation quality or inconsistent ancestral allelic states (determined through multispecies alignments; information available through Ensembl), which retained 64,432 (of 70,220) variants annotated by PolyPhen-2 [51] and 61,995 (of 67,539) variants annotated by SIFT [52]; of those, 61,615 (of 67,123) variants have been annotated by both methods. (A) The relationship between allele age and variant effects predicted by PolyPhen-2 (top), with effect categories given as benign, possibly damaging, probably damaging, and unknown; variant numbers per category are indicated in the legend. Each line shows the cumulative age distribution by effect category; circles indicate median and interquartile range. The frequency distribution of all variants considered is shown for the 5 major population groups defined in the TGP sample (middle), given as the number of variants within allele count bins (evenly spaced on linear scale but shown on log scale), with allele count as per population group in the TGP. The number of variants at nonzero frequencies in a population group is indicated in the legend. The relative proportion of variants across effect categories per allele count bin for the 5 major population groups in the TGP (bottom). (B) As for part (A), plots show the relationships between allele age and population frequency for variants predicted by SIFT, with effect categories given as tolerated and deleterious. (C) QQ-plots showing differences in allele age distributions for variants annotated by PolyPhen-2 (left) and SIFT (right), compared to a control set of variants (those annotated as benign by PolyPhen-2 or tolerated by SIFT), matched for allele frequency within a given population group. Matching was done by retaining only those variants observed at nonzero frequency within a population group and if variants of every effect [file pbio.3000586.s007.tif]

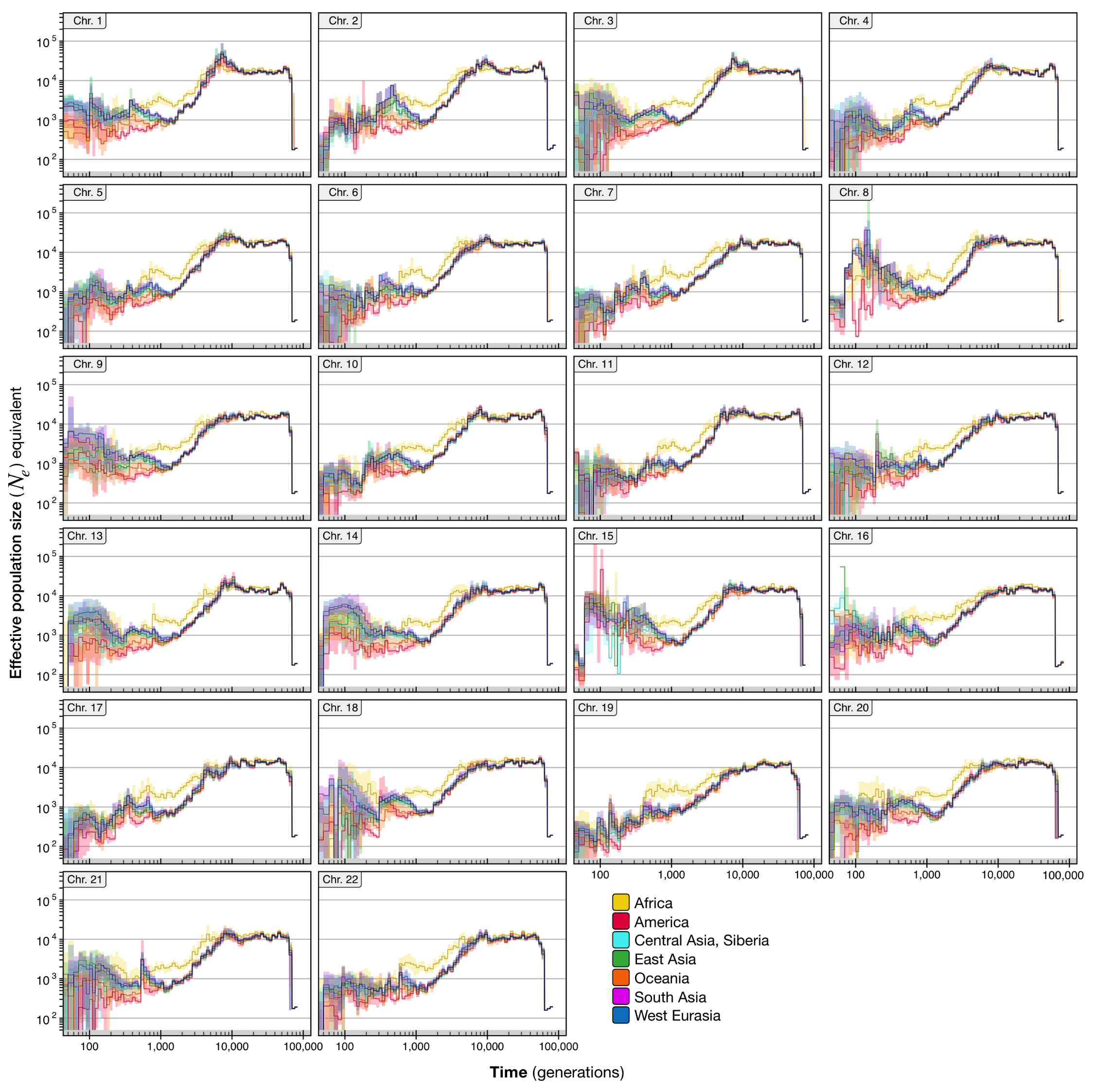

Supplement: S8 Fig — The CCF was inferred for each haploid target genome with all other comparator genomes in the SGDP sample, based on a total of 11.7 million variants dated (joint clock) on chromosomes 1–22, retained after excluding variants with low age estimation quality and inconsistent ancestral allele information. Coalescent intensity was computed per target genome and scaled by the maximum over the sample at a given time interval (epoch; evenly distributed on log scale). Each line shows the median and interquartile range of Ne equivalents inferred for individuals in the different ancestry groups (continental regions; see legend). CCF, cumulative coalescent function; SGDP, Simons Genome Diversity Project. (TIF) [file pbio.3000586.s008.tif]
