# Supplementary material for: Dating genomic variants and shared ancestry in population-scale sequencing data
Source: PLoS Biol. 2020 Jan 17;18(1):e3000586. doi: 10.1371/journal.pbio.3000586 (PMC6992231; doi:10.1371/journal.pbio.3000586)
Supplement: S1 Table — The table shows the total number (Nall) of variants available in the Atlas of Variant Age on chromosomes 1–22, as well as the number of variants dated using data from the TGP alone (NTGP) and the SGDP alone (NSGDP). Additionally, variants present in both data sets were dated using independently inferred pairwise TMRCA results from the TGP and SGDP to obtain a combined age estimate (NCombined). The number of haplotype pairs at which shared haplotype segments and TMRCA were inferred is shown for the two data sources; numbers are shown as the sum of concordant and discordant pairs analyzed per chromosome. See S3 Text for details about the analysis of TGP and SGDP sample data. Full result data sets for each variant, including the results of each pairwise analysis and age estimates obtained under each clock model (mutation, recombination, and joint clock; see S1 Text), are publicly available online at https://human.genome.dating/. SGDP, Simons Genome Diversity Project; TGP, 1000 Genomes Project; TMRCA, time to the most recent common ancestor. (PDF) [file pbio.3000586.s009.pdf]

Chr.	N_{all}	N_{TGP}	N_{SGDP}	N_{Combined}	Haplotype pairs analyzed in **TGP**			Haplotype pairs analyzed in **SGDP**		
					Sum =	Concordant +	Discordant	Sum =	Concordant +	Discordant
1	3,603,845	3,434,810	1,242,878	1,073,843	2,361,753,523 =	670,234,719 +	1,691,518,804	178,223,566 =	57,340,106 +	120,883,460
2	3,909,601	3,727,112	1,354,976	1,172,487	2,552,046,545 =	717,105,655 +	1,834,940,890	193,049,416 =	61,961,663 +	131,087,753
3	3,246,604	3,078,989	1,184,447	1,016,832	2,121,176,886 =	604,531,045 +	1,516,645,841	170,540,962 =	55,202,026 +	115,338,936
4	3,207,690	3,055,978	1,156,613	1,004,901	2,122,460,565 =	616,887,880 +	1,505,572,685	167,838,575 =	55,197,442 +	112,641,133
5	2,936,062	2,777,306	1,054,634	895,878	1,909,734,447 =	541,892,101 +	1,367,842,346	151,025,979 =	48,366,615 +	102,659,364
6	2,841,180	2,703,481	1,023,730	886,031	1,883,434,856 =	551,467,803 +	1,331,967,053	148,183,411 =	48,575,536 +	99,607,875
7	2,651,520	2,530,710	895,609	774,799	1,748,759,380 =	502,479,763 +	1,246,279,617	129,367,159 =	42,116,601 +	87,250,558
8	2,574,597	2,446,808	918,980	791,191	1,681,198,356 =	475,982,722 +	1,205,215,634	131,959,407 =	42,552,249 +	89,407,158
9	1,992,571	1,898,132	695,180	600,741	1,306,395,549 =	371,461,422 +	934,934,127	100,294,995 =	32,686,939 +	67,608,056
10	2,246,011	2,145,420	786,737	686,146	1,484,038,224 =	426,770,806 +	1,057,267,418	114,586,646 =	37,957,269 +	76,629,377
11	2,241,144	2,144,729	758,739	662,324	1,477,906,889 =	421,948,241 +	1,055,958,648	109,601,162 =	35,707,665 +	73,893,497
12	2,152,949	2,051,957	747,059	646,067	1,418,690,735 =	407,634,945 +	1,011,055,790	108,059,559 =	35,361,194 +	72,698,365
13	1,595,057	1,517,081	591,225	513,249	1,052,112,658 =	304,833,376 +	747,279,282	85,982,601 =	28,409,968 +	57,572,633
14	1,477,988	1,406,101	523,596	451,709	970,068,468 =	277,776,895 +	692,291,573	75,600,107 =	24,647,165 +	50,952,942
15	1,351,709	1,287,260	466,764	402,315	885,168,969 =	251,621,540 +	633,547,429	67,128,688 =	21,737,994 +	45,390,694
16	1,501,059	1,431,898	497,060	427,899	976,581,337 =	271,471,963 +	705,109,374	71,332,335 =	22,986,825 +	48,345,510
17	1,290,584	1,230,848	416,535	356,799	840,095,235 =	234,006,858 +	606,088,377	59,898,160 =	19,313,408 +	40,584,752
18	1,275,859	1,212,338	477,771	414,250	838,516,668 =	241,202,386 +	597,314,282	69,122,568 =	22,576,114 +	46,546,454
19	1,028,005	993,091	252,473	217,559	685,952,950 =	196,580,072 +	489,372,878	36,536,015 =	11,914,815 +	24,621,200
20	1,016,303	964,721	363,883	312,301	663,506,829 =	187,934,142 +	475,572,687	52,243,054 =	16,822,189 +	35,420,865
21	628,110	596,192	226,055	194,137	412,235,218 =	118,511,806 +	293,723,412	32,855,224 =	10,805,152 +	22,050,072
22	625,257	597,558	199,880	172,181	413,241,926 =	119,074,286 +	294,167,640	28,956,345 =	9,502,483 +	19,453,862
**Total**	**45,393,705**	43,232,520	15,834,824	13,673,639	**29,805,076,213** =	8,511,410,426 +	21,293,665,787	**2,282,385,934** =	741,741,418 +	1,540,644,516
