# Supplementary material for: Dating genomic variants and shared ancestry in population-scale sequencing data
Source: PLoS Biol. 2020 Jan 17;18(1):e3000586. doi: 10.1371/journal.pbio.3000586 (PMC6992231; doi:10.1371/journal.pbio.3000586)
Supplement: S2 Table — We estimated allele age for variants identified in the TGP to characterize the age distribution of genetic variation across the human genome. Allele age was estimated under the joint clock model. Of the 43,232,520 variants dated in the TGP (chromosomes 1–22), we retained only those at quality score QS > 0.5 (see S1 Text) and at which the ancestral allele is known and mapped to the reference allele (see S3 Text), which retained 34,388,511 variants. The table shows the number of variants (N) and the median of allele age estimates (Q50), as well as the 25th (Q25) and 75th (Q75) percentiles, per continental population group and stratified by allele frequency within that group. This is shown for (A) variants at nonzero frequencies within a given ancestry group, (B) geographically restricted variants that segregate only within a given group, and (C) strictly cosmopolitan variants that are shared among individuals from every continental group. AFR, African; AMR, American; EAS, East Asian; EUR, European; SAS, South Asian; TGP, 1000 Genomes Project. (PDF) [file pbio.3000586.s010.pdf]

Population group	Frequency range (%)	(**A**) Frequency within population				(**B**) Geographically restricted variants				(**C**) Strictly cosmopolitan variants			
		N	Q_{25}	Q_{50}	Q_{75}	N	Q_{25}	Q_{50}	Q_{75}	N	Q_{25}	Q_{50}	Q_{75}
**AFR**	$(0, 0.5]$	8,933,432	351	670	1,287	5,140,255	309	607	1,244	476,121	1,169	1,902	3,155
	$(0.5, 1.0]$	2,699,024	886	1,558	2,857	1,536,213	826	1,439	2,643	191,398	1,840	2,574	4,732
	$(1.0, 2.5]$	3,200,737	1,497	2,568	5,247	1,081,782	1,362	2,332	4,467	293,687	2,158	3,156	8,779
	$(2.5, 5.0]$	2,186,932	2,270	4,128	10,568	246,828	2,117	3,748	8,313	338,261	2,533	4,417	14,638
	$(5.0, 10]$	1,826,818	3,055	6,692	16,831	31,552	2,694	5,072	12,363	513,291	3,197	8,176	19,530
	$(10, 25]$	1,681,460	5,225	15,137	25,749	805	3,290	8,142	17,928	919,982	5,431	17,092	27,615
	$(25, 50]$	694,784	17,662	28,246	37,593	0	–	–	–	604,276	18,542	29,019	38,187
	$(50, 100]$	300,996	33,911	41,749	48,871	0	–	–	–	297,700	34,049	41,845	48,941
**AMR**	$(0, 0.5]$	8,124,067	407	1,125	3,186	634,975	97	145	222	105,375	1,237	2,684	10,711
	$(0.5, 1.0]$	1,920,838	823	2,390	8,016	87,768	144	218	321	110,129	1,539	3,367	13,708
	$(1.0, 2.5]$	1,861,646	1,117	3,217	13,840	32,732	340	454	584	362,381	1,937	4,488	17,220
	$(2.5, 5.0]$	827,543	1,460	3,597	16,886	13,699	554	662	801	430,222	2,323	5,750	19,605
	$(5.0, 10]$	784,043	2,076	4,897	19,281	6,011	642	751	896	602,870	2,614	7,301	21,384
	$(10, 25]$	1,032,974	2,885	10,238	24,772	750	667	773	887	981,035	3,049	11,263	25,301
	$(25, 50]$	663,726	4,157	19,824	33,473	1	–	–	–	662,766	4,166	19,850	33,488
	$(50, 100]$	379,938	22,142	36,490	46,057	0	–	–	–	379,938	22,142	36,490	46,057
**EAS**	$(0, 0.5]$	4,452,108	257	377	694	2,419,536	219	299	402	588,313	1,417	3,175	15,178
	$(0.5, 1.0]$	830,225	441	580	956	473,651	400	496	620	127,379	2,677	7,679	21,953
	$(1.0, 2.5]$	863,803	665	949	3,175	290,434	556	678	838	246,062	2,753	7,939	22,488
	$(2.5, 5.0]$	566,841	1,124	2,521	12,564	59,809	789	944	1,167	295,616	2,849	8,469	22,505
	$(5.0, 10]$	581,311	2,056	4,592	19,164	9,022	885	1,061	1,344	437,605	2,885	9,123	23,237
	$(10, 25]$	880,951	2,817	9,193	24,394	404	966	1,205	1,581	820,256	3,063	11,130	25,395
	$(25, 50]$	654,316	3,700	17,149	31,826	5	789	826	829	650,087	3,740	17,325	31,924
	$(50, 100]$	469,485	14,392	32,707	44,104	1	–	–	–	469,398	14,411	32,711	44,106
**EUR**	$(0, 0.5]$	5,501,819	272	488	2,002	952,272	152	221	309	236,433	2,244	6,848	21,733
	$(0.5, 1.0]$	1,000,000	463	681	1,381	78,287	272	356	465	131,350	2,031	5,102	19,648
	$(1.0, 2.5]$	1,152,780	752	1,138	3,489	11,207	357	458	593	314,046	2,005	4,688	18,342
	$(2.5, 5.0]$	776,938	1,148	2,421	11,344	46	535	720	989	401,255	2,167	5,045	18,692
	$(5.0, 10]$	747,466	1,985	4,451	18,440	0	–	–	–	566,483	2,500	6,629	21,036
	$(10, 25]$	1,000,157	2,885	9,925	24,724	0	–	–	–	940,136	3,010	10,874	25,295
	$(25, 50]$	657,062	3,998	18,937	32,945	0	–	–	–	654,305	4,011	19,001	32,986
	$(50, 100]$	390,799	19,765	35,465	45,555	0	–	–	–	390,708	19,779	35,469	45,558
**SAS**	$(0, 0.5]$	5,608,105	274	428	737	2,193,373	199	295	418	136,412	982	2,245	15,009
	$(0.5, 1.0]$	1,481,990	474	689	1,188	668,101	385	502	655	124,372	1,277	3,114	15,916
	$(1.0, 2.5]$	1,359,096	770	1,167	3,219	381,602	589	740	946	292,606	1,861	4,417	18,160
	$(2.5, 5.0]$	777,344	1,374	2,839	12,598	77,540	892	1,088	1,427	389,781	2,337	5,594	19,259
	$(5.0, 10]$	764,143	2,346	5,265	18,799	13,221	1,027	1,273	1,837	613,616	2,636	6,868	20,621
	$(10, 25]$	1,046,826	3,018	10,510	24,510	513	1,135	1,415	1,929	1,017,345	3,067	10,952	24,770
	$(25, 50]$	676,707	4,194	19,501	33,013	5	1,467	1,492	2,063	676,430	4,197	19,507	33,016
	$(50, 100]$	384,158	22,014	36,446	46,031	0	–	–	–	384,154	22,015	36,446	46,031
