# Supplementary material for: Dating genomic variants and shared ancestry in population-scale sequencing data
Source: PLoS Biol. 2020 Jan 17;18(1):e3000586. doi: 10.1371/journal.pbio.3000586 (PMC6992231; doi:10.1371/journal.pbio.3000586)
Supplement: S1 Text — (PDF) [file pbio.3000586.s014.pdf]

S1 Text

Dating of genomic variants and shared ancestry in population-scale sequencing data

Patrick K. Albers[1*] and Gil McVean[1]

[1]Big Data Institute, Li Ka Shing Centre for Health Information and Discovery, University of Oxford, United Kingdom

*patrick.albers@bdi.ox.ac.uk

Contents

1 Genealogical approach . . . 2
2 Sampling of haplotype pairs . . . 3
3 Inference of pairwise TMRCA . . . 5
3.1 Mutation clock model . . . 6
3.2 Recombination clock model . . . 8
3.3 Joint clock model . . . 10
4 Composite posterior estimation of variant age . . . 11
5 Heuristic method to reject outlier pairs . . . 12
5.1 Quality score . . . 13

Genealogical estimation of variant age (GEVA)

Here we introduce our methodology to estimate the age of genetic variants; the point in time when a mutation gave rise to the allele observed at a particular locus in sample data. In principle, we can estimate the age of any variant segregating at any frequency in a population, without being affected by the selective forces that acted on the allele. Our method has several useful properties:

- It does not require a demographic model or assumptions about relatedness among sampled individuals. Parametric models are used within the approach to detect recombination breaks, account for error, and obtain a posterior distribution on the time to the most recent common ancestor (TMRCA) for pairs of haplotypes, but the underlying approach to estimate allele age is agnostic with respect to the genealogical process.
- It makes full use of the information available in whole genome sequencing data, combining information from both the mutation and recombination clocks inherent in population genetic data.
- It is scalable. By sampling pairs of individuals, the computational costs can be limited, with little loss of power. For example, the probability of sampling the deepest root within the coalescent tree of a population in a subsample of size n is approximately $(n+1)/(n-1)$, suggesting that the most recent common ancestor (MRCA) of a subsample usually captures the MRCA of the larger sample. The probability of capturing the nearest discordant clade is more dependent on sample size (and the true age of the variant).

- It is robust to errors. Real data has sequencing or genotyping error, as well as haplotype phasing error, which can create problems in identifying haplotypes that carry a variant of interest and may create false breaks in haplotypes. We use empirically calibrated models of genotype error, which we measured in sequencing data, and we use filters to identify outliers in TMRCA distributions. The approach also makes the algorithm robust to low levels of recurrent mutation.
- It can combine information from different data sources. The core algorithm within GEVA combines information from many pairwise comparisons around a variant of interest. The comparisons can be performed across many data sets, potentially even distributed ones, with the only data needing to be shared being the parameter values of the pairwise posterior TMRCA distributions.

We refer to our method as the genealogical estimation of variant age (GEVA), which we developed as an integrated, analytical framework. We implemented GEVA in `C++` and made the source code available online.*

1 Genealogical approach

Our goal is to estimate the age of an allele at target site k, of which there are x_k copies in a sample of size N haploid chromosomes. We assume that a mutation occurred only once at site k in the history of the population and that there was no back-mutation. The allele is therefore assumed to derive from a mutation event in the genome of the common ancestor of the chromosomes that carry the allele. We assume that we know the ancestral and derived allelic states with certainty and that haplotypes have been phased.

We divide the sample into two disjoint subsets, X_k and Y_k, consisting of carrier and non-carrier haplotypes, respectively. By tracing back the ancestry of the chromosomes in X_k, we expect that all of them share a common ancestor by the time of the focal mutation event and that the mutation occurred before any of them share a common ancestor with a chromosome in Y_k.

The genealogy of the sample at site k is generally unknown. However, we can identify pairs of chromosomes whose lineages have a common ancestor either before or after the time of the focal mutation event.

- **Concordant pairs.** Any two carrier haplotypes are expected to coalesce more recently than the time of the focal mutation event. Specifically, they will have coalesced by the time of the node in the tree below where the mutation occurred.
- **Discordant pairs.** Any pair composed of one carrier and one non-carrier haplotype is expected to coalesce further back in time, prior to the time of the focal mutation event. Specifically, they will coalesce at or after the time of the node in the tree above where the mutation occurred.

---
* https://github.com/pkalbers/geva

Informally, the time of mutation is delimited by two time points; the time the subtree below the mutation has coalesced into a single lineage of the most recent common ancestor (MRCA) carrying the derived allele, and the time this subtree coalesced with the remaining sample. Note that we require $x_k > 1$ carrier haplotypes to form at least one concordant pair and, likewise, $x_k < N$ to form at least one discordant pair. While in principle it would be possible to use only disconcordant pairs to approximate an upper bound of allele age, for example, if only one chromosome carries the allele (singletons), or only concordant pairs to approximate a lower bound if all chromosomes carry the allele, we did not consider such cases here.

2 Sampling of haplotype pairs

The numbers of concordant and discordant pairs grow quadratically with sample size (for a fixed allele frequency), which can be computationally prohibitive. The set containing all possible concordant pairs that can be formed for a given target allele at site k is given by

$$\mathcal{C}_k = \big\{\{i,j\} : \ i,j \in X_k,\ i \neq j\big\} \tag{1}$$

and the set containing all possible discordant pairs is given by

$$\mathcal{D}_k = \big\{\{i,j\} : \ i \in X_k,\ j \in Y_k\big\}\ , \tag{2}$$

which are subsets of $N(N-1)/2$ possible pairs in the sample. There are $|\mathcal{C}_k| = x_k(x_k-1)/2$ possible concordant pairs and $|\mathcal{D}_k| = x_k(N-x_k)$ discordant pairs. We use two different sampling strategies to limit the computational cost while maintaining accuracy (described below). The maximum number of pairs sampled per group are user-defined parameters;

$$\begin{aligned} &\max\nolimits_{\mathcal{C}} \quad \text{for concordant pairs, and} \\ &\max\nolimits_{\mathcal{D}} \quad \text{for discordant pairs.} \end{aligned}$$

Concordant pairs. We sample concordant pairs uniformly at random. The probability that a subsample of X_k includes at least one pair that spans the TMRCA for the subtree depends on the number of descendants of the two branches leading to the MRCA. In a neutral model, this partition is uniform, hence for large sample size, a random draw of a chromosomes from X_k will include the MRCA with probability of at least $(a-1)/(a+1)$. This implies that a random sample of concordant pairs will include the MRCA of the samples that carry the variant with high probability. Examples of the distribution of pairwise TMRCA distributions for variants at different frequencies are shown in the figure on Page 4.

Discordant pairs. Our approach to sample discordant pairs is based on prioritizing non-carrier haplotypes that are the nearest genealogical neighbors to the subtree below the

mutation. By the time the subsample of carrier haplotypes has merged into a single lineage, the subsample of non-carrier haplotypes will have collapsed into an unknown number of ancestral lineages, which may also collapse further before joining with the remaining lineage. Dependent on the age of the allele and the ancestral background of the sample, the number of distinct nodes at which discordant pairs coalesce in the tree above the mutation can be relatively small; see figure below for examples. A randomly formed subset of discordant pairs would likely capture a large proportion of pairs that coalesce at a node close to or at the MRCA of the sample.

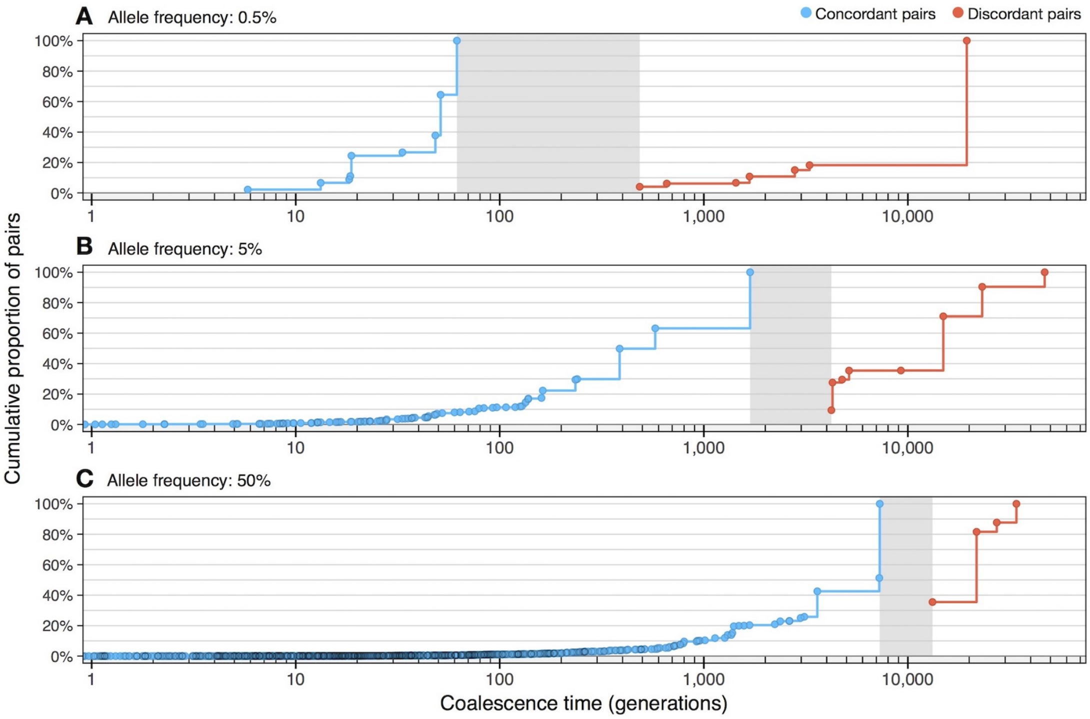

Distribution of coalescence times for concordant and discordant pairs. Coalescence time distributions for concordant and discordant pairs at three randomly selected sites in simulated data with frequencies of (A) 0.5%, (B) 5%, and (C) 50%. Data were simulated with sample size of 1,000 haplotypes, N_e=10,000, $\mu = 1 \times 10^{-8}$, and $r = 1 \times 10^{-8}$; using *Script 1* from **S2 Text**. Each panel shows the cumulative fraction of pairs (*y-axis*) that have coalesced back in time (*x-axis*); shown separately for pairs of concordant (*blue*) and discordant (*red*) haplotypes. Areas in *gray* indicate the branch in the underlying genealogical tree on which the focal mutation arose, delimited by the maximum and minimum of the coalescence times of concordant and discordant pairs, respectively.

To maximize the chance of selecting the most recent discordant coalescent events, we use a heuristic to identify candidates. Specifically, we compute the Hamming distance for the set of possible pairs, measured by counting allelic mismatches between two sequences at a fixed interval to both sides relative to a given target site. Here, we scanned up to the first 5,000 positions on each side (as seen in the data). The set of pairs is sorted from low to high distance to form a priority queue. We additionally "relax" priority ranks by scanning each position in the queue, starting at the lowest distance, to remove pairs in which the same non-carrier haplotype appears more than once in consecutive order. Removed pairs are then randomly inserted at a lower rank in the queue before continuing at the next position.

This is done, because the ranking of pairs based on their Hamming distance alone is limited in that the sequence interval at which pairs are compared is unlikely to be confined to the local genealogy, but rather may involve sequence variation that derived from peripheral genealogies. Also, a distinction of haplotypes predicated on carrier and non-carrier status is biased at target sites that violate model assumptions or in presence of data error. By relaxing the priority rank, we attempt to reduce the chance to include false negative non-carrier haplotypes that would otherwise be preferentially selected.

3 Inference of pairwise TMRCA

There are two main sources of information that relate to the time separating two haplotypes from their MRCA. Mutation events occur independently in each lineage and accumulate along the sequence as the ancestral haplotype is passed on over generations, and recombination events break down the length of an ancestral haplotype independently in each lineage in each generation; see schematic below. Here we describe three coalescent-based "clock" models, which are constructed in a Bayesian setting for probabilistic inference of the TMRCA between two lineages, and where time is modeled given information about mutational differences (*mutation clock*), recombination distance (*recombination clock*), or both (*joint clock*).

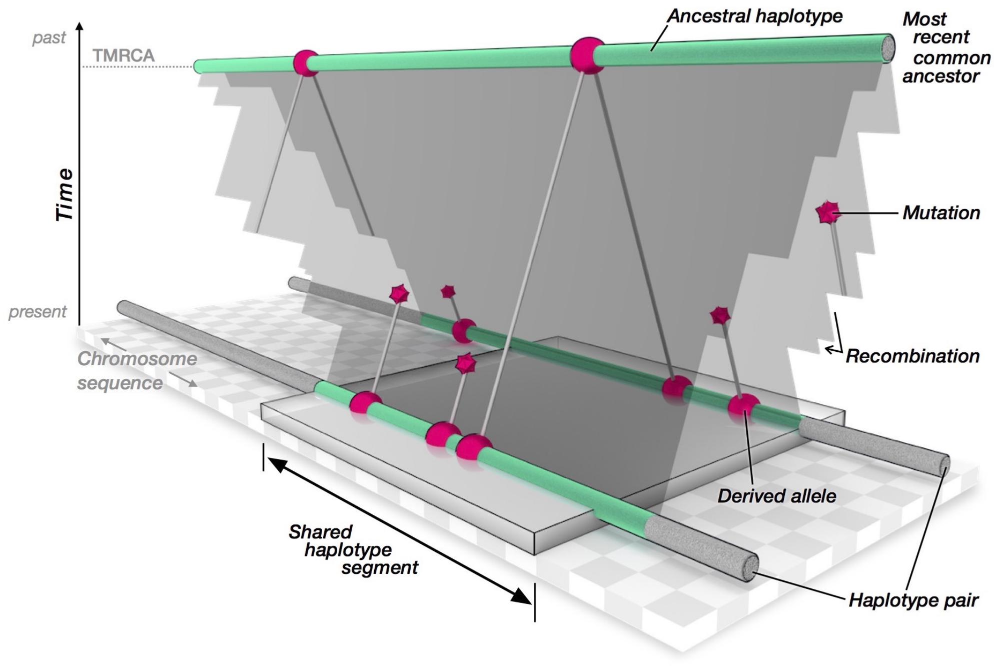

Schematic of the genealogical relationship between two random haplotypes. A random pair of haplotypes (*bottom*) share a common ancestor in the past (*top*), from whom they have inherited some piece of DNA. Over time, the ancestral haplotype sequence (*green*) has been broken down due to recombination, which occurred independently in each lineage; indicated by *cuts* in the plane connecting the two current haplotypes to their MRCA. A shared haplotype segment is locally defined as the sequence interval both haplotypes retained since inheritance from the MRCA; indicated by the *gray* block. Mutation events (*red polygons*) occurred independently in either lineage following the ancestral split from the MRCA. By using existing knowledge about the rate at which mutation and recombination events occur, it is possible to infer the time to the most recent common ancestor (TMRCA) from sequence information at the shared haplotype segment.

We define the continuous random variable T for the time of coalescence, where $T = t$ takes values scaled in units of the diploid effective size of the population, N_e, such that

$$t = \frac{m}{2N_e} \tag{3}$$

where m is the number of generations (meioses). We use the neutral coalescent to characterize the prior probability of coalescence;

$$\pi(t) \propto e^{-t}. \tag{4}$$

That is, the waiting time until the first coalescent event for two randomly sampled lineages is approximately exponentially distributed, with rate equal to 1. The choice of prior is expected to have a weak effect on the inference of TMRCA (and subsequent estimations of allele age), but depends on the order of mutation and recombination rates. For example, as the mutation rate gets large, the approach will converge on the true TMRCA irrespective of the prior. In theory, however, it would be possible to use different distributions, or estimate the prior from the data.

The setting in which each clock model operates is as follows. For a given pair of haplotypes, we treat the region they share by descent around a given focal site as known. More specifically, we assume that the genomic locations of the breakpoints that delimit the shared haplotype segment are known. We therefore assume that no recombination has occurred within the shared sequence interval in either of the two lineages considered. While this is purely theoretical, in **S2 Text** we propose a solution to locally infer the shared haplotype segment around a given target site, which employs a hidden Markov model (HMM) to infer the nearest breakpoints of past recombination events between two haplotype sequences.

3.1 Mutation clock model

Following the assumptions of the infinite-sites model (ISM), mutations occur only once at each site in the history of the sample, without recurrent or back-mutations [1, 2]. It follows that the number of pairwise differences observed between two non-recombining DNA sequences is equal to the number of mutation events that occurred on both lineages since coalescence in the MRCA. The number of pairwise differences is equivalent to the number of segregating sites in a sample of two haplotypes. We further assume that all mutations in the region are observed in the data and that alleles are encoded as 0s and 1s to distinguish ancestral and derived allelic states, respectively. That is, we know the ancestral and derived states for the variant at a given locus.

Given the compound mutation parameter $\theta = 4N_e\mu$, where μ is the known mutation rate per base pair per generation, mutations accumulate on each lineage independently as a Poisson process with rate $\theta/2$. We model the number of pairwise differences using the discrete random variable S, which follows the Poisson distribution with parameter θht, where t is the population-scaled time parameter and h is the physical length of the shared haplotype

segment considered, measured as the number of basepairs that make up the segment. We obtain the number of pairwise differences as the sum of allelic mismatches observed along the sequence interval. The probability to observe $S = s$ pairwise differences is given by the probability mass function (PMF) of the Poisson distribution, namely

$$P(S = s \mid \theta h t) \; = \; \frac{(\theta h t)^s}{s!} e^{-\theta h t} \,. \tag{5}$$

The likelihood function for the time parameter t is proportional to the above, but requires only those terms that involve t and where constant terms can be dropped, such that

$$\mathcal{L}(t \mid \theta, h, s) \; \propto \; t^s e^{-\theta h t} \tag{6}$$

from which we obtain the posterior probability of the time of coalescence as

$$\begin{aligned} p(t \mid \theta, h, s) \; &\propto \; \mathcal{L}(t \mid \theta, h, s) \; \times \; \pi(t) \\ &\propto \; t^s e^{-t(\theta h + 1)} \,. \end{aligned} \tag{7}$$

In the above, the density of the posterior probability is specified up to a missing normalising constant. The form of Equation (7) implies a Gamma distribution with shape (α) and rate (β) parameters

$$\alpha = s + 1 \,, \;\; \beta = \theta h + 1 \tag{8}$$

such that the posterior density can now be written as

$$p(t \mid \theta, h, s) \; = \; \frac{(\theta h + 1)^{s+1}}{\Gamma(s + 1)} t^s e^{-t(\theta h + 1)} \,. \tag{9}$$

This result has been obtained previously, for example see [3, Eq. 3.45]. Note that we previously defined the prior using the exponential distribution with rate equal to 1, but which is equivalent to using the Gamma distribution with $\alpha = 1$ and $\beta = 1$, due to $T \sim \mathrm{Exp}(\lambda)$ being equivalent to $T \sim \mathrm{Gamma}(1, \lambda)$, such that the prior distribution is conjugate to the posterior given above.

Variable mutation rates. In applications to genomic data with considerable heterogeneity of mutation rates, the model can be adjusted to consider variable rates along the genome, if such data is available. Let ϑ denote the expected value of pairwise differences over the shared haplotype segment per unit of population-scaled time. We have $\vartheta = \theta h$ if the mutation rate is uniform (as is assumed above). Otherwise, given a vector of known mutation rates per site, we compute $\vartheta = 4N_e \sum_{k=1}^{h} \mu_k$, where μ_k is the per generation mutation rate at the kth site in the focal nucleotide sequence of length h. This is used to calculate the rate of the Gamma distribution as $\beta = \vartheta + 1$.

Conditional count of pairwise differences. According to ISM assumptions, the number of pairwise differences observed along a non-recombinant region in two focal haplotype sequences is equal to the number of mutation events that occurred since their MRCA. However, this assumption is readily violated in applications to real (non-simulated) data; for example, due to recurrent or back-mutations, flip errors in phased haplotype data, and generally in presence of data error when alleles have been missed or falsely identified in the sequencing or genotyping process. Our model is sensitive to ISM violations, because every allelic mismatch is counted as a mutation event that separates the two focal haplotypes from their MRCA.

To account for departures from model assumptions, we exclude sites conditional on the frequency of the allele whose age we attempt to estimate. Let f_k denote the frequency of the derived allele at a given target site k. Pairwise differences are counted by scanning along the shared haplotype region of the two sequences considered. At the ith site in the sequence, a mismatch is counted if $f_i \leq f_k$ or excluded otherwise. The number of pairwise differences is thereby restricted to alleles that conform to ISM assumptions; that is, mutations that occurred more recently than the focal mutation event at site k.

We apply this restriction to concordant pairs, as both haplotypes carry the focal allele and are expected to coalesce before the time of the focal mutation event. But it does not apply to discordant pairs, because we do not know the actual number of haplotypes in the sample that subtend the lineage at which a pair of carrier and non-carrier haplotypes join back in time. If we would restrict the count of pairwise differences in discordant pairs, inferred coalescent times are likely to be underestimated, which may likewise affect estimates of allele age. However, we expect that overestimation at discordant pairs is less problematic as it is unlikely that false allelic mismatches are equally replicated among all pairs considered. Dependent on the age of the focal allele, we may also expect that the shared haplotype segment at a discordant pair will be relatively short, as there has been more time for recombination to break down its length, thereby reducing the chance to encounter sites that violate model assumptions.

3.2 Recombination clock model

The length of a haplotype segment shared between two sequences is delimited by two recombination events (meiotic crossovers) that occurred independently at some point in the past in either of the two lineages considered. Relative to a given target site in the genome, we characterize the surrounding haplotype segment by the two points at which the pairwise ancestral relationship changes due to recombination. To be precise, we define a *breakpoint* as the first site along the sequence that immediately follows the point at which the ancestral haplotype recombined in either of the two lineages; independently on the left and right-hand side from the target position. The full length of the focal shared haplotype is thereby enclosed by the breakpoint interval.

We use the compound recombination parameter $\rho = 4N_e r$, where r is the recombination rate per site per generation. In either direction from the target site, the genetic distance to the first recombination event is exponentially distributed with parameter ρt, where t is the population-scaled time of coalescence. We define D as a random variable for the distance along the sequence of a haploid individual. The probability to observe recombination at distance d is therefore

$$P(D = d \mid \rho t) \;=\; \rho t \, e^{-\rho t d} \tag{10}$$

and the probability that recombination occurred farther beyond along the sequence is

$$P(D > d \mid \rho t) \;=\; e^{-\rho t d} \,. \tag{11}$$

However, because recombination occurred independently along either of the two sequences considered, where only the nearest event defines a breakpoint, it follows that

$$\begin{aligned} P(D_2 = d \mid \rho t) \;&=\; 2 \times P(D = d) \times P(D > d) \\ &=\; 2\rho t \, e^{-2\rho t d} \end{aligned} \tag{12}$$

where D_2 denotes the breakpoint distance involving two sequences, either of which breaks first. In cases where no breakpoint is encountered before reaching the end of the chromosome, it is implied that no recombination occurred along either sequence, such that

$$P(D_2 > d \mid \rho t) \;=\; P(D > d)^2 \;=\; e^{-2\rho t d} \,. \tag{13}$$

To combine Equations (12) and (13), we can write

$$f_{D_2}(d \mid \rho t, b) \;=\; (2\rho t)^b \, e^{-2\rho t d} \tag{14}$$

where $b = 1$ if a breakpoint was found or $b = 0$ otherwise. The above can be further extended to consider the breakpoint distances on both sides, d_L and d_R, such that the full length of the shared haplotype segment, h, is observed with probability

$$\begin{aligned} f_H(h) \;&=\; (2\rho t)^{b_L} \, e^{-2\rho t d_L} \;\times\; (2\rho t)^{b_R} \, e^{-2\rho t d_R} \\ &=\; (2\rho t)^{b_L + b_R} \, e^{-2\rho t h} \end{aligned} \tag{15}$$

where $b_L, b_R \in \{0, 1\}$ indicate the breakpoints on the left and right-hand side from the focal position. The likelihood function for t can now be obtained from Equation (15) by ignoring multiplicative constants, such that

$$\mathcal{L}(t \mid \rho, h, b_L, b_R) \;\propto\; t^{b_L + b_R} \, e^{-2\rho h t}, \tag{16}$$

to obtain the posterior probability of coalescence time as

$$\begin{aligned} p(t \mid \rho, h, b_L, b_R) \;&\propto\; \mathcal{L}(t \mid \rho, h, b_L, b_R) \;\times\; \pi(t) \\ &\propto\; t^{b_L+b_R}\, e^{-t(2\rho h+1)}\,. \end{aligned} \tag{17}$$

The above has the same form as the posterior probability derived for the mutation clock model; see Equation (7), Section 3.1. Thus, we again use the Gamma distribution, but with parameters

$$\alpha = b_L + b_R + 1\,, \;\; \beta = 2\rho h + 1 \tag{18}$$

to arrive at the formulation for the posterior density, namely

$$p(t \mid \rho, h, b_L, b_R) \;=\; \frac{(2\rho h+1)^{b_L+b_R+1}}{\Gamma(b_L+b_R+1)}\; t^{b_L+b_R}\, e^{-t(2\rho h+1)}\,. \tag{19}$$

Variable recombination rates. To consider recombination rate variation in our model, we can use the information provided by a high-resolution recombination map. Let ϱ denote the population-scaled genetic length of the focal shared haplotype segment, such that $\varrho = \rho h$ if the recombination rate is constant over the region. The genetic length of a shared haplotype segment is taken (estimated) from the recombination map as the genetic distance between the physical positions of its breakpoints, located at sites i and j, for which we use $f_{map}(i,j)$ as a function to return the genetic length in units of *Morgan* (M). Note that map units are usually specified in *centiMorgan* (cM), where $1\text{cM} = 0.01\text{M}$. We now can calculate $\varrho = 4N_e \times f_{map}(i,j)$, such that the rate of the Gamma distribution is $\beta = 2\varrho + 1$.

3.3 Joint clock model

We construct a joint model that considers both mutation and recombination. The same notation is used and parameters are modeled given the assumptions (and adjustments) as described for the mutation clock (Section 3.1) and the recombination clock (Section 3.2). From there, we may immediately arrive at the joint likelihood function in support of the coalescence time t as the product of the two likelihoods given in Equations (6) and (16);

$$\mathcal{L}(t \mid \theta, \rho, h, s, b_L, b_R) \;\propto\; t^{s+b_L+b_R}\, e^{-th(\theta+2\rho)}\,. \tag{20}$$

However, it is convenient to replace the term $h(\theta + 2\rho)$ with $(\vartheta + 2\varrho)$, where ϑ involves the (variable) mutation rate as described on Page 7 and ϱ involves the (variable) recombination rate as described on Page 10. We therefore write

$$\mathcal{L}(t \mid \vartheta, \varrho, s, b_L, b_R) \;\propto\; t^{s+b_L+b_R}\, e^{-t(\vartheta+2\varrho)} \tag{21}$$

from which we obtain the posterior probability as

$$\begin{aligned} p(t \mid \vartheta, \varrho, s, b_L, b_R) \;&\propto\; \mathcal{L}(t \mid \vartheta, \varrho, s, b_L, b_R) \;\times\; \pi(t) \\ &\propto\; t^{s+b_L+b_R}\; e^{-t(\vartheta+2\varrho+1)} \;. \end{aligned} \tag{22}$$

Conveniently, both the mutation and recombination clock models specify the Gamma distribution for the calculation of the posterior density. We therefore have

$$\begin{aligned} \alpha \;&=\; s + b_L + b_R + 1 \;, \\ \beta \;&=\; h(\theta + 2\rho) + 1 \;=\; \vartheta + 2\varrho + 1 \end{aligned} \tag{23}$$

to calculate the posterior density as

$$p(t \mid \vartheta, \varrho, s, b_L, b_R) \;=\; \frac{(\vartheta + 2\varrho + 1)^{s+b_L+b_R+1}}{\Gamma(s + b_L + b_R + 1)} t^{s+b_L+b_R} e^{-t(\vartheta+2\varrho+1)} \;. \tag{24}$$

4 Composite posterior estimation of variant age

Our approach to estimate allele age is similar to existing composite likelihood methods that are applied to solve problems where the full likelihood function is unknown or intractable. Here, coalescence between a haplotype pair (concordant or discordant) is seen as a lower-dimensional feature of the local genealogical structure of the sample at a given focal variant. We combine information from hundreds or thousands of pairs to obtain an estimate of the time the allele has emerged through mutation.

The posterior density of the time to coalescence for a given pair is defined for the clock models presented above (Section 3). Each clock model calculates the posterior using the Gamma distribution with parameters α and β. For simplicity, we express the posterior density using $p(t \mid \lambda)$, where we use $\lambda = \{\alpha, \beta\}$ to connote the parameters determined from haplotype data under a specific model. The probability of coalescence more recently than (or at) time t is obtained from the cumulative distribution function (CDF) of the posterior density;

$$\overline{\Lambda}(t \mid \lambda) \;=\; P(T \leq t \mid \lambda) \;=\; \int_0^t p(u \mid \lambda)\, du \;. \tag{25}$$

The probability of coalescence subsequent to time t (further back in time) is likewise

$$\begin{aligned} \underline{\Lambda}(t \mid \lambda) \;=\; P(T > t \mid \lambda) \;&=\; \int_t^\infty p(u \mid \lambda)\, du \\ &=\; 1 - \overline{\Lambda}(t \mid \lambda) \;. \end{aligned} \tag{26}$$

We use the notation $\{i, j\} \leftarrow \mathcal{C}_k$ to indicate that a pair was sampled (without replacement) from the set of possible concordant pairs and, likewise, $\{i, j\} \leftarrow \mathcal{D}_k$ for discordant pairs, where i, j indicate the two haplotypes in a pair. Sampling from either set is done as described

in Section 2, but where we additionally remove concordant and discordant pairs that appear to be inconsistent, for which we employ a heuristic algorithm (described in Section 5).

The age of an allele observed at target site k is estimated from the composite posterior distribution;

$$\Phi_k^\tau(t) \;\propto \prod_{\{a,b\}\leftarrow\mathcal{C}_k} \overline{\Lambda}\big(t \mid \lambda_k^\tau(a,b)\big) \;\times \prod_{\{c,d\}\leftarrow\mathcal{D}_k} \underline{\Lambda}\big(t \mid \lambda_k^\tau(c,d)\big) \tag{27}$$

where τ indicates the coalescent clock model used. The composite posterior can now be obtained over $t \in (0, \infty)$, where t is scaled in units of $2N_e$.

We record the mode as a point estimate of variant age, but also report the mean and median of the composite distribution, as well as a 95% confidence interval (non-parametric calculation based on the CDF of the composite posterior). In practice, when hundreds or thousands of pairwise TMRCA posteriors are combined (multiplied), the resulting composite posterior is expected to be over-confident; for example, we expect the mean and median of the distribution to converge on the mode as the number of concordant and discordant pairs increases.

Any estimate of variant age is implicitly bounded by the properties of the TMRCA posteriors from which the composite posterior is formed, which depend on mutational and recombinational information observed at the haplotype segments shared between the pairs considered. For very recent relationships, the TMRCA estimate will tend towards zero as the length, h, of the shared haplotype segment increases; the expected value of TMRCA follows from the Gamma distribution, given by $\mathrm{E}[T] = \alpha/\beta$, where only β involves h (in all clock models), such that $\lim_{h\to\infty} \mathrm{E}[T] = 0$ irrespective of the values of N_e or the prior. Conversely, we expect h to decrease as the TMRCA increases, as recombination had more time to break down the length of the shared segment. The oldest relationships discernible from sequence data may therefore be found at segments with a length of a few basepairs, where the recombination breakpoints occur between immediately neighboring variable sites. In such cases, due to the lack of information, the TMRCA estimate strongly depends on the values of N_e and the prior. However, because the composite posterior is formed by combining the cumulative distributions of TMRCA, coalescent events that occurred more distant in time, relative to the age of the focal allele, may have only little influence on the shape of the composite posterior. Nevertheless, the values of N_e and the prior determine the theoretical upper bound for estimates of the TMRCA and, thus, estimates of the age of the oldest alleles.

5 Heuristic method to reject outlier pairs

There are several sources of error that may adversely affect the estimation of allele age when using the composite posterior approach described above. For example, a given focal variant may have been falsely called or missed, the allele may have been lost due to back-mutation, or some of the shared haplotype segments may have been inferred incorrectly. To reduce the

impact of such outliers on the estimation process, we perform quality control on the set of pairs before they are included in the computation of the composite posterior distribution.

Using a simple, heuristic algorithm, a time threshold is ascertained above and below which concordant and discordant pairs are rejected, respectively; see schematic below. The number of available concordant pairs is given by $n_\mathcal{C}$, where $1 \leq n_\mathcal{C} \leq \max_\mathcal{C}$, and the number of discordant pairs is given by $n_\mathcal{D}$, where $1 \leq n_\mathcal{D} \leq \max_\mathcal{D}$. The mean of the inferred coalescence time distribution is taken as a point estimate for the TMRCA of each pair, calculated as $\mathrm{E}[T] = \alpha/\beta$, where α, β are determined from haplotype data per pair as defined for a given clock model. The sets of concordant and discordant pairs are sorted, separately, from lowest to highest mean TMRCA. For simplicity, we have

$$\bar{c}_1,\ \bar{c}_2,\ \bar{c}_3,\ \ldots,\ \bar{c}_{n_\mathcal{C}} \ \text{ and }\ \bar{d}_1,\ \bar{d}_2,\ \bar{d}_3,\ \ldots,\ \bar{d}_{n_\mathcal{D}}$$

where $\bar{c}_i$ and $\bar{d}_i$ connote the mean TMRCA of a given concordant and discordant pair, respectively. We find a threshold to reject pairs if $\bar{d}_1 < \bar{c}_{n_\mathcal{C}}$, or we reject none otherwise. The threshold is placed such that the minimum total number of pairs is rejected. However, we keep the most recent concordant pair and the oldest discordant pair, so as to ensure that there is at least one pair in each group.

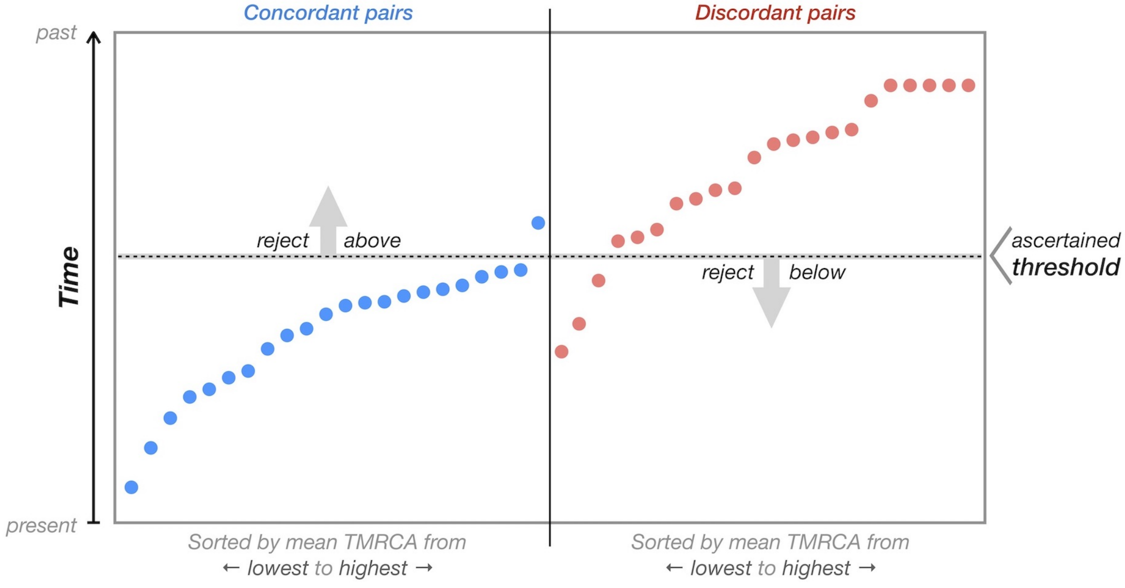

Schematic of concordant and discordant pair filtering. Available concordant (*left*) and discordant pairs (*right*) are independently sorted by mean TMRCA. A subset of pairs are rejected if distributions overlap. We use a heuristic algorithm to ascertain a threshold above and below which concordant and discordant pairs are rejected, respectively. The threshold is determined such that the minimum total number of pairs is rejected.

5.1 Quality score

The proportion of concordant and discordant pairs rejected for a variant may provide a simple metric to evaluate the quality of its age estimate (resulting from a given clock model).

We calculate a quality score, QS, as

$$QS = 1 - \max\left\{\frac{r_{\mathcal{C}}}{n_{\mathcal{C}}}, \frac{r_{\mathcal{D}}}{n_{\mathcal{D}}}\right\} \tag{28}$$

where $r_{\mathcal{C}}$ is the number of rejected concordant pairs and $r_{\mathcal{D}}$ the number of rejected discordant pairs. Values near or equal 1 indicate high quality, and values near 0 indicate low quality; note that at least one pair is retained in each group, such that $0 < QS \leq 1$.

References

1. Kimura M. The number of heterozygous nucleotide sites maintained in a finite population due to steady flux of mutations. Genetics. 1969;61(4):893–903.

2. Kimura M. Theoretical foundation of population genetics at the molecular level. Theoretical population biology. 1971;2(2):174–208.

3. Hein J, Schierup M, Wiuf C. Gene genealogies, variation and evolution: a primer in coalescent theory. New York: Oxford University Press; 2005.
