# Supplementary material for: Dating genomic variants and shared ancestry in population-scale sequencing data
Source: PLoS Biol. 2020 Jan 17;18(1):e3000586. doi: 10.1371/journal.pbio.3000586 (PMC6992231; doi:10.1371/journal.pbio.3000586)
Supplement: S2 Text — (PDF) [file pbio.3000586.s015.pdf]

S2 Text

Dating of genomic variants and shared ancestry in population-scale sequencing data

Patrick K. Albers[1*] and Gil McVean[1]

[1]Big Data Institute, Li Ka Shing Centre for Health Information and Discovery, University of Oxford, United Kingdom

*patrick.albers@bdi.ox.ac.uk

Contents

1 Assessment of genotype error in genomic data 2
1.1 Data preparation 2
1.2 Genotype error profile 3
2 Generation of simulated data 6
2.1 Integration of error in simulated data 8
2.2 Additional haplotype error through *in silico* phasing 8
3 Shared haplotype estimation using a simple hidden Markov model (HMM) 9
3.1 Transition model 10
3.2 Empirical emission model 12
3.3 Empirical initial state model 15
4 Simulation study 17
4.1 Estimation of variant age in simulated sample data 17
4.2 Variant age estimation based on PSMC 19
4.3 Performance of the heuristic pair rejection method 20

Analysis of data error and application in simulations

We describe the construction of an empirical genotype error profile in Section 1. Information derived from the error profile was used to

- reproduce realistic rates of error in simulated data (described in Section 2),
- develop a method for detecting shared haplotype segments while being robust towards data error when applied to real genomic data sets (Section 3), and
- evaluate the performance of GEVA (which includes the method for shared haplotype detection) and characterise the effects on age estimation in simulations of large sample data before and after the inclusion of error (Section 4).

1 Assessment of genotype error in genomic data

Any assessment of error in empirical data requires the existence of an error-free "gold standard" against which data can be compared; provided that data were obtained on the same biological sample. We used genotype data from the Illumina Platinum Genomes (IPG) project as a reference "truthset" [1],* against which we assessed error in matched data from the 1000 Genomes Project (TGP) [2];† for human assembly GRCh37 (hg19), which are available for both panels.

In the following, we use g to denote the assumed true genotypic state (as seen in IPG), and $\tilde{g}$ to denote the genotype that has been observed in the assessed data set (TGP), at a given locus and for the same individual. We define the coefficient $e_{i \to j}$ to denote the rate at which a true genotype g_i was observed as genotype $\tilde{g}_j$, where the subscripts $i, j \in \{0, 1, 2\}$ indicate the genotypic state; homozygous for the reference allele (0), heterozygous (1), or homozygous for the alternative allele (2).

1.1 Data preparation

The TPG sample comprises 2,504 individuals sequenced (or genotyped) at >80 million SNPs, for which data has been generated using a combination of low-coverage whole-genome sequencing ($> 4\times$), high-coverage exome sequencing ($> 50\times$), and microarray genotyping [2]. Data from IPG comprises 4.7 million SNPs generated using high-coverage whole-genome sequencing of a 17-member, three-generation family of European ancestry (`CEPH 1463`); namely, four individuals in the founder generation, two in the parental generation, and eleven children. The IPG sample has been sequenced at $50\times$ coverage on Illumina HiSeq 2000 and variants have been called in accordance with different methods to resolve conflicts among different call sets. The two parents (IDs `NA12877` and `NA12878`) have been additionally sequenced at $200\times$ coverage and variant data has been validated based on Mendelian inheritance constraints from pedigree information [1].

Cell lines from `CEPH 1463` are a well-characterized model system and have been sequenced or genotyped in several studies. Data for individual `NA12878` was available in both the IPG and TGP panels. We compared genotype data that we extracted from both panels for this individual, where the genotypic state observed in IPG was assumed to be the "true" state. Although the possibility that IPG retained misclassified genotypes at a certain fraction of variant sites cannot be excluded, we assumed this fraction to be negligibly small. Likewise, the impact of other sources of error, such as somatic mutations that occurred in the sampled biological material and thereby may produce different results when processed on different platforms, were assumed to be negligible.

We extracted genotype data for all autosomes and matched sites by chromosome position between the two panels. We only considered biallelic SNPs, and we excluded sites at which the

* https://emea.illumina.com/platinumgenomes.html
† ftp://ftp.1000genomes.ebi.ac.uk//vol1/ftp/release/20130502/

reference or alternate alleles were inconsistent between IPG and TGP. Data were additionally filtered using the accessibility mask available from IPG, thereby retaining only sites called with high confidence in IPG. Conversely, however, we did not filter sites based on high-confidence regions available for the TGP panel, due to the underlying intention to measure error as typically encountered in analyses of genomic data. The matching processes is illustrated in the figure below.

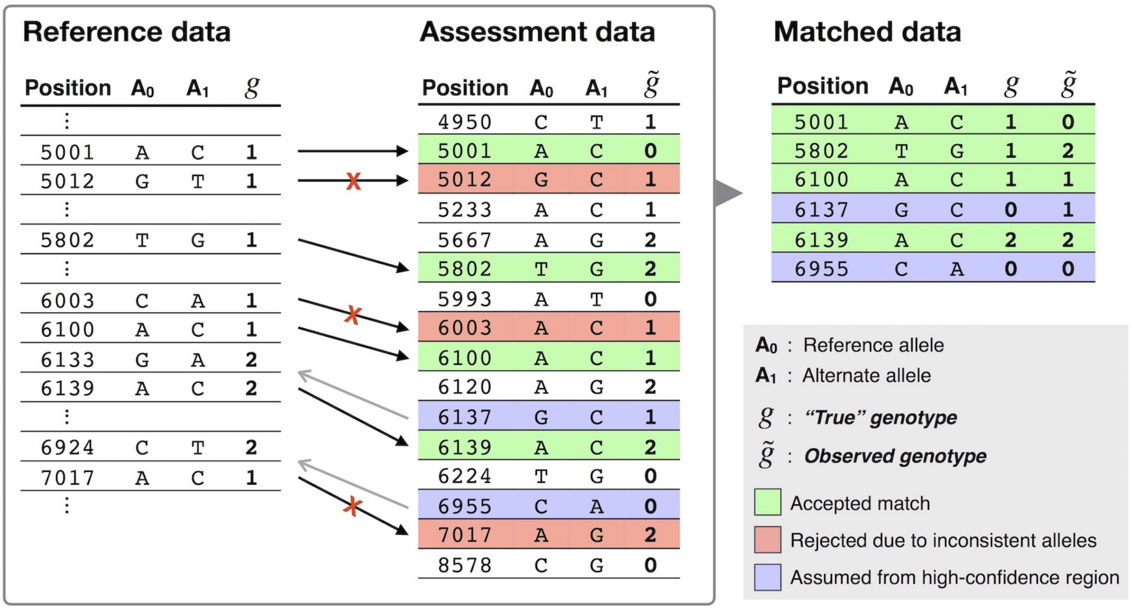

Schematic of the genotype matching process. Genotype information of individual NA12878 were extracted from the Illumina Platinum Genomes (IPG) truthset and data from the 1000 Genomes Project (TGP) Phase 3, from all autosomes, and sites were matched by chromosome position (GRCh37). We used the accessibility mask available for the IPG panel to retain variant sites called with high-confidence (indicated by gaps in the figure). Sites were removed if reference or alternate alleles did not match between IPG and TGP. Because the reference truthset (IPG) did not contain genotypes homozygous for the reference allele, these were assumed from high-confidence regions if present in the assessment dataset (TPG); indicated by left-pointing arrows. Figure modified from [3].

Note that available IPG data did not contain variants called as being homozygous for the reference allele (g_0). This is because the high-confidence regions in IPG have been identified in the individual call sets by collating sites that were called as being homozygous for the reference allele and monomorphic in the sample [1]. Variants homozygous for the alternate allele (g_2) have not been removed. Here, we made the following assumption to be able to measure error proportions pertaining to each genotypic state. The true state was assumed to be of the g_0 type if the position of a variant site in TGP was within high-confidence regions of the IPG accessibility mask. We assumed that high-confidence intervals encompassed variants which would have been reported as a different type otherwise.

1.2 Genotype error profile

We measured genotype error at 76 million comparisons between IPG and TGP. Of those, 73.2 million were homozygous for the reference allele. While the vast majority of genotypes

(>99%) have been called (or typed) without error, we found 0.08% of genotypes to be misclassified. The confusion matrix given in the table (below) shows the overall proportion of states observed per true genotypic state. We found that the density of erroneous genotypes increased towards the telomeric and centromeric regions on each chromosome (see panel A in the figure shown on Page 5), where we see error densities of >0.2% on all chromosomes, but >1% on most chromosomes, on a genome-wide background of ~0.1% on average.

Relative error rates measured per genotype class. Genotype data from individual NA12878 was available at 76 million comparisons between the Illumina Platinum Genomes (IPG) truthset and the 1000 Genomes Project (TGP) across autosomes. Error rates were calculated as the relative proportion a given true genotype g_i was observed as $\tilde{g}_j$, where $i, j \in \{0, 1, 2\}$; thus, summing to 100% per column. The total number of genotypes assessed per true genotype class is given below.

Observed genotype	True genotype		
	g_0	g_1	g_2
$\tilde{g}_0$	99.942%	0.550%	0.034%
$\tilde{g}_1$	0.041%	99.282%	0.231%
$\tilde{g}_2$	0.017%	0.168%	99.735%
Total	73,211,532	2,076,098	1,326,955

Next, we constructed a frequency-dependent genotype error profile. Assessed sites were assigned their population frequency as observed in the TGP sample and then pooled into 200 evenly distributed frequency bins. We calculated

$$e_{i \to j} = P(g_i \to \tilde{g}_j | f), \text{ for } i, j \in \{0, 1, 2\} \tag{1}$$

as the relative rate a true genotype g_i was observed as $\tilde{g}_j$ for sites at allele frequency f (recorded at the mean per bin), and normalized to sum to 1 per true genotype class; $\sum_{j=0}^{2} e_{i \to j} = 1$. These results are shown in panel B of the figure on Page 5.

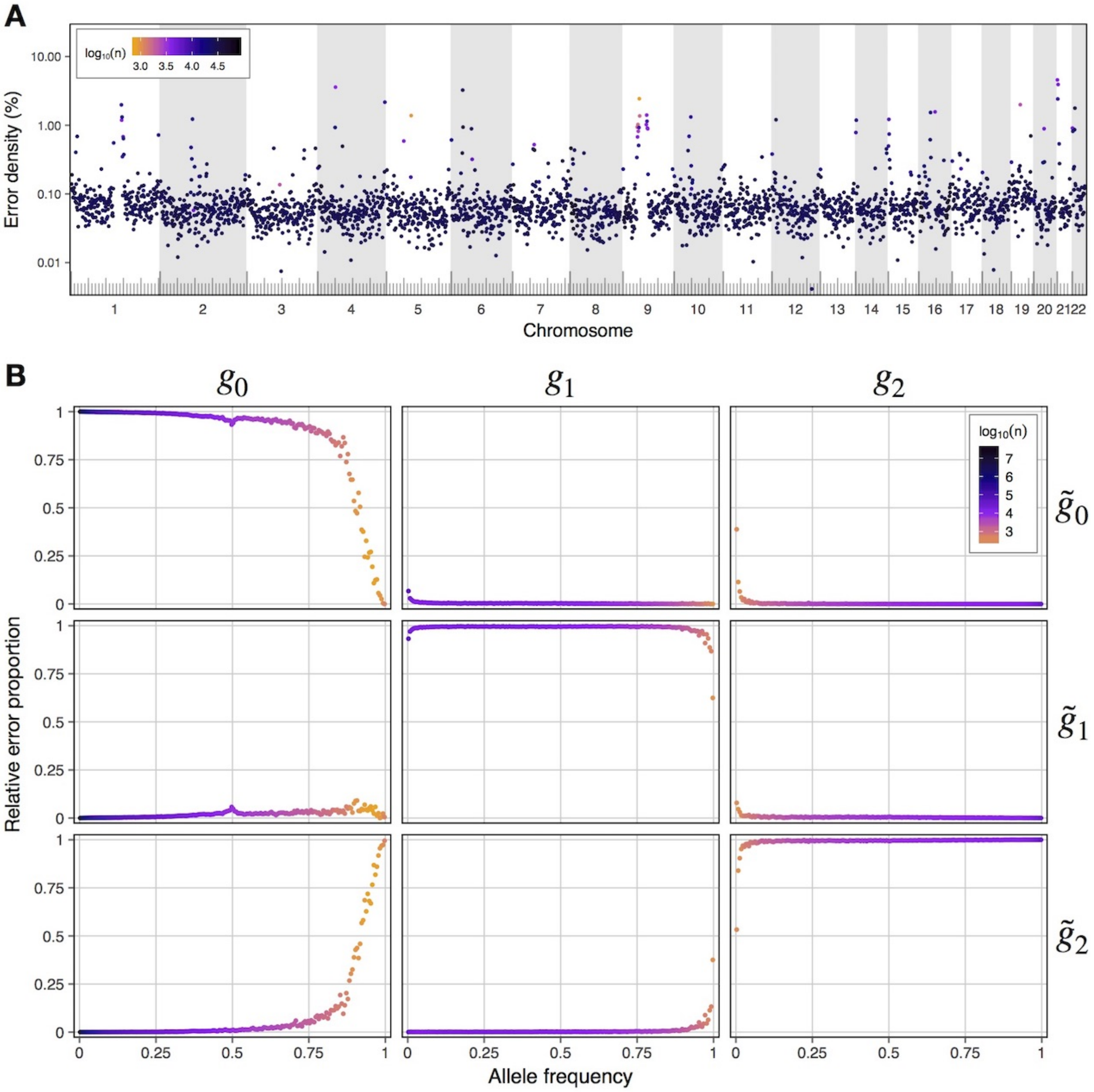

Genotype error measured in data from the 1000 Genomes Project. Genotype error was measured at 76 million comparisons between the Illumina Platinum Genomes (IPG) truthset and the 1000 Genomes Project (TGP), on genotype data extracted for the same individual (`NA12878`) from all autosomes. Panel **A** shows measured error densities by region on Chromosomes 1-22. The density of misclassified genotypes ($g \neq \tilde{g}$) was calculated in equally sized chunks of 1 Mb size along the length of each chromosome. Error density was calculated as the number of misclassified genotypes divided by the total number of genotypes per chunk; percentage shown on log-scale. Colors indicate the number of genotypes per chunk (see legend). The ruler at the bottom indicates the physical length per chromosome, where longer tick marks sit 10 Mb apart. Panel **B** shows the confusion matrix of relative error measured per genotypic class, given the allele frequency at matched sites in the TGP sample. The set of matched genotypes was pooled into 200 equally sized allele frequency bins. Relative error, $e_{i \to j}$, was calculated by counting g_i observed as $\tilde{g}_j$ and dividing by the sum of g_i per frequency bin; for $i, j \in \{0, 1, 2\}$. Colors indicate the number of genotypes per bin (see legend).

2 Generation of simulated data

We performed coalescent simulations using `msprime` software [4]. The software stores the history of the simulated sample, which can be queried in downstream analyses through its `python` interface. We used data generated from two main simulations (described below); in the following referred to as data sets $\mathcal{A}$ and $\mathcal{B}$. We further modified haplotype data in a copy of data set $\mathcal{B}$ to include empirically estimated rates of error (Section 2.1); in the following referred to as data set $\mathcal{B}'$. A copy of this data set was further modified by performing *in silico* haplotype phasing (Section 2.2); in the following referred to as data set $\mathcal{B}''$. The process of generating data sets $\mathcal{B}$, $\mathcal{B}'$, and $\mathcal{B}''$ is summarized in the figure shown below.

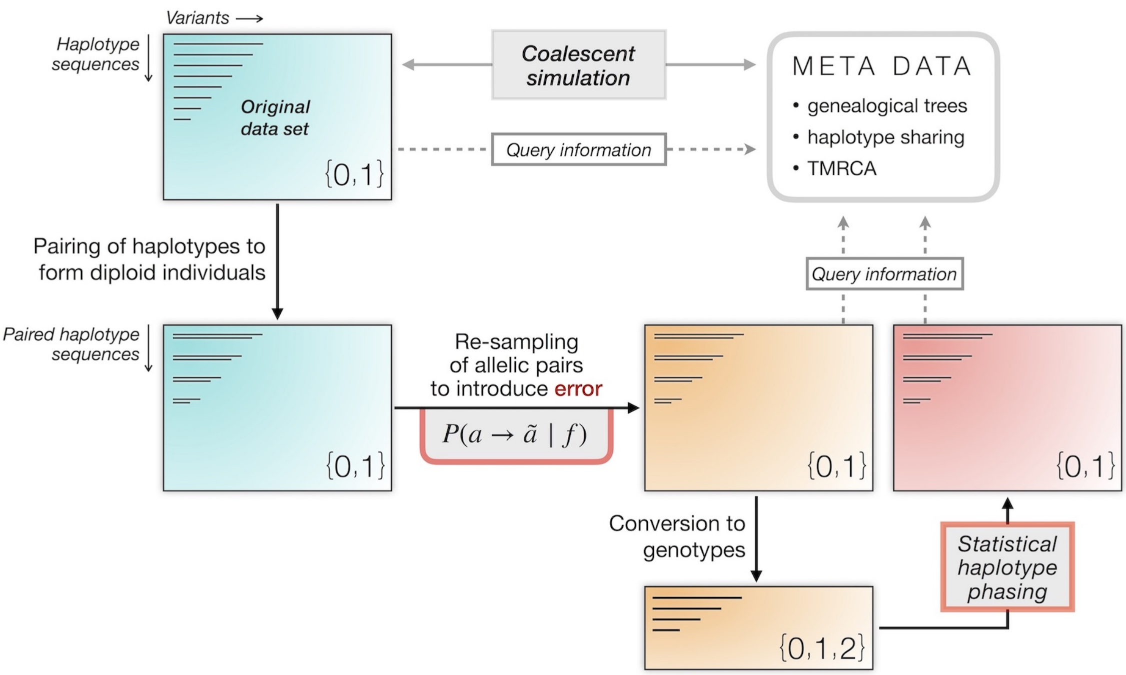

Schematic of the data generation process. The figure summarizes the generation of data sets $\mathcal{B}$ (*blue*), $\mathcal{B}'$ (*orange*), and $\mathcal{B}''$ (*red*). We performed coalescent simulations using `msprime` software [4], which produced a sample of haplotype sequences and a corresponding record (*meta data*) of the genealogical history of the simulated sample. Haplotype data was stored in *variant call format* (VCF), in which haploid sequences were arranged in pairs to form diploid individuals. We used this sample configuration to introduce error by scanning along the paired sequence in each individual, but where we used the frequency-dependent genotype error profile constructed in Section 1 to introduce error on haplotype-level. A given allelic pair, a, was re-sampled as $\tilde{a}$ with probability $P(a \to \tilde{a}|f)$, where f is the sample allele frequency at the current site; see Equation (2). We further modified the resulting haplotype data set by performing *in silico* haplotype phasing using `SHAPEIT2` [5] after conversion to genotype data. Because these data were derived from the same original data set, its simulation record can be queried in downstream analyses of each generated data set. However, note that genealogical information may not be retrieved conclusively in data after haplotype phasing.

Simple demographic model simulation ($\mathcal{A}$). We simulated a sample of N=1,000 haplotypes of 100 Mb length, with N_e=10,000 and constant and equal mutation and recombination rates ($\mu = 1 \times 10^{-8}$, $r = 1 \times 10^{-8}$, per base per generation). The full command to simulate this data set is given in Script 1 (below).

Script 1. Simple demographic model; single demographic unit without migration, constant population size, and constant and equal mutation and recombination rates.

```
import msprime
data = msprime.simulate(Ne = 10000,
                        sample_size = 1000,
                        mutation_rate = 1e-08,
                        recombination_rate = 1e-08,
                        length = 100000000)
data.dump("history.hdf5") # simulation record
with open("sample.vcf", "w") as vcf_file:
        data.write_vcf(vcf_file, ploidy = 2) # output haplotype data in VCF file
```

Complex demographic model simulation ($\mathcal{B}$). We simulated a sample of $N = 5,000$ haplotypes ($N_e = 7,300$; $\mu = 2.35 \times 10^{-8}$ per base per generation) under a demographic model that recapitulates the human expansion out of Africa [6], with population growth and migration between three major populations (African, Asian, European). The simulation was conducted with variable rates of recombination, for which we used the genetic map for Chromosome 20 from HapMap (Phase 2; GRCh37) [7], which also determined the length of the simulated region (∼63 Mb). The full command to simulate this data set is given in Script 2 (below).

Script 2. Complex demographic model; out-of-Africa model following [6], with population growth and migration between three major populations (African, Asian, European), with constant mutation rate, and variable recombination rates in Chromosome 20 from HapMap (Phase 2; GRCh37) [7]. Script modified from the `msprime` manual (https://msprime.readthedocs.io).

```
import msprime
import math
rec_map = msprime.RecombinationMap.read_hapmap("genetic_map_GRCh37_chr20.txt")
mut_rate = 2.35e-8
N_A = 7300
N_B = 2100
N_AF = 12300
N_EU0 = 1000
N_AS0 = 510
generation_time = 25
T_AF = 220e3 / generation_time
T_B = 140e3 / generation_time
T_EU_AS = 21.2e3 / generation_time
r_EU = 0.004
r_AS = 0.0055
N_EU = N_EU0 / math.exp(-r_EU * T_EU_AS)
N_AS = N_AS0 / math.exp(-r_AS * T_EU_AS)
m_AF_B = 25e-5
m_AF_EU = 3e-5
m_AF_AS = 1.9e-5
m_EU_AS = 9.6e-5
population_configurations = [
    msprime.PopulationConfiguration(sample_size=0, initial_size=N_AF),
    msprime.PopulationConfiguration(sample_size=5000, initial_size=N_EU, growth_rate=r_EU),
    msprime.PopulationConfiguration(sample_size=0, initial_size=N_AS, growth_rate=r_AS)
]
migration_matrix = [
    [      0, m_AF_EU, m_AF_AS],
    [m_AF_EU,       0, m_EU_AS],
    [m_AF_AS, m_EU_AS,       0],
]
demographic_events = [
    msprime.MassMigration(time=T_EU_AS, source=2, destination=1, proportion=1.0),
    msprime.MigrationRateChange(time=T_EU_AS, rate=0),
    msprime.MigrationRateChange(time=T_EU_AS, rate=m_AF_B, matrix_index=(0, 1)),
    msprime.MigrationRateChange(time=T_EU_AS, rate=m_AF_B, matrix_index=(1, 0)),
    msprime.PopulationParametersChange(time=T_EU_AS, initial_size=N_B, growth_rate=0, population_id=1),
    msprime.MassMigration(time=T_B, source=1, destination=0, proportion=1.0),
    msprime.PopulationParametersChange(time=T_AF, initial_size=N_A, population_id=0)
]
data = msprime.simulate(Ne = N_A,
                        mutation_rate = mut_rate,
                        recombination_map = rec_map
                        population_configurations = population_configurations,
                        migration_matrix = migration_matrix,
                        demographic_events = demographic_events)
data.dump("history.hdf5") # simulation record
with open("sample.vcf", "w") as vcf_file:
        data.write_vcf(vcf_file, ploidy = 2) # output haplotype data in VCF file
```

2.1 Integration of error in simulated data

The frequency-dependent genotype error profile constructed in Section 1 was used to modify a copy of simulated data set $\mathcal{B}$, but where we introduced error on haplotype-level. This modified version is referred to as data set $\mathcal{B}'$. We first sorted haplotypes into pairs to form diploid individuals composed of two allelic sequences. Note that `msprime` already provides the option to output simulated haploid sequences in *variant call format* (VCF) for diploids. Alleles are encoded as 0s and 1s for the ancestral and derived allelic states, respectively. There are four possible ordered pairs of alleles; namely

$$a_{00} = (0,0),\ a_{01} = (0,1),\ a_{10} = (1,0),\ a_{11} = (1,1).$$

We scanned along the paired sequence of each individual, where, for a given allelic pair a, we sampled $\tilde{a}$ from the set of possible pairs $\{a_{ij}\}_{i,j \in \{0,1\}}$, with probability according to the empirically determined rate of genotype error; given by

$$P(a \to \tilde{a} | f) = \begin{array}{c} \begin{matrix} \tilde{a}_{00} & \tilde{a}_{01} & \tilde{a}_{10} & \tilde{a}_{11} \end{matrix} \\ \begin{bmatrix} e_{0\to0} & \frac{e_{0\to1}}{2} & \frac{e_{0\to1}}{2} & e_{0\to2} \\ e_{1\to0} & e_{1\to1} & 0 & e_{1\to2} \\ e_{1\to0} & 0 & e_{1\to1} & e_{1\to2} \\ e_{2\to0} & \frac{e_{2\to1}}{2} & \frac{e_{2\to1}}{2} & e_{2\to2} \end{bmatrix} \begin{matrix} a_{00} \\ a_{01} \\ a_{10} \\ a_{11} \end{matrix} \end{array} \tag{2}$$

where f is the allele frequency in the simulated sample at the current site along the sequence. Recall that the coefficient $e_{i\to j}$ captures the relative rate of error per genotypic class at a set of recorded allele frequencies; see Equation (1). At sites where the observed frequency did not match to frequencies recorded in the error profile, we used linear interpolation to approximate error rates, which we again normalized to sum to 1 per true genotype class (corresponding to rows in Equation 2).

2.2 Additional haplotype error through *in silico* phasing

We used a copy of data set $\mathcal{B}'$ (described above) to introduce additional errors through *in silico* haplotype phasing. Note that haplotype sequences were already sorted to form diploid individuals in the generated VCF output, and we maintained this configuration after the integration of data error. We computed the genotype sequence per individual as the sum of alleles at each position. This converted data set was then used to statistically re-estimate haplotypes without a reference panel using `SHAPEIT2` [5]. The haplotype phasing process was expected to introduce single-site ("flip") and long-range phase ("switch") errors.

3 Shared haplotype estimation using a simple hidden Markov model (HMM)

We have developed a hidden Markov model (HMM) to locally infer the region a given pair of chromosomes (concordant or discordant) share by descent from their MRCA at a focal position. Relative to a given target site in the genome, the shared haplotype segment is delimited by ancestral recombination events that occurred independently to the left and right-hand side in either of the two lineages considered. Our HMM is constructed as a two-state model in which the *local* genealogy at a given target site is distinguished from any *peripheral* genealogies that generated the variation seen outside the local segment. The two states are denoted by H_0 (local) and H_1 (peripheral). We scan the sequence from the position of a given target site until the end of the chromosome, which we do in two independent runs for the sequence to the left and to the right-hand side. The Viterbi algorithm is then used to decode the hidden state sequence from which we find a recombination breakpoint at the first occurrence of the H_1 state. If the H_0 state was inferred at all sites until the end of the chromosome, we record the breakpoint to sit beyond the last position of the sequence. The breakpoints inferred on both sides identify the sequence interval which encloses the local shared haplotype segment. This is illustrated in the figure shown below. In cases where the H_1 state was inferred throughout (including the focal site at the initial position), we exclude the current (concordant or discordant) haplotype pair from downstream analyses.

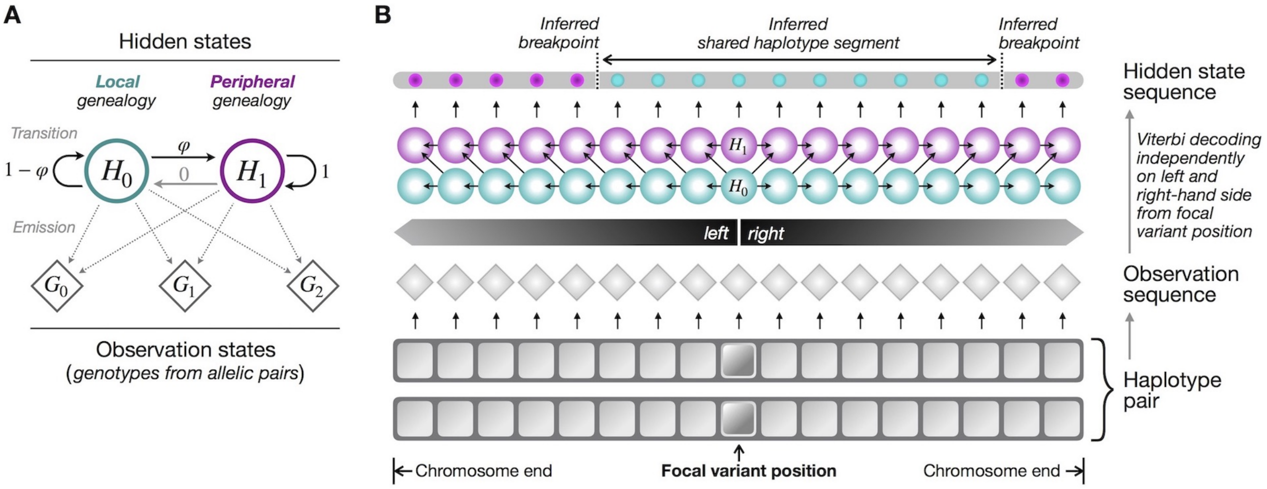

Schematic of the hidden Markov model (HMM). Panel **A** gives a graphic representation of the hidden state transitions and observation state emissions of the HMM. We define two hidden states to discriminate the *local* genealogy (H_0) which generated the variation observed at and around a given target site from any *peripheral* genealogies (H_1) outside the focal segment. The observation sequence is encoded as genotypes (G_0, G_1, G_2) by combining alleles (encoded as 0s and 1s) along the sequence of the haplotype pair considered. The probability of transition from the local to the peripheral state is denoted by φ. The HMM is constructed using a *left-to-right* architecture in which transitions from a higher to a lower-numbered state have zero probability. Emission probabilities were determined using an empirically generated model with realistic rates of data error. Panel **B** shows the HMM trellis, illustrating (*from bottom to top*) how two haplotype sequences are paired to form the observation sequence. Starting at the position of a given focal variant, the HMM is applied independently to the sequence to the left and right-hand side, to infer the nearest recombination *breakpoints* that delimit the enclosed shared haplotype segment.

The observation sequence is constructed from SNP variant data from the two sequences considered. Alleles are encoded as 0s and 1s, denoting the reference and alternate allele, respectively. We assume that the alternate allelic state has been correctly assigned to the derived allele. The two sequences are paired such that each site is represented as an unsorted pair of alleles (genotypes) with three possible observation states; namely

$$G_0 = \{0, 0\},\ G_1 = \{0, 1\},\ G_2 = \{1, 1\}.$$

Note that we do not consider a separate state for missing alleles, but rather exclude sites from the observation sequence at which one or both alleles have been missed.

3.1 Transition model

Our HMM employs a *left-to-right* architecture, meaning that the state transitions proceed in one direction where transitions from a higher to a lower-numbered state have zero probability. We define the transition matrix

$$A \ = \begin{bmatrix} a_{00} & a_{01} \\ a_{10} & a_{11} \end{bmatrix} \ = \ \begin{bmatrix} 1-\varphi & \varphi \\ 0 & 1 \end{bmatrix} \tag{3}$$

where the coefficient a_{ij} denotes the probability of transition from state H_i to state H_j. Since we have $a_{10} = 0$ (and thus $a_{11} = 1$), it is implied that we cannot return to the local genealogy once it has been left. The transition from the local to the peripheral state is given by $a_{01} = \varphi$, which is dependent on the genetic distance between consecutive variants in the sequence and the number of meioses (generations) separating the two haplotypes considered.

From the target site at position k, the HMM proceeds until the end of the chromosome to either the left or right-hand side relative to k. At the current site l in the sequence, we define δ_l as the genetic distance observed between l and the immediately previous site at position $l-1$. The time since the two chromosomes inherited the local haplotype from a common ancestor is treated as an unknown, but we use the following approximation to make broad distinctions of coalescence times based on the sample frequency f_k of the allele observed at target site k. For concordant pairs, we calculate [8]

$$\xi_k \ = \ \frac{-2f_k}{1-f_k}\ \log(f_k)\ , \quad 0 < f_k < 1 \tag{4}$$

which is the expected age of a selectively neutral allele at frequency f_k in a population of constant size. Since the above is dependent on the frequency of the allele observed at the target site, it does not apply to discordant pairs, for which we set $\xi_k = 1$, as we cannot derive an expectation when the allele is not shared by both haplotypes. We use the above to obtain an approximation for the probability of transition from the local to a peripheral genealogy as

$$\varphi_l(k) \ = \ 1 - e^{-4N_e \delta_l \xi_k} \tag{5}$$

where N_e is the diploid effective size of the population. Note that we therefore compute a transition matrix $A_l(k)$ at every site along the sequence, dependent on the allele frequency at the target site k and the genetic distance at the current site l.

Transition probabilities computed for different focal allele frequencies are illustrated in panel A of the figure shown below. We note that differences in transition probabilities resulting from focal allele frequency variation, in practice, have little impact on the allele age estimation process. This is demonstrated through analysis of simulated data set $\mathcal{A}$ (see Section 2). First, we calculated transition probabilities conditional on focal allele frequency, as defined in this section and which is the default in GEVA. We then analyzed the same data, but set $\xi_k = 1$ for all haplotype pairs to make the calculation of transition probabilities independent of frequency. We found that age estimates were highly correlated (Spearman rank correlation, $\rho > 0.98$) for each clock model; see panel B in the figure below.

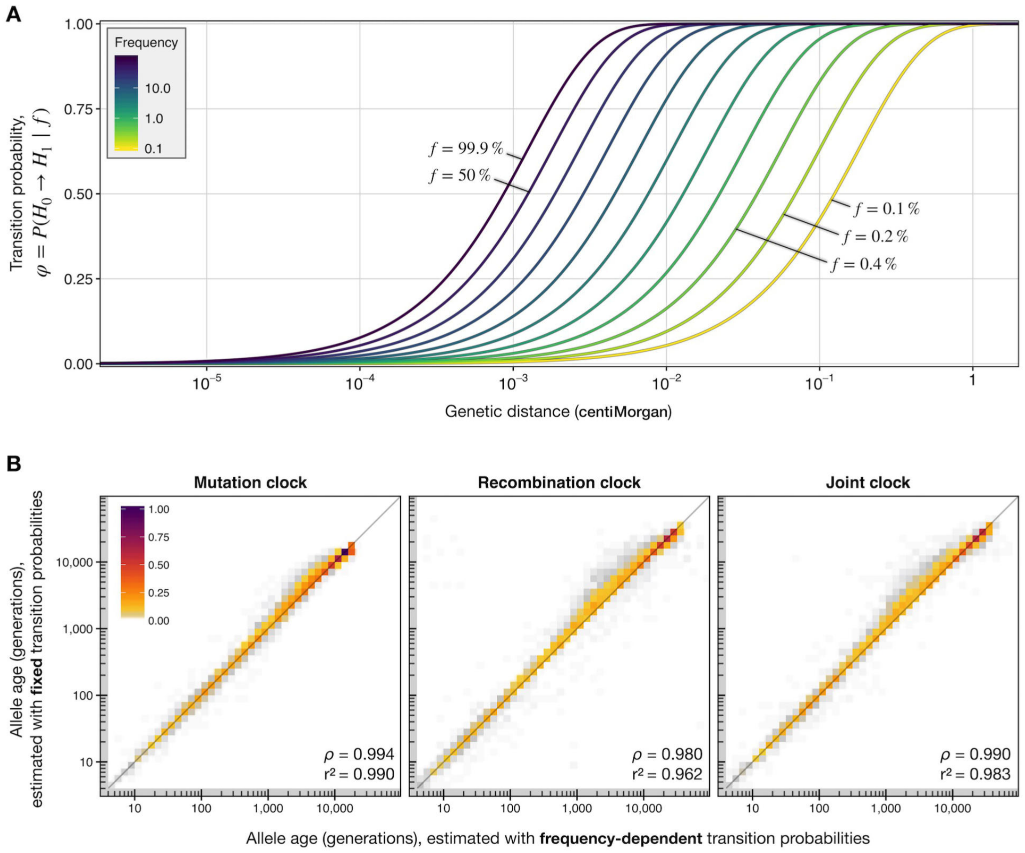

Transition probabilities. Panel **A** shows the probability of transition from the local (H_0) to the peripheral (H_1) state, φ, given the genetic distance (*x-axis*) for different allele frequencies (see legend; labels indicate certain low and high frequencies). Note that the genetic distance is given in *centiMorgan* in the figure, but transition probabilities are computed in units of *Morgan* in Equation (5). In the HMM, genetic distance is measured between consecutive sites along the sequence, and allele frequency is taken as the observed frequency of the alternative allele in the sample at a given focal site. Panel **B** shows density scatterplots of allele age estimated ($\max_{\mathcal{C}} = 500$; $\max_{\mathcal{D}} = 500$) under each clock model for 5,000 variants (randomly drawn at allele count $1 < x < N$) from data simulated with sample size N=1,000 haplotypes, N_e=10,000, $\mu = 1 \times 10^{-8}$, and $r = 1 \times 10^{-8}$; see Section 2 (Script 1). The same set of variants was analyzed using GEVA, but with frequency-dependent transition probabilities in the HMM (*x-axis*), and with fixed transition probabilities (*y-axis*). For the latter, we set $\xi_k = 1$ for all haplotype pairs; see Equation (5). Lower inserts indicate the Spearman rank correlation statistic, ρ, and the squared Pearson correlation coefficient (on log-scale), r^2, calculated between the two corresponding sets of age estimates.

3.2 Empirical emission model

We generated an empirical model from simulated data, which we modified to include realistic distributions of error, to make the HMM robust in applications to real data. The model is defined by a set of observation probabilities (emissions), denoted by $\sigma_j^i(f_l)$, where i identifies the hidden state $\{H_i\}_{i\in\{0,1\}}$ and j the genotypic state $\{G_j\}_{j\in\{0,1,2\}}$ observed at site l along the sequence of the two haplotypes considered. Emissions are obtained conditional on the allele frequency, f_l, observed in the sample at site l. Note that the emissions for the two hidden states (H_0, H_1) are based on TMRCA, but which involves a simplifying assumption (described further below) to construct a time-independent, approximate emission model.

The model was constructed by estimating the relative rates of observing each genotypic state in pairwise shared haplotype segments (identified from genealogical records after simulation using `msprime`; see Section 2), given allele frequency information and the TMRCA for each segment. This was done through an iterative sampling process. We randomly selected two haplotypes from simulated data and identified the locations of recombination breakpoints to detect shared haplotype segments (non-recombinant sequence intervals). Specifically, we scanned each genealogical tree along the sequence and recorded breakpoints at coordinates where the genealogical relationship changed due to recombination, such that the sequence interval in between two consecutive breakpoints was derived from the same MRCA. Note that the continuous position coordinates internally stored by `msprime` may differ from the discrete genomic positions after output to variant call format (VCF). We matched breakpoint coordinates to the returned genomic positions to identify sequence intervals that are observable from the data. For each segment, we recorded the TMRCA and retrieved the enclosed variant sequences from both haplotypes. The genotypic states observed at each site in the sequence interval were recorded together with the sample allele frequency at each site. Segments shorter than 10 variant sites were removed. We repeated this process until collecting $\sim$100 million segments (from >30,000 random haplotype pairs).

To illustrate differences arising from data error, we first analyzed data set $\mathcal{B}$, which was simulated using `msprime` under a complex demographic model that recapitulates the human expansion out of Africa (described in Section 2). This is compared to data set $\mathcal{B}'$, which is a copy of $\mathcal{B}$ but where haplotypes were modified using empirically estimated error rates (described in Section 2.1). Both data sets thereby have the same genealogical history.

We pooled segments into 100 TMRCA bins (evenly distributed on log-scale between 1 generation and 500,000 generations), within which we pooled genotypes into 500 allele frequency bins (evenly distributed on linear scale). For each combination of TMRCA and frequency bin, the relative rate of observing each genotypic state was obtained by normalizing counts (to sum to 1). The resulting time and frequency-dependent distributions are shown in panel A of the figure shown on Page 13. We found that the main differences were located at the extremes of either TMRCA or frequency. But notably, before error, the relative rate of observing heterozygous genotypes (G_1) was zero (or near zero) in each frequency bin for recent TMRCA (<10 generations), but non-zero throughout after error.

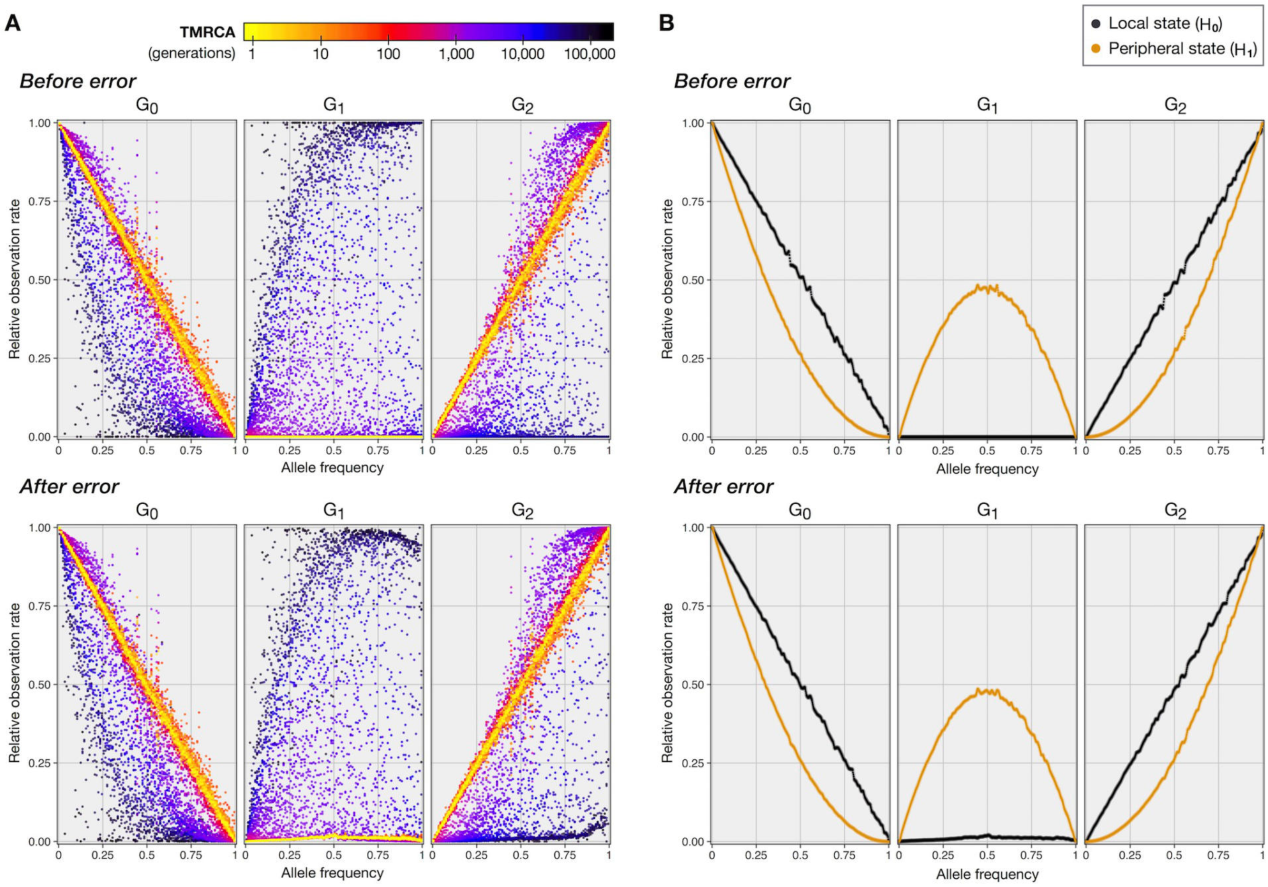

Empirical emission probabilities. The relative rate of observing each genotypic state (G_0, G_1, G_2) in simulated data before error (data set $\mathcal{B}$; *top*) and after error (data set $\mathcal{B}'$; *bottom*). Panel **A** shows the relative rates measured in 100 TMRCA bins, evenly distributed on log-scale between 1 generation and 500,000 generations (see legend), and for variants pooled into 500 evenly distributed allele frequency bins. For each combination of TMRCA and frequency bin, measured rates were normalized to sum to 1 across the three genotypic states. Panel **B** shows the relative rates as in (A), but where a nominal distinction at 100 generations was made to determine emission probabilities in the local state (H_0; $\leq$ 100 generations) and the peripheral state (H_1; $>$ 100 generations) by allele frequency.

Next, we estimated emissions as defined for the model, specifically $\sigma_j^i(f_l)$, for which we pooled segments into only two TMRCA bins, to make an approximate distinction between recent shared ancestry and older genealogical relationships. We applied a nominal cutoff at 100 generations to distinguish segments in hidden state H_0 (TMRCA $\leq$ 100 generations) from those in hidden state H_1 (TMRCA $>$ 100 generations). The resulting observation rates (after pooling into 500 allele frequency bins) are shown in panel B of the figure shown above, for the analyses before and after error. We used the observation rates obtained from data after error as the emission model in subsequent applications of GEVA. These results are available as part of the online repository of the GEVA software.* For frequencies not captured by the set of recorded frequency bins, in practice (within GEVA), we use linear interpolation to approximate the emissions at the observed sample allele frequency, which we again normalize to ensure that observation rates sum to 1 per hidden state.

* https://github.com/pkalbers/geva/blob/master/hmm/hmm_emission_probs.txt

Note that the distinction of TMRCA to determine observation rates in H_0 and H_1 is arbitrary for any nominal cutoff. We see this as a useful simplification to distinguish a *local* shared haplotype segment from the background "noise" of variation produced through *peripheral* genealogies. However, one caveat is that we implicitly assume that local TMRCA is on average younger than TMRCA at neighboring segments. Inference of breakpoints is expected to be less problematic at older segments if the local segment sits within an extended region where variation is derived from similarly old relationships, because we would quickly transition into the peripheral state and infer a breakpoint nearby. But, conversely, it is less likely that we can accurately infer breakpoints that delimit an older and short local segment if the immediately following segment is younger and relatively long.

Further, we note that a nominal cutoff at 100 generations, in practice, may not reflect a limit on the ability to infer breakpoints at shared haplotype segments with higher or lower TMRCA, or on the performance of the method to estimate allele age. This is demonstrated through comparison of age estimation in GEVA, performing pairwise analyses of the HMM with emission models generated from different nominal cutoffs of TMRCA. We used the default emission model generated with a TMRCA cutoff at 100 generations (from data after error) and prepared a second model in the same way, but with a cutoff at 1,000 generations. For the same set of 5,000 randomly selected variants in data set $\mathcal{B}'$, we found that age estimates were highly correlated (Spearman rank correlation, $\rho > 0.94$) for each clock model; see figure below.

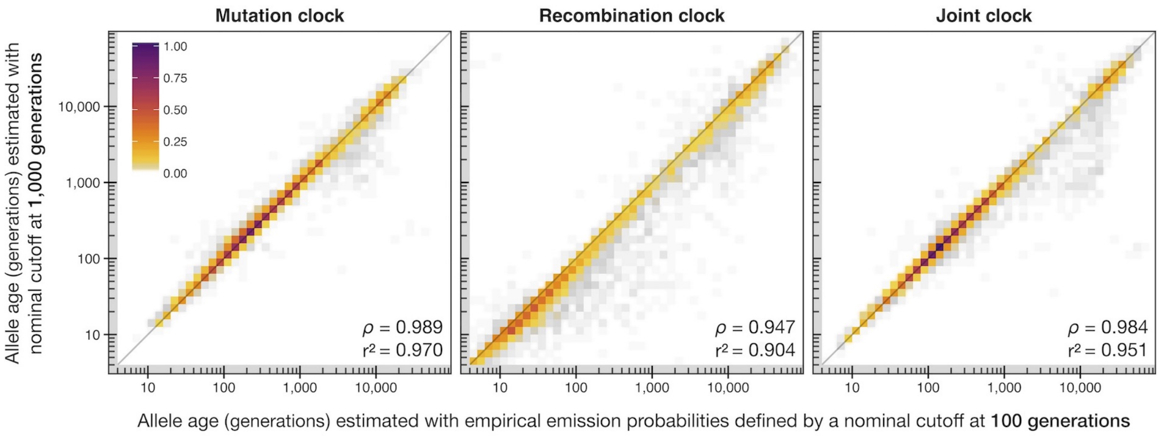

Comparison of allele age estimated with different empirically generated emission models. The correlation between allele age estimated using GEVA with two different emission models in the HMM. Emissions were generated from simulated data (after including realistic distributions of error), but with different nominal cutoffs to distinguish the two hidden states (H_0 and H_1) based on younger and older TMRCA, respectively. This was done by applying a TMRCA cutoff at 100 generations (*x-axis*) and with a cutoff at 1,000 generations (*y-axis*). Each panel shows the density scatterplots of allele age estimated ($\max_{\mathcal{C}} = 500$; $\max_{\mathcal{D}} = 500$) under a given clock model, for the same set of 5,000 variants (randomly drawn at allele count $1 < x < N$) from data set $\mathcal{B}'$, simulated with sample size N=5,000 haplotypes, N_e=7,300, $\mu = 2.35 \times 10^{-8}$, and variable recombination rates (HapMap2, Chromosome 20); see Section 2 (Script 2). Lower inserts indicate the Spearman rank correlation statistic, ρ, and the squared Pearson correlation coefficient (on log-scale), r^2, calculated between the two corresponding sets of age estimates.

3.3 Empirical initial state model

Similar to the generation of emission probabilities described above, we estimated the initial probability of being in either H_0 or H_1 empirically from simulated data. For concordant pairs, we define $\pi_i^{\mathcal{C}}(f_k)$ as the initial probability of being in state $\{H_i\}_{i \in \{0,1\}}$, where f_k is the sample allele frequency at the focal site k. Likewise, we define $\pi_i^{\mathcal{D}}(f_k)$ for discordant pairs. Again, we used simulation data sets $\mathcal{B}$ and $\mathcal{B}'$ (described in Section 2 and Section 2.1, respectively) to obtain frequency-dependent estimates for $\pi_i^{\mathcal{C}}$ and $\pi_i^{\mathcal{D}}$ from the true positive rate (TPR) of correctly observing allelic combinations by comparing pairs ($\{1, 1\}$ for concordant pairs, $\{0, 1\}$ or $\{1, 0\}$ for discordant pairs) before and after error.

In data without error, we have $\pi_0^{\mathcal{C}} = 1$ and $\pi_0^{\mathcal{D}} = 1$ with certainty at any f_k (and therefore $\pi_1^{\mathcal{C}} = 0$ and $\pi_1^{\mathcal{D}} = 0$). To estimate initial state probabilities in data after error, we used data set $\mathcal{B}'$ to divide the set of variants by allele count (simulated sample size N=5,000). We then randomly sampled up to 1,000 sites per allele count category ($1 <$ allele count $< N$) and, for each site, sampled up to 1,000 pairs per set of possible concordant and discordant pairs. For both groups, we computed the TPR by comparison to the allelic combination seen at the same position and for the same pair in data before error (data set $\mathcal{B}$). We recorded the TPR at each site and report mean TPR per allele count category; see figure shown on Page 16.

We defined $\pi_0^{\mathcal{C}}(f_k)$ and $\pi_0^{\mathcal{D}}(f_k)$ according to the recorded mean TPR at a given allele frequency, and set $\pi_1^{\mathcal{C}}(f_k) = 1 - \pi_0^{\mathcal{C}}(f_k)$ and $\pi_1^{\mathcal{D}}(f_k) = 1 - \pi_0^{\mathcal{D}}(f_k)$. These results are available as part of the online repository of the GEVA software.* Because these were recorded at a fixed set of allele frequencies, we use linear interpolation to approximate the probabilities at sample allele frequency f_k, which we again normalize to ensure that probabilities sum to 1 per hidden state, separately for concordant and discordant pairs.

* https://github.com/pkalbers/geva/blob/master/hmm/hmm_initial_probs.txt

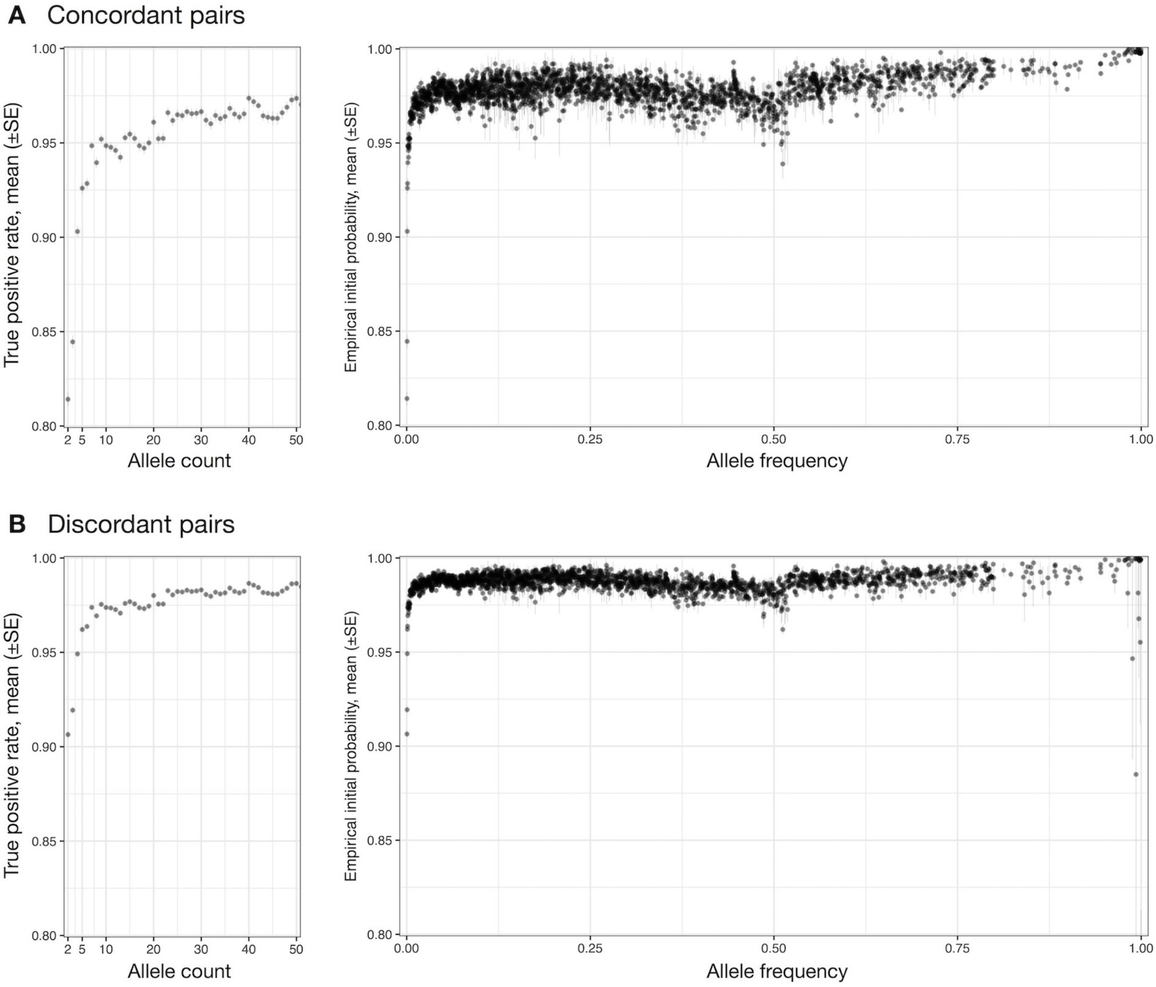

Empirical initial state probabilities. The probability of being in the local H_0 state at the initial position in the sequence was estimated from the true positive rate (TPR) of correctly observing the allelic combination $\{1, 1\}$ for concordant and $\{0, 1\}$ or $\{1, 0\}$ for discordant pairs, by comparing the same pairs in data before error (data set $\mathcal{B}$) to corresponding coordinates in data after error ($\mathcal{B}'$). The TPR was measured at sets of up to 1,000 variants randomly selected per allele count category ($1 <$ allele count $< N$; where N=5,000 is the number of haplotypes in the simulated sample), for which up to 1,000 concordant or discordant pairs were sampled per variant. Panel **A** shows the mean TPR (±SE) per allele count category measured for concordant pairs; shown in greater detail for allele counts in $[2, 50]$ (*left*), and across the full allele frequency range (*right*). Panel **B** shows the same as **A**, but for discordant pairs.

4 Simulation study

We used simulated data to assess the genealogical estimation of variant age (GEVA) method (Section 4.1), and compared GEVA to a strategy that employs PSMC as a method to infer TMRCA posteriors from which allele age can be estimated (Section 4.2). Also, we evaluated the performance of the heuristic rejection method (described in **S1 Text**) through which outliers in the inferred TMRCA distribution are excluded prior to age estimation in GEVA (Section 4.3).

4.1 Estimation of variant age in simulated sample data

The performance of GEVA was evaluated in terms of its accuracy to infer the TMRCA between pairs of haplotypes, and to estimate allele age from the inferred TMRCA distributions. We used simulation data sets $\mathcal{A}$ and $\mathcal{B}$, which we generated under different demographic models (described in Section 2). To reproduce the conditions typically present in applications to real data, we also applied GEVA to data sets $\mathcal{B}'$ and $\mathcal{B}''$, which we derived from $\mathcal{B}$, but where haplotype data were modified to contain realistic proportions of data error ($\mathcal{B}'$; described in Section 2.1) and additional error through *in silico* haplotype phasing ($\mathcal{B}''$; described in Section 2.2).

We applied GEVA with scaling parameters as specified for each simulation. For data set $\mathcal{A}$, we used N_e =10,000, $\mu = 1 \times 10^{-8}$, and $r = 1 \times 10^{-8}$. Allele age was estimated for 5,000 variants randomly drawn from sites with allele count $1 < x < N$, which we analyzed with $\max_{\mathcal{C}} = 500$ and $\max_{\mathcal{D}} = 500$ as the maximum number of concordant and discordant pairs sampled per site (given a sample size of N =1,000). Thus, we inferred TMRCA posteriors from locally estimated shared haplotype segments at >3 million concordant and discordant pairs. We used our heuristic rejection method to exclude outlier pairs before estimating age.

Similarly, for $\mathcal{B}$ and its derived data sets $\mathcal{B}'$ and $\mathcal{B}''$, we used N_e =7,300, $\mu = 2.35 \times 10^{-8}$, and the HapMap genetic map (Phase 2; GRCh37; Chromosome 20) to determine variable recombination rates over the simulated region. Data were simulated with a sample size of N =5,000. Allele age was estimated for the same set of variants in $\mathcal{B}$, $\mathcal{B}'$, and $\mathcal{B}''$, with $\max_{\mathcal{C}} = 500$ and $\max_{\mathcal{D}} = 500$. We selected 5,000 variants at random from the intersection of sites at which the observed allele count satisfied $1 < x < N$ across the three data sets. More than 3 million haplotype pairs were analyzed in each data set, where we excluded outliers before estimating age.

The results of the analysis of data set $\mathcal{A}$ are shown in **S1 Fig**, in which we used different metrics to measure estimation bias and correlation between true and estimated allele age (**S1 Fig**A) and pairwise TMRCAs inferred at each site (**S1 Fig**B), for each of the three clock models. Likewise, the results for data set $\mathcal{B}$ are shown in **S2 Fig**, data set $\mathcal{B}'$ in **S3 Fig**, and data set $\mathcal{B}''$ in **S4 Fig**. The metrics are described below.

Evaluation of allele age. Let t denote the true time of the mutation event that gave rise to the allele at a given site in the sample, for which we obtain an estimated age denoted by $\hat{t}$. Coalescent simulators typically do not record the actual point in time when a (neutral) mutation occurred, as the probability of mutation is proportional to the branch length in a simulated genealogical tree, and mutations are placed uniformly over a given branch. To obtain a "true" value for allele age, we queried the simulation meta-data as recorded by `msprime` to locate the branch on which a specific mutation occurred. A branch is delimited by the time all carrier haplotypes have coalesced into a single lineage and the time this lineage joined with any of the remaining lineages. Let t^0 and t^1 denote the times of coalescent events that occurred immediately before and after the mutation event, respectively, as for the simulated sample, where $t^0 \leq t$ and $t^1 > t$. We retrieved t^0 and t^1 from simulation records to calculate the true time of a mutation as $t' = \sqrt{t^0 \times t^1}$, which is the geometric mean. Note that the arithmetic mean would be appropriate given that mutation events are placed uniformly between t^0 and t^1 in the neutral coalescent. However, we found the geometric mean to be more reliable when analyzing larger sets of variants that exhibit high variability in terms of mutational timing and branch lengths.

Correlation between true (t) and estimated ($\hat{t}$) allele age was measured using Spearman's rank correlation coefficient, ρ. Additionally, we calculated Pearson's r^2 on log-scaled values of true and estimated allele age. Estimation error was measured using the root mean squared $\log_{10}$ error (RMSLE), calculated as

$$\text{RMSLE} = \sqrt{\frac{1}{n}\sum_{i=1}^{n}\Big(\log_{10}[\,t_i\,] - \log_{10}[\,\hat{t}_i\,]\Big)^2} \tag{6}$$

where n is the number of variants considered.

We compute the following metric as a measure for estimation bias, denoted by ϵ, which is adjusted for the time interval between t^0 and t^1. Error is quantified as the mean difference relative to the time interval during which a focal mutation occurred, where underestimation of allele age is calculated relative to t^0, and overestimation relative to t^1; calculated as

$$\epsilon = \frac{1}{n}\sum_{i=1}^{n} I_i^0\left(\frac{t_i^0 - \hat{t}_i}{t_i^0}\right) + I_i^1\left(\frac{\hat{t}_i - t_i^1}{t_i^1}\right) \tag{7}$$

where I^0 and I^1 are indicator functions given by

$$I_i^0 = \begin{cases} 0 & \text{if } \; t_i^0 < \hat{t}_i \\ 1 & \text{otherwise} \end{cases}, \; I_i^1 = \begin{cases} 0 & \text{if } \; t_i^1 > \hat{t}_i \\ 1 & \text{otherwise} \end{cases}. \tag{8}$$

Implicitly, this considers any age estimate falling in between t^0 and t^1 as being correct, thereby making ϵ robust towards variations in branch lengths when considering larger sets of variants with different coalescent histories.

Evaluation of pairwise TMRCA. We used Spearman's ρ and Pearson's r^2 to measure correlation between true and inferred TMRCA for the sets of concordant and discordant pairs sampled for each variant, as well as RMSLE as an error metric. The true time of coalescence was determined from simulation records, and we used the mean of the Gamma distribution as a point estimate of the inferred TMRCA, calculated as $\mathrm{E}[T] = \alpha/\beta$, where the values of α, β are obtained through analysis under a given clock model (see **S1 Text**).

4.2 Variant age estimation based on PSMC

The *Pairwise Sequentially Markovian Coalescent* (PSMC) model has been used to infer historic changes in population size back in time [9], using sequence data from two haplotypes alone (or one diploid individual). The model is based on the *Sequentially Markov Coalescent* (SMC) model for analytically tractable approximation to the ancestral recombination graph (ARG) in model-based inferences [10, 11]. Here, we employed the HMM-based PSMC method [9] for TMRCA inference between two chromosomal sequences. In this approach, time is divided into a number of discrete intervals, which are the hidden states of the HMM. Using the forward-backward algorithm, a posterior probability is obtained for each state at different sites equally spread across the full length of the chromosome.

Implementation. Here we used an implementation of the PSMC-based HMM to infer the posterior distribution of coalescence times for concordant and discordant pairs at a given target position; thus effectively treating PSMC as a substitute clock model. The `decode` algorithm implemented in software available for the *Multiple Sequentially Markovian Coalescent* (MSMC) method [12] specifically applies the PSMC-HMM when two haplotype sequences are provided as input data. We modified `decode` to only output posterior probabilities at a specified target position, but without hindering the computation of posteriors along the sequence. The modified version of `decode` is available online.*

Pairwise TMRCA inference. We first used GEVA to estimate allele age for the sets of variants selected from each simulated data set ($\mathcal{A}, \mathcal{B}, \mathcal{B}', \mathcal{B}''$), to then apply the modified `decode` algorithm on haplotype data for the same sets of concordant and discordant pairs as sampled through GEVA (before excluding outlier pairs). The number of discrete time intervals (hidden states) was set to 64, which is the default in `decode`. For simulation panel $\mathcal{A}$, we used N_e=10,000, $\mu = 1 \times 10^{-8}$, and $r = 1 \times 10^{-8}$ to specify the scaled mutation and recombination rates. For $\mathcal{B}$, $\mathcal{B}'$, and $\mathcal{B}''$, we used N_e =7,300 and $\mu = 2.35 \times 10^{-8}$ to specify the scaled mutation rate ($\theta = 2N_e\mu$). Because `decode` does not operate on variable recombination rates, we fixed the scaled recombination rate parameter to 80% of the value of θ, which is the recommended setting.† A total of >14 million haplotype pairs was analyzed across all four simulated data sets.

* https://github.com/pkalbers/msmc2
† https://github.com/stschiff/msmc/blob/master/guide.md

Allele age estimation. We first applied our heuristic method to reject outlier pairs in the sets of concordant and discordant pairs that were analyzed per focal variant. For this, we took the mode of the inferred posterior distribution to obtain a point estimate of the TMRCA per pair, which we recorded at the mean between time interval boundaries. We then estimated allele age on the posteriors of the retained pairs using a composite posterior approach similar to the one used by GEVA. Specifically, we computed

$$F(t) = \sum_{i=1}^{t} p(i) \times \max\left[\sum_{j=1}^{n} p(j)\right]^{-1}, \text{ for } t = 1, 2, \ldots, n \tag{9}$$

for concordant pairs and $F'(t_i) = 1 - F(t_i)$ for discordant pairs to approximate the cumulative posterior distribution, where n is the number of discrete time intervals and $p(i)$ the posterior probability inferred at the ith time interval. The PSMC-derived composite posterior distribution was then computed as

$$\Phi(t) \propto \prod_{\{a,b\} \in C} F_{a,b}(t) \times \prod_{\{c,d\} \in D} F'_{c,d}(t), \text{ for } t = 1, 2, \ldots, n \tag{10}$$

where C and D refer to the pre-selected (and subsequently filtered) sets of available concordant and discordant pairs, respectively. A point estimate of allele age was taken at the mode of the composite posterior distribution which we recorded at the mean between time interval boundaries.

4.3 Performance of the heuristic pair rejection method

We evaluated our heuristic rejection method (see **S1 Text**) in terms of the proportion of haplotype pairs rejected in data before and after error, for each clock model and the PSMC-based approach. In data before error ($\mathcal{B}$), pairwise TMRCA inference under the joint clock model led to a rejection of 2.4% of pairs, across the 5,000 variants analyzed, which was similar for the mutation clock (2.3%) and higher for the recombination clock (3.4%). In data after error ($\mathcal{B}'$), 8.1% of pairs were rejected for the joint clock, 7.6% for the mutation clock, and 10.7% for the recombination clock. Additional phasing of data after error ($\mathcal{B}''$) showed no notable differences compared to the proportion of pairs rejected in data set $\mathcal{B}'$. When using PSMC to infer TMRCA, only 1.7% of pairs were rejected in the analysis on data before error ($\mathcal{B}$), but 8.9% in data after error ($\mathcal{B}'$ or $\mathcal{B}''$).

We further evaluated our heuristic filtering approach in terms of its accuracy to reject pairs that have been selected by GEVA due to data error. This was done by comparing the pairs selected in the analysis of data set $\mathcal{B}'$ to their true allelic configuration in haplotype data from $\mathcal{B}$; results are given in the table on Page 21. For the 5,000 variants analyzed, we measured the true positive rate (TPR) of correctly retaining error-free pairs, which was >94% for concordant or discordant pairs and under each clock model. The true negative rate (TNR) of correctly rejecting erroneous pairs was overall lower and differed between the two groups;

>58% for concordant pairs and >38% for discordant pairs under each clock model. Filtering of pairs inferred using the PSMC-based approach resulted in a similar TPR overall (>94% for concordant or discordant pairs), but the TNR was much lower compared to either clock model (9% for concordant pairs and 12.2% for discordant pairs), such that PSMC (within the implementation used here) showed the lowest accuracy.

Accuracy of rejecting haplotype pairs in data after error. The performance of our heuristic rejection method was evaluated in terms of its accuracy to reject erroneous haplotype pairs that have been selected by GEVA from data after error ($\mathcal{B}'$). Selected concordant and discordant pairs were distinguished into true and false positives by scanning their allelic configurations in data before error ($\mathcal{B}$). We report the true positive rate (TPR, or sensitivity) of correctly retaining error-free pairs, the true negative rate (TNR, or specificity) of correctly rejecting erroneous pairs, as well as the accuracy (ACC) as the sum of true positives and true negatives divided by the sum of all pairs; separately for the sets of concordant and discordant pairs selected across 5,000 variants analyzed under each clock model and the PSMC-based approach. However, because the 5,000 variants analyzed here were selected from the intersection of sites at which the observed allele count satisfied $1 < x < N$ across data sets $\mathcal{B}$, $\mathcal{B}'$, and $\mathcal{B}''$, the accuracy reported is likely to be artificially inflated overall.

Clock model	Concordant pairs			Discordant pairs		
	TPR	TNR	ACC	TPR	TNR	ACC
Mutation clock	97.0%	58.9%	96.0%	97.3%	38.2%	87.2%
Recombination clock	94.1%	66.6%	93.3%	94.3%	41.6%	85.3%
Joint clock	97.3%	60.0%	96.3%	96.8%	39.7%	87.0%
PSMC-based approach	94.6%	9.0%	37.3%	94.5%	12.2%	49.0%

References

1. Eberle MA, Fritzilas E, Krusche P, Kallberg M, Moore BL, Bekritsky MA, et al. A reference data set of 5.4 million phased human variants validated by genetic inheritance from sequencing a three-generation 17-member pedigree. Genome Research. 2016;27(1):1–9.

2. Auton A, Abecasis GR, Altshuler DM, Durbin RM, Bentley DR, Chakravarti A, et al. A global reference for human genetic variation. Nature. 2015;526(7571):68–74.

3. Albers PK. Rare and low-frequency variants and predisposition to complex disease. University of Oxford; 2017.

4. Kelleher J, Etheridge AM, McVean G. Efficient Coalescent Simulation and Genealogical Analysis for Large Sample Sizes. PLoS Computational Biology. 2016;12(5):e1004842–22.

5. Delaneau O, Zagury JF, Marchini J. Improved whole-chromosome phasing for disease and population genetic studies. Nature methods. 2013;10(1):5–6.

6. Gutenkunst RN, Hernandez RD, Williamson SH, Bustamante CD. Inferring the Joint Demographic History of Multiple Populations from Multidimensional SNP Frequency Data. PLoS Genetics. 2009;5(10):e1000695–11.

7. International HapMap Consortium, et al. A second generation human haplotype map of over 3.1 million SNPs. Nature. 2007;449(7164):851.

8. Kimura M, Ota T. The age of a neutral mutant persisting in a finite population. Genetics. 1973;75(1):199–212.

9. Li H, Durbin R. Inference of human population history from individual whole-genome sequences. Nature. 2011;475(7357):493–U84.

10. McVean GAT, Cardin NJ. Approximating the coalescent with recombination. Philosophical Transactions of the Royal Society B: Biological Sciences. 2005;360(1459):1387–1393.

11. Marjoram P, Wall JD. Fast "coalescent" simulation. BMC Genetics. 2006;7:16.

12. Schiffels S, Durbin R. Inferring human population size and separation history from multiple genome sequences. Nature Publishing Group. 2014;46(8):919–925.
