# Supplementary material for: Dating genomic variants and shared ancestry in population-scale sequencing data
Source: PLoS Biol. 2020 Jan 17;18(1):e3000586. doi: 10.1371/journal.pbio.3000586 (PMC6992231; doi:10.1371/journal.pbio.3000586)
Supplement: S3 Text — (PDF) [file pbio.3000586.s016.pdf]

S3 Text

Dating of genomic variants and shared ancestry in population-scale sequencing data

Patrick K. Albers[1*] and Gil McVean[1]

[1]Big Data Institute, Li Ka Shing Centre for Health Information and Discovery, University of Oxford, United Kingdom

*patrick.albers@bdi.ox.ac.uk

Contents

1 Information about ancestral and derived allelic states 2
2 Variants dated in 1000 Genomes Project (TGP) data 2
2.1 Pathogenic variants 3
3 Variants dated in Simons Genome Diversity Project (SGDP) data 5
4 Combined age estimation of variants present in TGP and SGDP 6

Estimation of variant age in publicly available data sets

We used GEVA to estimate the age of all variants on Chromosomes 1-22 (biallelic SNPs, except singletons and variants at alternate allele frequency >99%) in data from two human genome resources that are available in the public domain:

- 1000 Genomes Project (TGP), Phase 3, final release [1];
- Simons Genetic Diversity Project (SGDP), fully public data set [2].

The two data sets are described in detail further below. In total, we estimated the age of 45,393,705 variants across all autosomes, for each clock model (mutation clock, recombination clock, and joint clock). This includes 43,232,520 variants dated in sample data from TGP, and 15,834,824 variants from SGDP. For the 13,673,639 variants identified in both TGP and SGDP, we additionally estimated the age after combining information from both data sources (described in Section 4). Overall, we analyzed 32,087,462,147 (>32 billion) haplotype pairs. A breakdown of variants dated per chromosome, as well as a summary of the haplotype pairs analyzed, is given in **S1 Table**.

We make these results available as a public resource, referred to as the Atlas of Variant Age for the human genome. All data, including the full age estimation profiles for each clock model and the results of every pairwise analysis, are available online:

`https://human.genome.dating`

Throughout, we applied GEVA using the following specifications. We set N_e=10,000 to internally scale time in units of $2N_e$, which adheres to the usually quoted value [3]. Though, we note that more recent estimates of N_e indicate a much lower effective size and high variability among different human populations [4, 5]. Results are reported in units of generations, after rescaling time given the specified value for N_e. We assumed a constant rate of mutation, set to $\mu = 1.2 \times 10^{-8}$ per base per generation, following recent estimates of the human mutation rate [6]. We used variable recombination rates according to HapMap genetic maps as available per chromosome (Phase 2; GRCh37) [7].

The maximum number of concordant and discordant haplotype pairs sampled per variant (specified by parameters $\max_{\mathcal{C}}$ and $\max_{\mathcal{D}}$) differed for the analyses conducted using TGP and SGDP data (see below). Throughout, variant age was estimated after applying the heuristic pair rejection method to exclude outliers in the pairwise TMRCA distributions of concordant and discordant pairs selected per variant, and we report the quality score (QS) for the age estimated under each clock model.

1 Information about ancestral and derived allelic states

The GEVA method (in its current implementation) assumes that ancestral and derived allelic states have been correctly assigned to the reference and alternate allele, respectively, as seen in a given data set. We acquired information as available for the human genome from Ensembl (human assembly GRCh37; release 92; version 20180221),* to determine the ancestral allele as predicted through multi-species alignments in the Ensembl *Enredo-Pecan-Ortheus* (EPO) pipeline.† We used this information to annotate available variant data, so as to (optionally) retain those variants in downstream analyses for which the ancestral allele was known and mapped to the reference allele. In TGP, we matched variant annotations based on chromosomal position, rsID, and consistent reference and alternate alleles. In SGDP, we did the same but omitted matching by rsID, as this information was absent in the available data set.

2 Variants dated in 1000 Genomes Project (TGP) data

The final release TGP sample consists of 2,504 individuals (5,008 genomes) sampled from 26 populations worldwide.‡ We set $\max_{\mathcal{C}} = 500$ and $\max_{\mathcal{D}} = 500$ as the sampling limits for concordant and discordant pairs, respectively. For the 43.2 million variants dated across Chromosomes 1-22, we analyzed a total of 8.5 billion concordant and 21.3 billion discordant pairs, which involved the inference of the locally shared haplotype segment and the TMRCA posterior distribution at each pair. We recorded an overall computation time of approximately

* ftp://ftp.ensembl.org//pub/release-92/variation/vcf/homo_sapiens/homo_sapiens.vcf.gz
† https://www.ensembl.org/info/genome/compara/multiple_genome_alignments.html
‡ ftp://ftp.1000genomes.ebi.ac.uk//vol1/ftp/release/20130502/

2,699,936 hours (308 CPU years); measured as the sum of the system time elapsed per CPU core (Intel Xeon Gold 6126, Skylake SP, 2.60GHz).

We report the mode, mean, and median of the composite posterior distribution as point estimates for variant age, as well as a 95% confidence interval. Correlation between estimators was high throughout (Pearson's $r^2 > 0.99$; Spearman's $\rho > 0.99$, based on all 43.2 million variants, in all comparisons between mode, mean, and median, under each clock model).

We computed the quality score (QS) after rejecting outlier haplotype pairs for every variant and under each clock model; see panel A in the figure shown on Page 4. Estimation quality was high overall; for example, median QS was 0.985 for the joint clock model (0.991 mutation clock; 0.820 recombination clock), and the proportion of variants with $QS > 0.95$ was 58.3% (joint clock; 61.0% mutation clock; 37.2% recombination clock). Because GEVA, by default, attempts to estimate the age of the alternate allele (assuming that it is derived and the reference allele is ancestral), we investigated differences in estimation quality arising from violations of this assumption, given the ancestral allelic states as predicted from the Ensembl EPO pipeline (Section 1); see panel B in figure on Page 4. We found that 88.2% of variants had the ancestral allele as the reference allele; among those, median QS was 0.993 for the joint clock model (0.996 mutation clock; 0.846 recombination clock). Among the 6.9% of variants where the ancestral allele matched the alternate allele, estimation quality was lower overall; median QS was 0.645 for the joint clock model (0.680 mutation clock; 0.568 recombination clock). The relative proportion of variants with $QS > 0.95$ (joint clock) was 62.0%, 16.4%, and 52.2% for sites where the ancestral allele matched the reference, alternate, or neither allele, respectively. These results suggest that the quality score is informative as a measure of estimation quality, where low QS values may indicate departures from baseline model assumptions in GEVA. However, we note that other sources of error exist, and that high QS values may not validate estimation results.

2.1 Pathogenic variants

We used information from the Ensembl Variant Effect Predictor (VEP; release 75) [8], available through TGP,* where functional consequences have been assigned to a subset of variants in the TGP sample (Phase 3; GRCh37). Variant effects have been predicted using PolyPhen-2 [9] and SIFT [10]. We selected all variants that had annotations from either PolyPhen-2 or SIFT and obtained their age from the Atlas of Variant Age. The set comprised 70,220 variants annotated by PolyPhen-2 and 67,539 variants annotated by SIFT, where 67,123 variants had annotations from both methods. The results shown in **S7 Fig** were generated on age estimates obtained from TGP data for the joint clock model, after excluding variants with low estimation quality ($QS \leq 0.5$) and where the reference allele did not match the ancestral allele.

* ftp://ftp.1000genomes.ebi.ac.uk//vol1/ftp/release/20130502/supporting/functional_annotation/

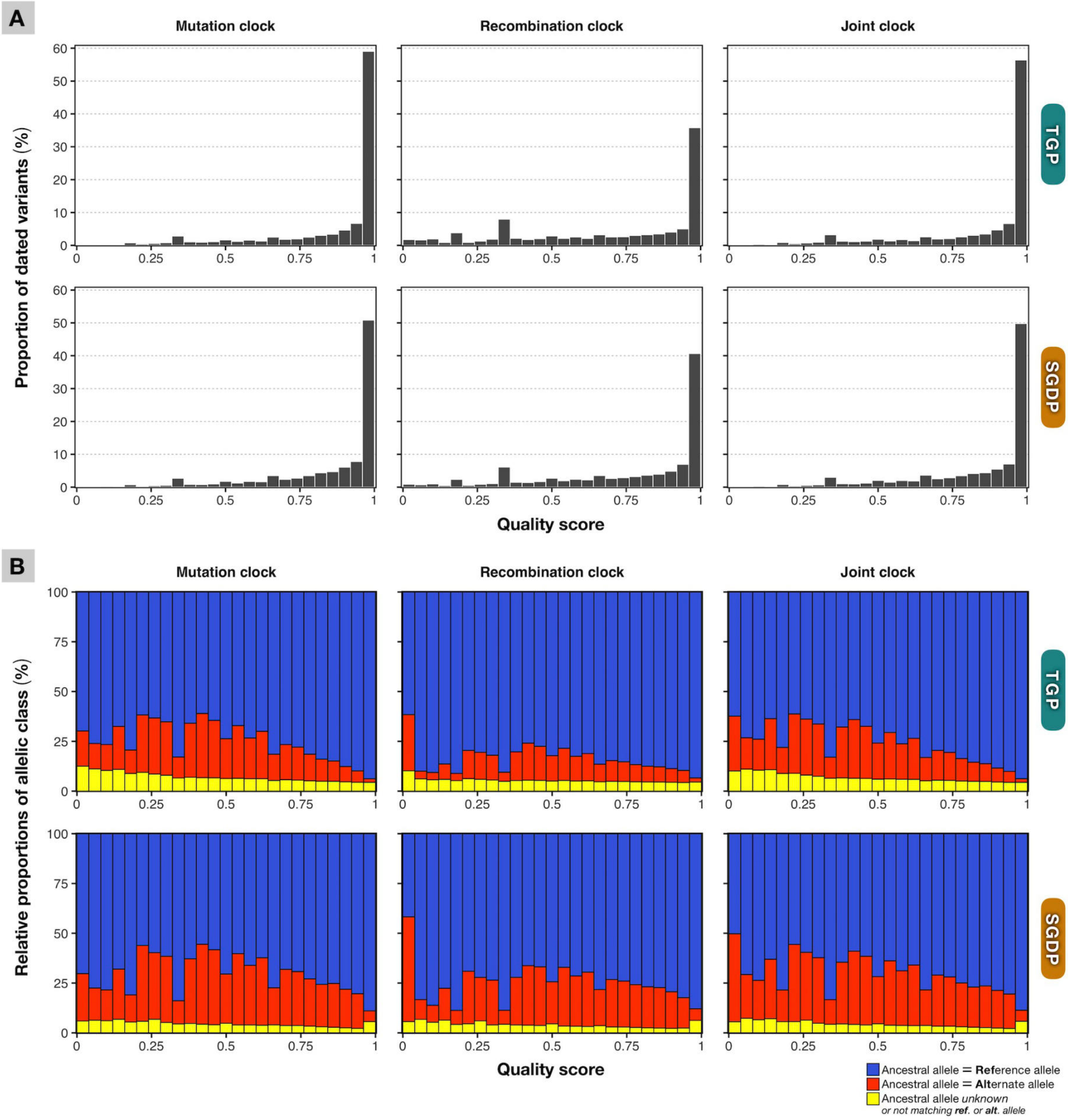

Quality score for variants dated in TGP and SGDP data. Panel **A** shows the histogram of the quality scores (QS) computed for variants dated in the 1000 Genomes Project (TGP; *top panels*) and the Simons Genome Diversity Project (SGDP; *bottom panels*), under each clock model. Histograms show the proportion of variants estimated with QC values within 25 equally distributed bins, relative to the number dated in each data source; 43,232,520 variants in TGP and 15,834,824 in SGDP. Corresponding to (A), Panel **B** shows the relative proportions of allelic class found in each QS bin; here defined as the ancestral allele matching the reference allele (*blue*), the ancestral allele matching the alternate allele (*red*), or where the ancestral allele was either unknown or did not match the reference or alternate alleles (*yellow*) at the variants (SNPs) dated in each data source. Note that GEVA attempts to estimate the age of the alternate allele by default, assuming that the reference allele represents the ancestral state.

3 Variants dated in Simons Genome Diversity Project (SGDP) data

Data available from SGDP consists of 276 individuals (556 genomes) from 130 populations worldwide (fully public data set; hg19/GRCh37). We used the already phased panel (labelled "PS2") that had been phased using SHAPEIT2.* Given the relatively small sample size of the SGDP panel (compared to TGP), we set $\max_{\mathcal{C}} = 100$ and $\max_{\mathcal{D}} = 100$ as the sampling limits for concordant and discordant pairs per variant. We estimated the age for 15.8 million variants across autosomes, which involved the analysis of 0.7 billion concordant and 1.5 billion discordant pairs. Overall computation time was 33,168 hours (3.8 CPU years; Intel Xeon E5-2650 v2, Ivy Bridge EP, 2.60GHz). Note that the processing time was on average faster compared to the analysis of TGP data, due to the smaller sample size and the lower sampling limits set per variant.

As with variants dated using TGP data, we found that the mode, mean, and median as point estimates of variant age were highly correlated (Pearson's $r^2 > 0.99$; Spearman's $\rho > 0.99$, based on all 15.8 million variants, in all comparisons between mode, mean, and median, under each clock model).

We additionally re-estimated the age of >0.36 million variants on Chromosome 20 using $\max_{\mathcal{C}} = 500$ and $\max_{\mathcal{D}} = 500$, to assess differences resulting from estimation with lower and higher sampling limits. We found that age estimates were highly consistent under each clock model (Spearman's $\rho > 0.9$), indicating that the lower sampling limits ($\max_{\mathcal{C}} = 100$, $\max_{\mathcal{D}} = 100$) were sufficiently high for the analysis of variants in SGDP; see figure below.

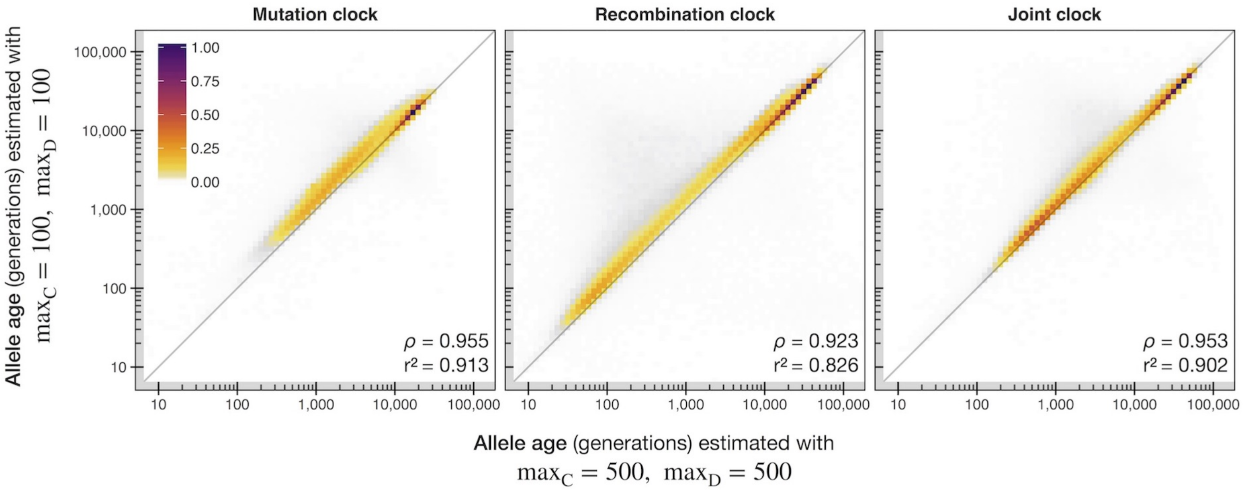

Consistency of variant age estimated with high and low sampling limits. Density scatterplot showing the relationship between allele age estimated with different sampling limits for concordant and discordant pairs; set to $\max_{\mathcal{C}} = 500$ and $\max_{\mathcal{D}} = 500$ (*x-axis*), and set to $\max_{\mathcal{C}} = 100$ and $\max_{\mathcal{D}} = 100$ (*y-axis*). Age was estimated for all variants on Chromosome 20 in SGDP (>0.36 million) under each clock model. Colors indicate the relative density scaled by the maximum per panel. The inserts (*bottom*) show the correlation between the two analyses; Spearman rank correlation coefficient (ρ) and the square of the Pearson correlation coefficient (r^2; calculated on log-scaled allele ages).

* https://sharehost.hms.harvard.edu/genetics/reich_lab/sgdp/phased_data/PS2_multisample_public/

Estimation quality, measured after rejecting outlier haplotype pairs per variant and under each clock model, is shown in panel A of the figure shown on Page 4. For example, median quality score (QS) was 0.960 for the joint clock model (0.969 mutation clock; 0.905 recombination clock), and the proportion of variants with $QS > 0.95$ was 51.8% (joint clock; 53.1% mutation clock; 42.8% recombination clock). Differences in quality with regards to the ancestral state of the reference or alternate allele are shown in panel B on Page 4. As in the analysis of variants in TGP, the majority of variants in SGDP (81.5%) had the ancestral allele as the reference allele, for which median QS was 0.980 (joint clock; 0.980 mutation clock; 0.929 recombination clock). However, compared to estimation in TGP data, we found that a higher proportion of variants had the ancestral allele as the alternate allele (14.0%), and differences in quality were not as pronounced as in TGP; median QS was 0.794 (joint clock; 0.816 mutation clock; 0.737 recombination clock). The relative proportion of variants with $QS > 0.95$ (joint clock) was 56.2%, 22.3%, and 64.1% for sites where the ancestral allele matched the reference, alternate, or neither allele, respectively. Differences in estimation quality measured for variants dated in TGP and SGDP may also arise from the different sampling limits applied. Note that we used external data to determine the ancestral allele (Ensembl EPO pipeline; Section 1), and that we included this information only at sites where genomic position and bases at both alleles matched unambiguously.

4 Combined age estimation of variants present in TGP and SGDP

The true age of a variant refers to the time of a singular mutation event in the past, which follows from the assumption (infinite sites model) that mutations occur only once per genomic locus in the history of the population. Although this assumption is readily violated in reality, in particular for very old mutations or if the mutation rate is high, we would nonetheless expect it to hold for the vast majority of variants observed in the (human) genome. Estimating the age of the same allele in different data sources, in which it has been identified in genomic sequence data of independent (unrelated) samples, should therefore yield consistent results.

We identified 13,673,639 variants present as biallelic SNPs (non-singletons and below 100% allele frequency) in both TGP and SGDP; matched by genomic position and reference and alternate alleles. The ages of alleles at corresponding sites, independently estimated from TGP and SGDP data, were highly consistent; see **S5 Fig**. We then, additionally, estimated the age of these variants by combining information from both sources, to implicitly increase the genealogical resolution, but without combining sequence data directly.

Recall that we estimate the age from the composite distribution of locally inferred TMRCA posteriors at concordant and discordant haplotype pairs; see **S1 Text**. The TMRCA posterior is modeled using the Gamma distribution, where parameters α, β are obtained from the data as defined by the clock model used (mutation clock, recombination clock, or joint clock). For each variant, we combined information by recovering TMRCA posteriors from the parameter values as obtained in the two data sources (without rejecting outliers). We estimated the

"combined" age, and computed a quality score, after rejecting outlier pairs in the combined sets of concordant and discordant comparisons.

Again, as with variants dated using TGP or SGDP data alone, we found that the mode, mean, and median as point estimates of variant age were highly correlated (Pearson's $r^2 > 0.99$; Spearman's $\rho > 0.99$, based on all 13.7 million variants, in all comparisons between mode, mean, and median, under each clock model).

References

1. Auton A, Abecasis GR, Altshuler DM, Durbin RM, Bentley DR, Chakravarti A, et al. A global reference for human genetic variation. Nature. 2015;526(7571):68–74.

2. Mallick S, Li H, Lipson M, Mathieson I, Gymrek M, Racimo F, et al. The Simons Genome Diversity Project: 300 genomes from 142 diverse populations. Nature. 2016;538(7624):201–206.

3. Takahata N. Allelic genealogy and human evolution. Molecular Biology and Evolution. 1993;10(1):2–22.

4. Tenesa A, Navarro P, Hayes BJ, Duffy DL, Clarke GM, Goddard ME, et al. Recent human effective population size estimated from linkage disequilibrium. Genome Research. 2007;17(4):520–526.

5. Park L. Effective population size of current human population. Genetics research. 2011;93(2):105–114.

6. Scally A, Durbin R. Revising the human mutation rate: implications for understanding human evolution. Nature Reviews Genetics. 2012;13(10):745–753.

7. International HapMap Consortium, et al. A second generation human haplotype map of over 3.1 million SNPs. Nature. 2007;449(7164):851.

8. McLaren W, Gil L, Hunt SE, Riat HS, Ritchie GRS, Thormann A, et al. The Ensembl Variant Effect Predictor. Genome biology. 2016;17(1):1–14.

9. Adzhubei IA, Schmidt S, Peshkin L, Ramensky VE, Gerasimova A, Bork P, et al. A method and server for predicting damaging missense mutations. Nature Publishing Group. 2010;7(4):248–249.

10. Kumar P, Henikoff S, Ng PC. Predicting the effects of coding non-synonymous variants on protein function using the SIFT algorithm. Nature Protocols. 2009;4(7):1073–1081.
