# Supplementary material for: Dating genomic variants and shared ancestry in population-scale sequencing data
Source: PLoS Biol. 2020 Jan 17;18(1):e3000586. doi: 10.1371/journal.pbio.3000586 (PMC6992231; doi:10.1371/journal.pbio.3000586)
Supplement: S4 Text — (PDF) [file pbio.3000586.s017.pdf]

S4 Text

Dating of genomic variants and shared ancestry in population-scale sequencing data

Patrick K. Albers[1*] and Gil McVean[1]

[1]Big Data Institute, Li Ka Shing Centre for Health Information and Discovery, University of Oxford, United Kingdom

[*]patrick.albers@bdi.ox.ac.uk

Contents

1 Cumulative coalescent function (CCF) 1
2 Coalescent intensity function (CIF) 5
3 Effective population size (N_e) equivalent 5
4 Aggregation of CCFs across chromosomes 6

Shared ancestry inference

The ancestry shared between two haploid genomes can be described by the cumulative coalescent function (CCF), which is a monotonically increasing "coalescent profile" that expresses the fraction of a given (*target*) genome that has coalesced with another (*comparator*) genome up to a given point back in time. We use a dynamic programming technique for maximum likelihood decoding of the CCF, which operates on the distribution of allele sharing observed between two haplotype sequences, given the (estimated) ages of alleles. The method assumes independence of variants and ignores error in age estimates. We implemented this method in `C++` and made the source code available online.[*] The rationale of the method is illustrated in the figure on Page 2; the following section describes the algorithm in detail.

1 Cumulative coalescent function (CCF)

Let M be the number of mutations carried by a given target genome for which the age, denoted by ϕ, has been estimated. These are sorted from most recent to oldest age to obtain a sequence of time points, indexed by $i = 0, 1, 2, \ldots, M-1$, such that

$$\phi_0 \leq \phi_1 \leq \phi_2 \leq \ldots \leq \phi_{M-1}.$$

Corresponding to the above, we generate a sequence of observations,

$$\omega_0,\ \omega_1,\ \omega_2,\ \ldots,\ \omega_{M-1}$$

[*] `https://github.com/pkalbers/ccf`

where each observation in the age-sorted sequence is encoded as

$$\omega = \begin{cases} 1 & \text{if } \textit{shared} \\ 0 & \text{if } \textit{unshared} \end{cases} \tag{1}$$

to indicate the two possible observation states; *shared* if both the target and the comparator genomes carry the mutation, or *unshared* if only the target genome carries the mutation. The shared state indicates that the two genomes coalesce more recently than (or at) the time of the mutation event, while the unshared state indicates coalescence further back in time. Note that we only consider mutations carried by the target genome, because the times of unshared mutations carried by only the comparator are independent from the timings of coalescent events along the target genome.

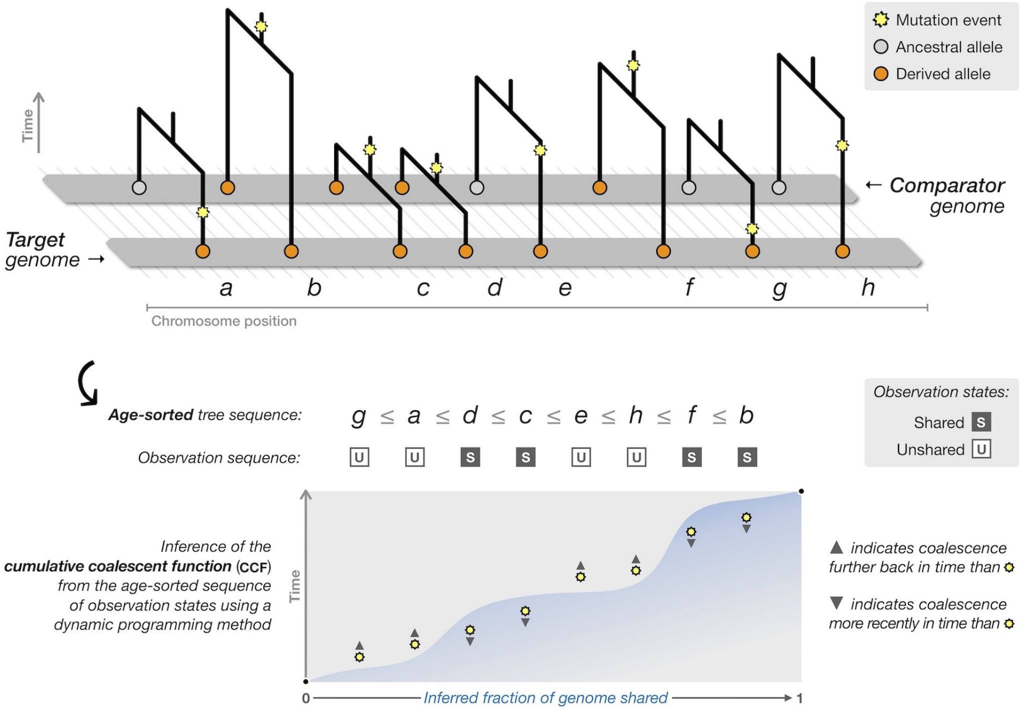

Schematic of shared ancestry inference using dynamic programming. The figure shows a schematic representation of two genomic sequences (*top*); labels $a, b, \ldots, h$ identify the derived alleles (*orange*) carried by a given target genome, for which allele age information are available. The genealogical relationship between the target and a given comparator genome is characterized by a sequence of trees. The mutation event that gave rise to an allele is indicated (*yellow*) on each tree at some point in the past. We define two observation states; *shared* (both the target and comparator carry the derived allele) and *unshared* (only the target carries the derived allele). Unshared alleles where only the comparator carries the derived allele are ignored. Given knowledge about the age of alleles, we sort the trees by the time of mutation (from youngest to oldest) to obtain the age-sorted observation sequence of shared and unshared states (*middle*). The time of coalescence between the two genomes is indicated by the shared or unshared state (*bottom*); we expect shared alleles to coalesce more recently than the time of mutation, and unshared alleles to coalesce further back in time, assuming that mutations and the trees on which they occurred are independent. Knowledge about the times of the mutations carried by a given target genome allows us to determine the relative order of events of lineages coalescing with the target genome. We thereby seek to infer the fraction the target genome shares with the comparator genome from the sequence of coalescent events as a function of time; referred to as the cumulative coalescent function (CCF), which we infer using a dynamic programming method. In the figure, the inferred CCF is indicated by the shaded area (*blue*).

The fraction of the ancestry a given target genome shares with another genome is discretized at the level of a single nucleotide. This being impractical for analytical purposes, we use a discrete choice approach as an approximation technique. We define $\{\delta_j\}_{j=0\ldots S}$ as the parameter space of possible states for the CCF, where $0 < \delta_j \leq 1$. For S states, we obtain

$$\delta_j = \frac{j}{S}, \text{ for } j = 1, 2, \ldots, S \tag{2}$$

and set $\delta_0 = \varepsilon$, where ε is reasonably small; $0 < \varepsilon \ll 1/S$.

Our goal is to infer the most likely sequence of fractional states that explain the age-sorted sequence of observed sharing states, such that we can subsequently assign an ancestry proportion (fraction) to the time of each mutation and, hence, infer the CCF as a function of the time by which the two genomes share a certain fraction of their genomes due to common ancestry.

The observation sequence is treated as a series of M independent Bernoulli trials, where the probability of observing the shared state is $P(\omega = 1) = p$, and $P(\omega = 0) = 1 - p$ for the unshared state, such that the probability mass function (PMF) is given by $f(\omega \mid p) = \omega p + (1 - \omega)(1 - p)$. The likelihood function for p as a parameter in a series of n observations is $L(p \mid \omega_1, \ldots, \omega_n) = \prod_{i=1}^{n} f(\omega_i \mid p)$. Importantly, we assume that p is proportional to the fraction of the ancestry shared between the two genomes; that is, the probability of observing the shared state is proportional to the CCF. This implicitly involves maximizing the likelihood $L(p_0, p_1, p_2, \ldots, p_{M-1} \mid \omega_0, \omega_1, \omega_2, \ldots, \omega_{M-1})$ for all possible combinations of the continuous probability parameters, which has no closed solution. We therefore define every $\{p_i\}_{i=0\ldots M-1}$ to take values in the fractional state space $\{\delta_j\}_{j=0\ldots S}$ and use dynamic programming to decode the most likely sequence of fractional states. The steps of the algorithm and inference of the CCF are formulated below. Note that the `C++` implementation of the algorithm performs certain operations on log-scale which, for simplicity, we have omitted here.

Initialization. Let $\mathbf{A}, \mathbf{B}$ denote two matrices of size $M \times (S + 1)$. These are initialized at the first site in the sequence, for states in $j = 0, 1, 2, \ldots, S$,

$$\mathbf{A}_{0,j} = \omega_0 \delta_j \ + \ (1 - \omega_0)(1 - \delta_j) \tag{3}$$

$$\mathbf{B}_{0,j} = 0. \tag{4}$$

In context of the recursion below, computing $\mathbf{A}_{i,j}$ becomes equivalent to obtaining the maximum likelihood over the discretized parameter space $\{\delta_j\}_{j=0\ldots S}$ conditional on the sequence of observations. Matrix $\mathbf{B}$ stores a pointer to the parameter that maximizes the likelihood at each position in the sequence.

Recursion. Moving along the sequence of observations, for $i = 0, 1, 2, \ldots, M-1$, we compute for each state in $j = 0, 1, 2, \ldots, S$,

$$\mathbf{A}_{i,j} = \big(\omega_i\delta_j \;+\; (1-\omega_i)(1-\delta_j)\big) \times \max_{k=0\ldots j}\big[\mathbf{A}_{i-1,k}\big] \tag{5}$$

$$\mathbf{B}_{i,j} = \operatorname*{argmax}_{k=0\ldots j}\big[\mathbf{A}_{i-1,k}\big]. \tag{6}$$

The recurrence relation in $\mathbf{A}_{i,j}$ is to update the likelihood incrementally, where $\mathbf{B}_{i,j}$ stores the state that makes $\mathbf{A}_{i,j}$ most likely. Note that k may not exceed the current index j, which is to ensure that transitions to the current state from a higher state have zero probability.

Termination. We define **Z** as a zero-indexed array of length M to store the index of states in the subsequent traceback step. Likewise, we define $\mathbf{P}$ as a zero-indexed array of length M, in which the inferred path sequence will be stored. At the last position in both arrays, we set

$$\mathbf{Z}(M-1) = S \tag{7}$$

$$\mathbf{P}(M-1) = \delta_S = 1 \tag{8}$$

which reflects the underlying assumption that the ancestry of the two genomes can be traced back, eventually, to a single origin in the past.

Traceback. We seek to find the optimal path through the parameter space $\{\delta_j\}_{j=0\ldots S}$ that maximizes the likelihood given the observation sequence. Tracing back from the terminal position, for $i = M-1,\ M-2,\ \ldots,\ 1$, we compute

$$\mathbf{Z}(i-1) = \mathbf{B}_{i,\mathbf{Z}(i)} \tag{9}$$

$$\mathbf{P}(i-1) = \delta_{\mathbf{Z}(i-1)}. \tag{10}$$

We then obtain the CCF by mapping the inferred state path at $\mathbf{P}_i$ to the corresponding variant age ϕ_i, which gives the inferred fraction of the genome shared at each time point in $\{\phi_i\}_{i=0\ldots M-1}$.

Finally, we define the CCF as a function of time, denoted by Λ_t for $t \in (0, \infty)$, where $\Lambda_0 = 0$ and $\Lambda_\infty = 1$. In practice, we approximate Λ_t using linear interpolation over a fixed grid of time points; for example, to jointly assess the CCFs from multiple individuals at the same time points in downstream analyses. For this, we write

$$\Lambda_t \;=\; \frac{\mathbf{P}_{k-1}\,(\phi_k - t) + \mathbf{P}_k\,(t - \phi_{k-1})}{\phi_k - \phi_{k-1}}\ , \quad \text{for}\ \ k = \operatorname*{argmin}_{i=1\ldots M}\big[\,|\phi_i - t|\,\big] \tag{11}$$

where t is taken from a fixed grid of L time points in $\{t_i\}_{i=1\ldots L}$, which may or may not overlap with the estimated allele ages in $\{\phi_i\}_{i=0\ldots M-1}$.

2 Coalescent intensity function (CIF)

For a given time interval (or "epoch"), we infer the intensity of coalescence between a target and comparator genome from the rate of change (gradient) of the CCF. Specifically, we compute the coalescent intensity function (CIF) as

$$\lambda(t_i, t_j) \ = \ \log\left[1 - \Lambda(t_i)\right] - \log\left[1 - \Lambda(t_j)\right] \tag{12}$$

where $t_i < t_j$, which denote the lower and upper time points that delimit a given epoch, respectively. The logarithmic difference approximates the percent change as a smooth function over time, which is appropriate given the continuous-time coalescent process. Note that the above is only defined for $\Lambda_t < 1$.

The CCF approximates the cumulative distribution of the time until the comparator genome has fully coalesced with the target genome, where the fraction shared between the two genomes is defined proportionally to time T as a random variable. Equivalently,

$$\Lambda_t \ \equiv \ F(T \leq t) \ \approx \ 1 - e^{-\int_0^t \lambda(u)\ du} \tag{13}$$

which follows from the assumption that coalescent events are mutually independent and the time to coalescence is approximately exponentially distributed with (instantaneous) rate $\lambda(t)$. Note that t is scaled in units of constant size N_0 (or, more commonly, denoted by N_e). The expression given in Equation (12) is therefore equivalent to the intensity parameter of a Poisson process; namely

$$\lambda(t_i, t_j) \ \equiv \ \int_{t_i}^{t_j} \lambda(u)\ du \ = \ \bar{\lambda} \times (t_j - t_i) \tag{14}$$

where $\bar{\lambda}$ is the average (constant) rate of coalescence during the epoch considered.

3 Effective population size (N_e) equivalent

Generally, changes in population size induce changes in the rate of coalescence, such that the inverse of the coalescent rate is equal to the relative population size at time t. Thus, the time-variable population size is given by the relation $N_t = N_0/\lambda_t$, where N_0 is the size constant by which time is scaled. Note that we have $\lambda_t = 1$ if population size is constant over time (equal to N_0).

Here, we use the CIF inferred for a given target genome to estimate the size of the ancestral population from which it derived in the past. Because the ancestry of a single genome is a distribution over many genealogical relationships, tracing back to different populations and at different points in time, we consider the pairwise coalescent history inferred between the target genome and a larger sample of comparator genomes, to obtain an estimate of the ancestral population size from the strongest signal of shared ancestry within a given

time interval. Analogous to notation of the effective population size in population genetics modeling, we refer to this estimate as the N_e *equivalent* (denoted by $N_{\tilde{e}}$ below).

For a given epoch that is delimited by time points t_i, t_j, we compute

$$N_{\tilde{e}}(t_i, t_j) \;=\; N_0 \times (t_j - t_i) \times \max_{k=1\dots n} \Big[\lambda_k(t_i, t_j) \Big]^{-1} \tag{15}$$

where k identifies the CIF in a sample of n comparators available for a given target. Note that we use the maximum CIF to obtain a non-parametric estimate of the population size, but which is restricted by the empirically determined parameter space $\{\lambda_k\}_{k=1\dots n}$ of the sample. Hence, in practice, CIFs are inferred against comparators from a large sample of individuals with both similar and diverse ancestral backgrounds, as $N_{\tilde{e}}$ tends to be overestimated if the intensity of coalescence with available comparators is low. Likewise, the length of an epoch is chosen to allow sufficiently many comparators to coalesce with a given target.

4 Aggregation of CCFs across chromosomes

The dynamic programing algorithm presented in Section 1 enables us to rapidly compute the CCF between every pair of haploid chromosomal sequences in large sample data sets. To generate a summary of the ancestry shared between diploid individuals (or groups of individuals), we combine information across the different chromosomes as follows. Consider a sample of N diploid individuals whose genome is constituted of K chromosomes (for example, $K = 22$ if considering autosomes in humans). We infer the CCF separately for each chromosome k, between each of the $2N$ target sequences in turn against $2N - 1$ comparator sequences. CCFs are approximated at a fixed grid of L time points to subsequently match chromosomes $1, 2, \dots, K$ at every time point in $\{t_i\}_{i=1\dots L}$. Profiles are aggregated, first, among diploid individuals and per chromosome k, by computing the average fraction Λ_t at each time point t, and then across chromosomes by computing the (weighted) mean, where weights might be assigned to different chromosomes, for example, conditional on the number of dated variants available per chromosome. This process is illustrated in the figure on Page 7, but also explained in more detail below.

There are four CCFs inferred between the two haploid sequences of a diploid target individual I and the two haploid sequences of comparator individual J, for $I \neq J$; namely

$$\Lambda_t^k(I_0, J_0),\; \Lambda_t^k(I_0, J_1),\; \Lambda_t^k(I_1, J_0),\; \Lambda_t^k(I_1, J_1)$$

where k refers to the chromosome currently considered, and the subscripts $0, 1$ indicate the haploid sequence per diploid individual. We also obtain two CCFs between the haploid sequences of the same individual ($I = J$); namely

$$\Lambda_t^k(I_0, I_1),\; \Lambda_t^k(I_1, I_0).$$

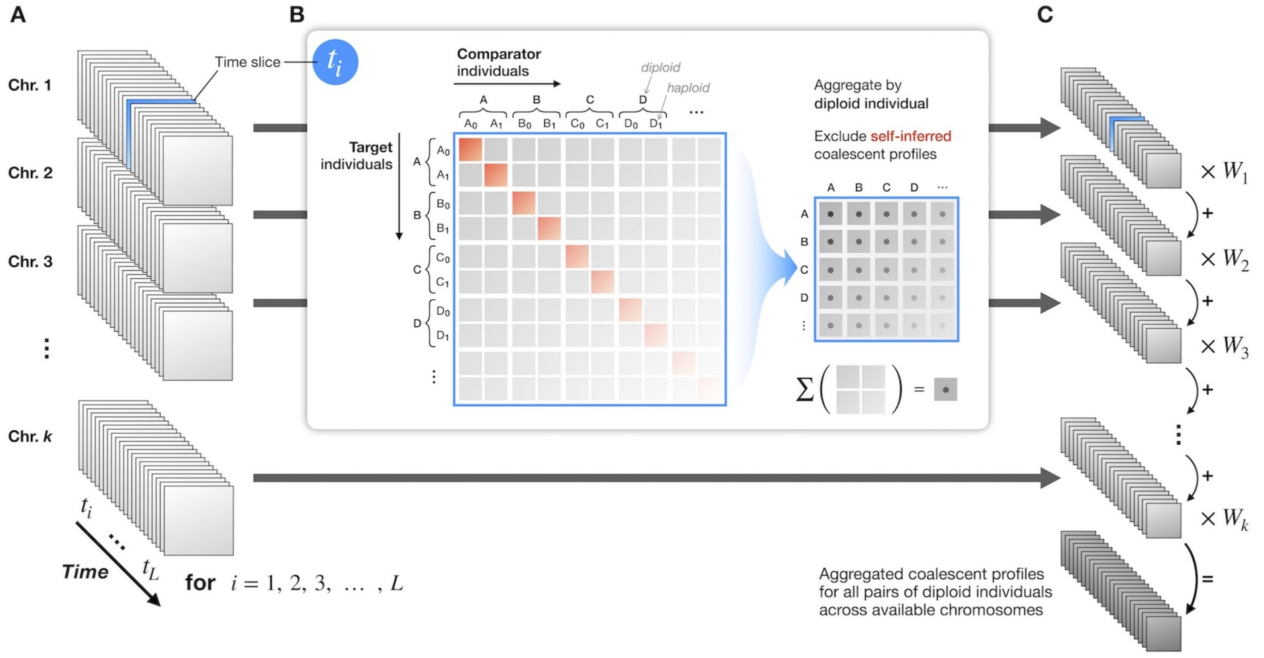

Aggregation of coalescent profiles per diploid individual. Panel **A** shows the schematic representation of the data available after inference of the CCF between each pair of haplotypes in a large sample of N diploid individuals. Each chromosome $1, 2, \dots, K$ can be represented as a three-dimensinoal matrix of size $2N \times 2N \times L$, where L is the number of time points to which CCFs have been approximated. Panel **B** illustrates the aggregation of CCF data per diploid individual. Every time "slice" in $\{t_i\}_{1\dots L}$ is represented as a $2N \times 2N$ matrix containing the inferred fraction (Λ_i) each of the $2N$ target haplotypes shares with each of the $2N - 1$ comparator haplotypes. We compute the average fraction among the four data points per pair of diploid individuals. Panel **C** shows the resulting, averaged matrices for each chromosome k, which are further aggregated into a single three-dimensional matrix of size $N \times N \times L$. This is done by computing the weighted mean across chromosomes, where weights ($\sum W_k = 1$) are assigned, for example, to up or down weight chromosomes with higher or lower numbers of dated variants, respectively.

The aggregated CCF between the target and the comparator individual is denoted by $\Lambda_t^*(I, J)$, which we compute as

$$\Lambda_t^*(I, J) = \sum_{k=1}^{K} \left(\frac{1}{4} \sum_{a=0}^{1} \sum_{b=0}^{1} \Lambda_t^k(I_a, J_b) \right) \times W_k \tag{16}$$

where W_k refers to the weight applied to each chromosome, for example, calculated as $W_k = v_k/u$, where v_k is the number of variants dated on chromosome k, and u is the sum of variants dated across all chromosomes considered. Similarly, to aggregate the CCFs within the same individual, we compute

$$\Lambda_t^*(I) = \sum_{k=1}^{K} \frac{1}{2} \left(\Lambda_t^k(I_0, I_1) + \Lambda_t^k(I_1, I_0) \right) \times W_k \,. \tag{17}$$

The above can be extended to aggregate the CCFs obtained for the haploid sequences of species with higher ploidy, or across groups of individuals to summarize the ancestry shared between defined demographic units.
