# Supplementary material for: Dating genomic variants and shared ancestry in population-scale sequencing data
Source: PLoS Biol. 2020 Jan 17;18(1):e3000586. doi: 10.1371/journal.pbio.3000586 (PMC6992231; doi:10.1371/journal.pbio.3000586)
Supplement: S5 Text — (PDF) [file pbio.3000586.s018.pdf]

S5 Text

Dating of genomic variants and shared ancestry in population-scale sequencing data

Patrick K. Albers[1*] and Gil McVean[1]

[1]Big Data Institute, Li Ka Shing Centre for Health Information and Discovery, University of Oxford, United Kingdom

[*]patrick.albers@bdi.ox.ac.uk

Contents

1 Generation of simulated data 1
2 Inference approach 1
3 Results and discussion 3

Inference of shared ancestry in simulated sample data

1 Generation of simulated data

We used `msprime` software [1] to simulate the demographic model illustrated in panel A of the figure on Page 2, which recapitulates the human expansion out of Africa [2]. Note that we also used this model for simulations in **S2 Text**, from which we now modified *Script 2* to simulate a sample of $N = 600$ haplotypes, consisting of an equal number of 200 haplotypes from each of the three simulated populations; African (AF), Asian (AS), and European (EU). Other simulation parameters were left unchanged. The model specifies three relevant events in the past; the time of the split of EU and AS from ancestral population B ($T_{EU-AS} = 848$ generations ago), the emergence of B from AF ($T_B = 5{,}600$ generation ago), and the emergence of AF from ancestral population A ($T_{AF} = 8{,}800$ generation ago). Only AS and EU experienced exponential population growth following T_{EU-AS}.

2 Inference approach

The simulated region encompassed 622,240 variant sites. We used GEVA to estimate the age of 412,348 variants (all SNPs with allele count $1 < x < N$) with $\max_{\mathcal{C}} = 100$ and $\max_{\mathcal{D}} = 100$ as the maximum number of concordant and discordant pairs sampled per site, and with scaling parameters as specified for the simulation (N_e =7,300; $\mu = 2.35 \times 10^{-8}$; variable recombination rates from HapMap Chromosome 20). Variant age estimated under the join clock model was used for the inference of shared ancestry. As for previous analyses of GEVA on simulated data, we first assessed the correlation between true and estimated allele age, which we found to be consistent with previous results; see panel B in figure on Page 2.

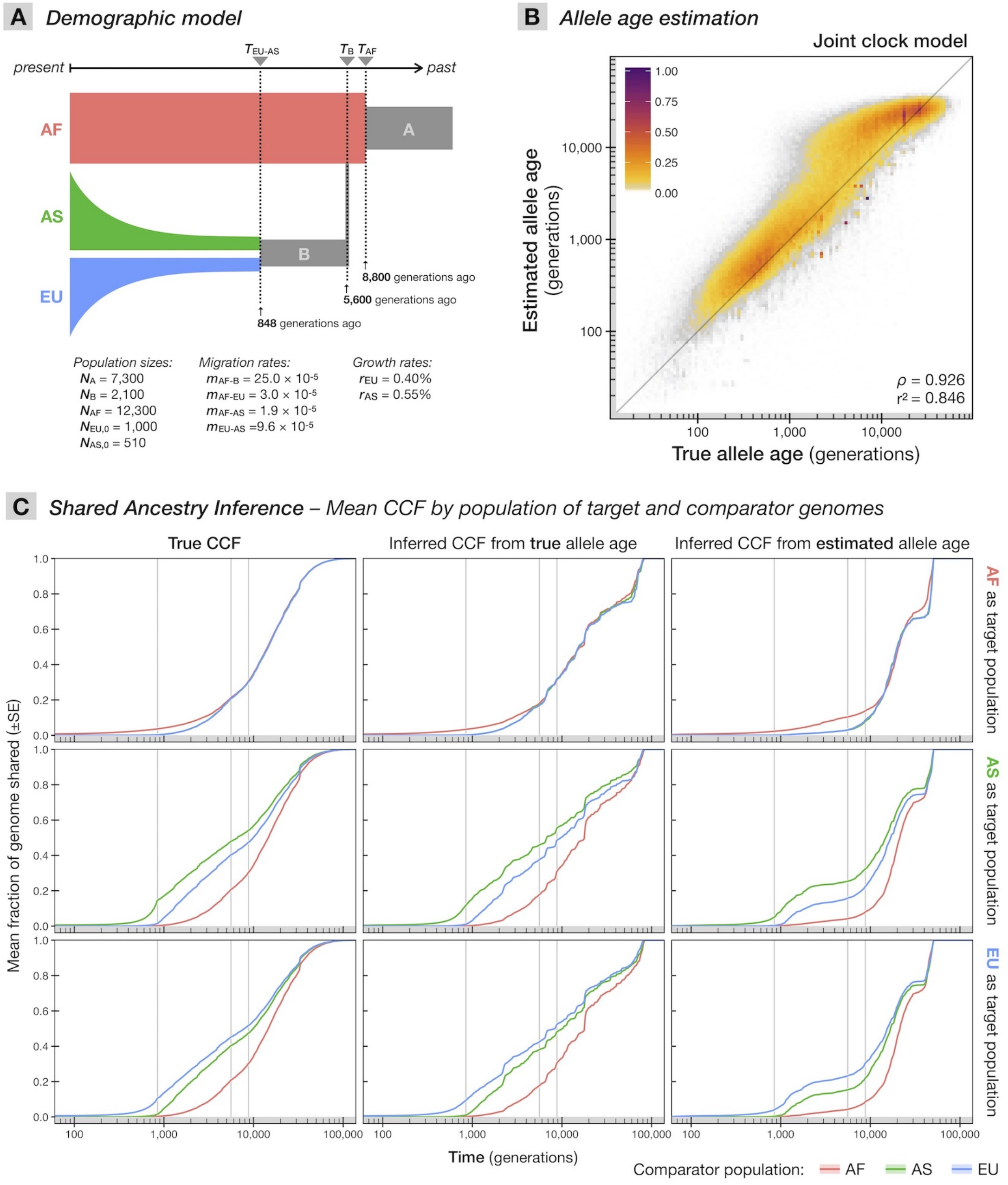

Results of shared ancestry inference in simulated data. (**A**) The demographic model used in coalescent simulations, which recapitulates the human expansion out of Africa [2], for three major populations; African (AF), Asian (AS), and European (EU). The times of three events are indicated (*top*); T_{AF} marks the time when AF emerged from ancestral population A, T_B the time when ancestral population B emerged from AF, and T_{EU-AS} the split of EU and AS from B. The time of each event assumes a generation time of 25 years per generation. The simulation was conducted with parameters as stated in the figure (*bottom*); that is, the size of each population, where $N_{AS,0}$ and $N_{EU,0}$ refer to the initial size of AS and EU, respectively, migration rates, and growth rates for AS and EU. We used this model to simulate a sample of $N = 600$ haplotypes (200 haplotypes from each of the three populations). (**B**) GEVA estimates of 412,344 variants (all variants at allele count $1 < x < N$), comparing true age (geometric mean of lower and upper age of the branch on which a mutation occurred; *x-axis*) and estimated age (joint clock model, $\max_{\mathcal{C}} = 100$, $\max_{\mathcal{D}} = 100$; *y-axis*). Colors indicate the maximized density; the insert shows the Spearman rank correlation statistic, ρ, and the square of the Pearson correlation coefficient (calculated on log-scaled age), r^2. (**C**) Shared ancestry inference, comparing coalescent profiles obtained from simulation records (true CCF; *left*), inferred from true allele age (*center*), and inferred from estimated allele age (*right*). We computed the CCFs for each of the 600 simulated haplotypes as target genome in turn against the whole sample as comparators; recorded over a fixed grid of 500 time points between 1 and 500,000 generations ago equally spaced on log-scale. Each line shows the mean and standard error (±SE) of CCFs between each combination of target and comparator population; for target genomes from AF (*top*), AS (*middle*), and EU (*bottom*). Vertical lines indicate the times of the three demographic events; T_{EU-AS} (*left*), T_B (*center*), and T_{AF} (*right*).

We inferred the ancestry of the simulated sample between every pair of haplotypes. This was done in three ways:

- **True CCF.** Coalescent profiles were obtained directly from simulation records, where we scanned the ancestry of a pair of lineages to determine the exact lengths of chromosomal segments that have coalesced up to a given point back in time. The fraction of the genome shared was computed as the sum of segment lengths divided by the full length of the simulated region, where we obtained the cumulative distribution at the exact time points of coalescent events.
- **Inferred CCF from true allele age.** The CCF between target and comparator genomes was inferred using the dynamic programming method described in **S4 Text**, but where we used the true age of alleles to obtain the age-sorted sequence of observed allele sharing. True age was determined from simulation records, which we computed as the geometric mean between the times of coalescent events that delimit the branch on which a given mutation occurred. We only considered those variants for which also the estimated age was available.
- **Inferred CCF from estimated allele age.** Coalescent profiles were inferred using the dynamic programming method with estimated allele ages (joint clock).

Inference of the CCF was done for every haplotype as target genome in turn against every haplotype in the sample (excluding itself) as comparator genome. True and inferred ancestry results were subsequently compared after approximating CCFs over a fixed grid of 500 time points between 1 and 500,000 generations ago (equally spaced on log-scale). Note that the dynamic programming method considers mutations carried by only the target genome. The inferred ancestry of target i shared with comparator j may therefore differ from the inferred ancestry of target j with comparator i. This is not the case for true CCFs, because these were generated having full knowledge of the sample ancestry, such that the ancestry measured between target and comparator i, j is identical to j, i.

3 Results and discussion

The CCFs obtained for the three strategies are compared in panel C of the figure on Page 2, which shows the average fraction of the genome shared back in time for each combination of target and comparator population. The times of the simulated demographic events are reflected by changes of the gradient along the ancestry shared within and between different populations.

In results of the true CCF, the ancestry shared among only AS genomes increases rapidly (exponentially) back in time until reaching T_{EU-AS}, and then increases constantly until T_B. The same is seen in the ancestry shared among only EU samples, but where the initial increase was less rapid (due to a lower growth rate). Sharing between AS and EU is low, and only increases further back than T_{EU-AS}. On average, the relationship of both groups to AF

genomes is indistinguishable, which is mirrored in the ancestry of AF genomes shared with genomes from either AS or EU. The ancestry shared among only AF genomes is higher (more recent) until reaching T_B, compared to the ancestry shared with either AS or EU, but then indistinguishable further back in time. Each comparison shows a gradient change at T_{AF}.

Ancestries inferred from true allele ages were overall consistent with patterns and times seen in the true ancestry profiles. For example, we see a rapid increase in the ancestry shared among only AS or only EU genomes until T_{EU-AS}, more recent shared ancestry among only AF genomes until T_B, and highly consistent gradients of the CCFs inferred between the different groups further back in time. However, we note that we infer artifacts at certain times, suggesting false changes in coalescent rates; for example at $\sim$2,500 generations ago among non-AF samples, but mostly in the distant past (>20,000 generations ago) and seen consistently across the whole sample. Such artifacts may result from incomplete information and variability of age-sorted sequences, as we only took a point estimate as the true age of mutations that may have occurred on relatively long branches.

For coalescent profiles inferred from estimated allele ages, we find notable differences between the values of the true and estimated fraction of the genome shared back in time. We also find artifacts that suggest false gradient changes, but again mostly limited to times in the distant past (>20,000 generations ago). The ancestry shared among genomes from the same population group increases rapidly in both AS and EU until $\sim$1,000 generations ago. Also, sharing between those groups is low until $\sim$1,000 generations ago, but which is slightly older than the actual time ($T_{EU-AS} = 848$ generations ago) of the split of AS and EU. Their split from AF is similarly shifted into the past, yet distinguishable from the ancestry of target genomes from AF shared with either AS or EU genomes. The ancestry of non-AF genomes shared with AF genomes is mirrored in the gradient and timing of the relationship between AF and non-AF genomes.

Overall, we note that the exact timings of events may not be reflected accurately by gradient changes along the inferred ancestry profiles, but we find the relative order of events to be consistent. Importantly, we find that ancestral relationships among and between different ancestry groups are qualitatively consistent.

References

1. Kelleher J, Etheridge AM, McVean G. Efficient Coalescent Simulation and Genealogical Analysis for Large Sample Sizes. PLoS Computational Biology. 2016;12(5):e1004842–22.

2. Gutenkunst RN, Hernandez RD, Williamson SH, Bustamante CD. Inferring the Joint Demographic History of Multiple Populations from Multidimensional SNP Frequency Data. PLoS Genetics. 2009;5(10):e1000695–11.
