# Supplementary material for: Dating genomic variants and shared ancestry in population-scale sequencing data
Source: PLoS Biol. 2020 Jan 17;18(1):e3000586. doi: 10.1371/journal.pbio.3000586 (PMC6992231; doi:10.1371/journal.pbio.3000586)
Supplement: S6 Text — (PDF) [file pbio.3000586.s019.pdf]

S6 Text

Dating of genomic variants and shared ancestry in population-scale sequencing data

Patrick K. Albers[1*] and Gil McVean[1]

[1]Big Data Institute, Li Ka Shing Centre for Health Information and Discovery, University of Oxford, United Kingdom

[*]patrick.albers@bdi.ox.ac.uk

Contents

1 Ancestry sharing in TGP. 2
2 Ancestry sharing in SGDP. 2

Inference of shared ancestry between individuals and population groups in publicly available data sets

We used the dynamic programming method presented in **S4 Text** to infer the cumulative coalescent function (CCF) between every pair of genomes within the 1000 Genomes Project (TGP) sample [1], as well as the publically available sample from the Simons Genetic Diversity Project (SGDP) [2]. Inference of the ancestry shared between individual genomes was conducted separately on data from Chromosomes 1-22. Information from the Atlas of Variant Age (as presented in **S3 Text**) was used to generate, in each analysis, the age-sorted sequence of observed allele sharing between the haploid chromosomes of a given target and comparator individual. Throughout, we used the mode as a point estimate of allele age under the joint clock model, for variants dated in TGP, as well as SGDP. Given the earlier results about the quality of dating as a function of variant covariates, we considered only those variants where the ancestral allele was known and matched the reference allele, and where estimation quality was reasonably high (quality score: $QS > 0.5$; see **S1 Text**). This is likely to introduce a small bias, but is at least partly compensated for by the the improved quality of the input data. The fraction of the genome shared was inferred at a resolution of $S = 200$ states (see **S4 Text**). We approximated the CCF over a fixed grid of 1,000 time points between 10 generations and 1 million generations ago (evenly distributed on log-scale). A summary profile was generated per diploid individual by aggregating individual CCFs as the weighted average across chromosomes, using the number of variants retained per chromosome to calculate weights (as described in **S4 Text**). The coalescent profiles inferred between each chromosomal pair in TGP and SGDP, as well as summaries of the ancestry shared with the whole sample, are available online:

https://human.genome.dating/ancestry/index

1 Ancestry sharing in TGP.

Of the 43.2 million variants dated in TGP, we retained 34.4 million across autosomes after filtering. We inferred the CCF between the haploid genomes from the 2,504 individuals available in the TGP sample, as well as the panel of 31 related individuals that were excluded from the final release data set.* Individuals in this additional panel comprised trios, parents, siblings, and second order relatives, who were part of the initial data set and also used to produce the variant call set of the final data set. Thus, we inferred the CCF for all 5,070 haploid chromosomes as target genomes, in turn against all others as comparator genomes, resulting in 25.7 million pairwise coalescent profiles per chromosome, and 6.3 million summary profiles after aggregating individual CCFs.

2 Ancestry sharing in SGDP.

Coalescent profiles were inferred on 11.7 million variants across autosomes, retained after filtering from the full set of 15.8 million variants dated in SGDP. We inferred the CCF between each pair of haploid chromosomes in the sample of 278 diploid individuals, resulting in 308,580 pairwise coalescent profiles per chromosome, and 77,284 summary profiles after aggregating individual CCFs. Additionally, we prepared cross-chromosome summaries for groups of individuals, by aggregating CCFs among individuals belonging to the 130 ancestry groups. This resulted in 16,900 summary profiles that describe the ancestry shared between each demographic unit.

References

1. Auton A, Abecasis GR, Altshuler DM, Durbin RM, Bentley DR, Chakravarti A, et al. A global reference for human genetic variation. Nature. 2015;526(7571):68–74.

2. Mallick S, Li H, Lipson M, Mathieson I, Gymrek M, Racimo F, et al. The Simons Genome Diversity Project: 300 genomes from 142 diverse populations. Nature. 2016;538(7624):201–206.

* ftp://ftp.1000genomes.ebi.ac.uk//vol1/ftp/release/20130502/supporting/related_samples_vcf
