# Supplementary material for: Dating genomic variants and shared ancestry in population-scale sequencing data
Source: PLoS Biol. 2020 Jan 17;18(1):e3000586. doi: 10.1371/journal.pbio.3000586 (PMC6992231; doi:10.1371/journal.pbio.3000586)
Supplement: Attachment — Submitted filename: GEVA_review (1).pdf [file pbio.3000586.s020.pdf]

# Review of "Dating genomic variants and shared ancestry in population-scale sequencing data"

September 2019

The manuscript presents a simple yet elegant method for estimating allele ages - genealogical estimation of variant age (GEVA). The method uses pairwise estimates of the TMRCA between haplotypes pairs. The TMRCA between concordant pairs that share the focal mutation should younger than the focal allele's age while the TMRCA of discordant pairs should be older than the focal allele's age. The authors therefore use pairwise TMRCAs to create a posterior distribution of the focal allele's age and use its mode as a point estimate for this age. The authors verify their approach using rigorous simulations. Allele age estimates are then used to estimate haplotype sharing between individuals and populations at different times in the past.
This manuscript presents exciting new results which will undoubtedly serve as a great resource for genomic research. GEVA provides a detailed view of human population structure, with incredible resolution in both space and time. In addition, GEVA's estimates of allele ages can help identify signatures of natural selections. GEVA will undoubtedly be used to analyze many different human data sets as well as many other organisms.
We do have, however, some questions and suggestions which might serve to improve the manuscript.

1. Two other recent methods also estimate allele ages. Speidel et al. (2019) estimated the whole local tree around common variants thereby also estimating allele age [1]. Platt et al. (2019) presented an estimator for allele age that also works well for extremely rare alleles [2].
   We think that a short paragraph about the similarities and differences

between the methods can strengthen the manuscript. It would make a reader aware of the other methods and provide information about when to prefer GEVA over them. If possible, adding some direct comparison of the methods would be really good.

2. GEVA uses the **mode** of the posterior distribution of allele ages as their point estimate of allele age. It is not immediately clear why would the mode be the most appropriate point estimate, as opposed to the mean or the median. Because many of the authors downstream analysis depends on these point estimates this choice is very important. Not only that, but one would suspect that most users of GEVA would only use the point estimates. It would therefore be very good if the authors examine the possible benefits of using a different point estimate.

3. How are confidence intervals for allele ages calculated? It seems like there are a lot of possible sources of uncertainty that may be included - uncertainty in point estimate, uncertainty in generation time, uncertainty in global mutation rate, variation in local mutation rates; etc. In addition, the pairs are correlated making it harder to get good CIs.

4. Both in simulations and inference on real data, it seems that very old alleles are all inferred to have the same age. This is most clearly evident in figures 5, S8 and S9, and is a phenomenon we encountered when trying to work with these estimates. Looking at figure S8, we can see that this is clearly an artifact. What is the source of this artifact? Can it be corrected? Could it be a result of using the mode of the posterior distribution as the point estimate?

5. GEVA's scalability for large data sets comes from sampling only a (small) subset of all possible pairs. However, doesn't this sampling also mean that method does not gain additional accuracy from increasing the sample size?

6. In Section S1.1 of the supplement, the authors claim
   "In principle, however, it is possible to also estimate the age of an allele if we only find one allele copy (singletons) or if all chromosomes are fixed for the derived allele, but which we have not considered here"
   Can the authors elaborate a bit here? With only discordant pairs for a singleton, wouldn't GEVA only give an upper bound on allele age? and

with only concordant pairs for a fixed derived allele, wouldn't GEVA only give an lower bound on allele age?

7. The authors claim that GEVA should only weakly depend on the prior on allele ages (equation 4 of the supplement). It would be nice to see this claim supported by simulations.

8. Though Section S2.1 of the supplement is much improved compared to the original biorxiv submission it is still **extremely** difficult to understand. It contains all of the technical details with none of the motivation. Figure S13, is a big step forward, but an explanation of the basic idea of recursively calculation the maximum likelihood is missing. We took the liberty of sketching such an explanation, that is we wrote down what would have helped us understand this section faster (see below).
   Also, with a large enough sample, wouldn't the calculation of the matrix A run into underflow problems? Isn't it better to work with log probabilities and replace the multiplication in equation (33) with a sum?

9. The emission probabilities of the HMM shared haplotype estimation were estimated using a specific demographic model. The same demographic model was also used in the simulations verifying the method. What's the sensitivity of GEVA for model misspecification here?
   (Also, did the authors try to rescale the mutation rate (and therefore time and population size) in the Gutenkunst model to u=1.2e-8?)

10. In Section S6.1, the mean of the posterior distribution is used as the point estimate of TMRCAs. Why use mean here and mode for point estimation of allele ages?

11. In Section S8, wouldn't restricting analysis to variants for which the ancestral allele is the reference allele create a bias?

12. In Figure 6, the population labels add little to the figure since it's very difficult to track any single population in the plots. Perhaps it's better to only label the broad ancestry groups (Africa, America etc.). This would also allow the label to be applied directly near the corresponding color in the 4 panels.

# References

[1] Leo Speidel, Marie Forest, Sinan Shi, and Simon Myers. A method for genome-wide genealogy estimation for thousands of samples. *bioRxiv*, 2019.

[2] Alexander Platt, Alyssa Pivirotto, Jared Knoblauch, and Jody Hey. An estimator of first coalescent time reveals selection on young variants and large heterogeneity in rare allele ages among human populations. *PLOS Genetics*, 15(8):1–25, 08 2019.

### Motivation for the DP in Section S2.1

The probability of observing $\{\omega\}_{i=0}^{M}$ is

$$P(\{\omega\}_{i=0}^{M-1}|\{\delta(\phi_i)\}_{i=0}^{M-1}) = \prod_i P(\omega_i|\delta(\phi_i))$$

with $P(\omega_i|\delta(\phi_i)) = \omega_i\delta(\phi_i) + (1-\omega_i)(1-\delta(\phi_i))$ and $0 \leq \delta(\phi_1) \leq \delta(\phi_2)\cdot \leq \delta(\phi_{M-1}) \leq 1$.
The maximum likelihood is therefore

$$ML(\{\delta(\phi_i)\}_{i=0}^{M-1}) = \max_{\{\delta(\phi_i)\}_{i=0}^{M-1}} P(\{\omega\}_{i=0}^{M-1}|\{\delta(\phi_i)\}_{i=0}^{M-1}).$$

We can define a set of functions with the following recursion relation

$$A_m(\delta) \equiv ML(\{\delta(\phi_i)\}_{i=0}^{m}|\delta_m = \delta) = \max_{\delta_m|0\leq\delta_m\leq\delta} P(\omega_m|\delta_m)A_{m-1}(\delta_m)$$

such that $ML(\{\delta\}_{i=0}^{M-1}) = A_{M-1}(1)$.
If we then discretize $\delta$, we can calculate $A$ recursively starting from $A_0(\delta) \equiv P(\omega_0|\delta_0)$ and building our way to $A_{M-1}(\delta)$. Once we have calculated $A_m(\delta)$ for all values of $m$ we can trace back through $A$ while maximizing the likelihood for each $\delta_i$.
