# Supplementary material for: Dating genomic variants and shared ancestry in population-scale sequencing data
Source: PLoS Biol. 2020 Jan 17;18(1):e3000586. doi: 10.1371/journal.pbio.3000586 (PMC6992231; doi:10.1371/journal.pbio.3000586)
Supplement: Attachment — Submitted filename: Albers_McVean_2019__response.pdf [file pbio.3000586.s021.pdf]

Dating genomic variants and shared ancestry in population-scale sequencing data

Patrick K. Albers and Gil McVean

We thank the reviewers for their helpful feedback and questions. In the following, we respond to each point raised in the reviewer's comments (given in full in the grey boxes). Note that we provide two files, for both the Manuscript and the Supplementary Information; one where changes to the previous version are highlighted in yellow, and another without highlights. Where applicable, we refer to revised or added sections of the Manuscript by line number.

Reviewer #1

> This paper describes a very useful and carefully built resource: an atlas of allele age estimates for the variants in a large database of publicly available genomes. The algorithm used to infer these allele ages is clearly described in the supplement and represents a cogent heuristic for tackling this difficult problem. GEVA has the potential to be a 1-stop shop for local ancestry inference and demographic inference, two important classes of methods that usually involve different assumptions and software.
>
> I have no significant concerns about the technical soundness of this manuscript. I do think it could be cleaned up a bit to improve its "user-friendliness"—the supplement is a very rich source of information, but it is not very well organized or indexed. I think the presentation of supplementary figures before the supplement index is counterproductive, and would prefer the index to be on the first page of this file so readers can more easily refer back to it.

Following the reviewer's advice regarding the organisational structure of our Supplementary Information, we moved the table of contents to the first page. The Supplementary Figures and Supplementary Tables are now also indexed in the table of contents. Additionally, we included brief summaries of each section on the first page of the Supplementary Text (page 13), to further improve readability and to highlight how the different sections are connected.

> The discussion of the paper is very short, especially given the breadth of results being presented, and it could be usefully expanded to flesh out how GEVA fits into the existing landscape of similar methods. For example, GEVA does some of the same things as tsinfer, another method from the McVean group, including inferring local ancestry profiles. Can the authors recommend when one local ancestry method may be more appropriate than the other, and how they are likely to compare to methods like chromopainter, RFmix, etc? In addition, the paper includes many PSMC-like plots, but it isn't clear whether the authors would recommend their method as an alternative to users who may be interested in running PSMC on new genomes from humans or other organisms. A biorxiv preprint by Leo Speidel, Simon Myers, et al. also estimates allele ages, and it would be useful to say something about the differences between the methods in performance and applicability.

We extended our introduction to include references to recently published methods for ancestry inference (lines 33–35), the 'tsinfer' (Kelleher et al. 2019) and 'relate' (Speidel et al. 2019) methods, highlighting that these methods mainly focus on the inference of genealogies, but where age can

be estimated indirectly from tree topologies. For example, 'tsinfer' is able to estimate relative ages from the order of nodes in local genealogies, and 'relate' places mutation events at the midpoint of branches to estimate age as the mean of the time between consecutive coalescent events. To highlight this difference, we now state that our method estimates age probabilistically, without reconstructing genealogies (lines 42–42). Note that Kelleher et al. (2019) cite an earlier version of our manuscript (bioRxiv: https://www.biorxiv.org/content/10.1101/416610v1) as a method to estimate the absolute age of mutations.

We introduce our approach to infer shared ancestry in context of existing methodologies (lines 210–217), and included references to the 'chromopainter' (Lawson et al. 2012) and 'RFmix' (Maples et al. 2013) methods (lines 218–223), to highlight the conceptual difference to our approach for inference of the cumulative coalescent function (CCF). Namely, the CCF captures ancestry proportions shared between individual genomes as a function of time, from which we can derive other parameters such as the intensity of coalescence and changes in relative relatedness over time, as well as changes in past population size (Ne equivalent); see Movie 1 and Figure 5C. However, we now mention that PSMC is expected to estimate Ne more accurately (lines 273–274). We also extended our discussion to provide a summary of our CCF methodology and potential applications (lines 356–367).

> The GEVA source code is available, but may or may not be as user-friendly as the database of variant ages that the authors obtained from a fixed set of publicly available genomes. Are the authors planning to keep updating the database as more genomes are sequenced, or are readers encouraged to run the method on other datasets themselves? Is it recommended for use on data from organisms other than humans? Do the authors expect that inferring demography directly from their coalescence time density functions might be more or less accurate than inferring demography using PSMC or other methods that incorporate site frequency spectrum information?

While we encourage researchers to use the GEVA source code, for example to estimate allele age in novel (unpublished) sequencing data sets or for other species, we also provide the Atlas of Variant Age as a ready-to-use source of information for the human genome. Since the Atlas represents a novel data resource, we hope that it will prove useful in future research, and that there will be increased interest in keeping the Atlas up-to-date, in particular as more data becomes available, and to further increase the accuracy of age estimates. We agree that it is important to update and extend the Atlas, and it is our intention to do so in the course of future studies or related applications. Similarly, we hope that there is interest in estimating the age of variants in data from species other than humans. This is now reflected in the discussion (lines 371–377).

Minor comments:

> A PLoS Genetics paper recently published by Alexander Platt, Jody Hey, et al. also estimates allele ages. This should probably be referenced.

We now included a reference to Platt et al. (2019) in our introduction (Ref #25).

> The supplement presents a nice empirically calibrated error model that is trained on the discrepancies between the Illumina Platinum Genomes and less accurate sequences generated from the same cell lines. The model appears to generate posterior probability estimates as to whether a given variant is an error or not. These error probabilities are likely to be useful in downstream applications of the variant allele age database. Are they searchable within the database? When a variant is queried, will it be flagged as probably an error if appropriate?

Our analysis of genotype error by comparison between data from IPG and TGP produced an empirical model of error rates conditional on allele frequency. We used these results to replicate realistic error distributions in simulated data, from which we then estimated the error rates conditional on both frequency and TMRCA (known from the record of simulated genealogies). We used TMRCA information to distinguish error rates for concordant and discordant pairs, to construct realistic models for the emission and initial state probabilities in the HMM. We now mention where to find these results in the "Availability of results" paragraph on page 15 of the manuscript, as well as the Supplementary Text (emission probabilities: Section S5.2, page 45; initial state probabilities: Section S5.3, page 48). However, note that this analysis may not directly translate into an application that could flag whether a particular allele has been called or typed with error.

Reviewer #2

> The manuscript presents a simple yet elegant method for estimating allele ages - genealogical estimation of variant age (GEVA). The method uses pairwise estimates of the TMRCA between haplotype pairs. The TMRCA between concordant pairs that share the focal mutation should younger than the focal allele's age while the TMRCA of discordant pairs should be older than the focal allele's age. The authors therefore use pairwise TMRCAs to create a posterior distribution of the focal allele's age and use its mode as a point estimate for this age. The authors verify their approach using rigorous simulations. Allele age estimates are then used to estimate haplotype sharing between individuals and populations at different times in the past.
> This manuscript presents exciting new results which will undoubtedly serve as a great resource for genomic research. GEVA provides a detailed view of human population structure, with incredible resolution in both space and time. In addition, GEVA's estimates of allele ages can help identify signatures of natural selection. GEVA will undoubtedly be used to analyze many different human datasets as well as many other organisms.
> We do have, however, some questions and suggestions which might serve to improve the manuscript.
> Two other recent methods also estimate allele ages. Speidel et al. (2019) estimated the whole local tree around common variants thereby also estimating allele age [1]. Platt et al. (2019) presented an estimator for allele age that also works well for extremely rare alleles [2].
> We think that a short paragraph about the similarities and differences between the methods can strengthen the manuscript. It would make a reader aware of the other methods and provide information about when to prefer GEVA over them. If possible, adding some direct comparison of the methods would be really good.

We now included a reference to the recently published paper by Speidel et al. (2019) in our introduction (lines 33–35). Briefly, their approach mainly focuses on the estimation of local tree topologies from the order of coalescent times, from which they can obtain estimates of allele age indirectly, by placing mutation events at the midpoint of branches (thus estimating age as the

mean of the time of consecutive coalescent events). This differs from our approach, because GEVA does not require to reconstruct the underlying genealogy (which we now mention on lines 41–42), but rather combines pairwise TMRCA information probabilistically.

> GEVA uses the mode of the posterior distribution of allele ages as their point estimate of allele age. It is not immediately clear why would the mode be the most appropriate point estimate, as opposed to the mean or the median. Because many of the authors downstream analysis depends on these point estimates this choice is very important. Not only that, but one would suspect that most users of GEVA would only use the point estimates. It would therefore be very good if the authors examine the possible benefits of using a different point estimate.
> How are confidence intervals for allele ages calculated? It seems like there are a lot of possible sources of uncertainty that may be included - uncertainty in point estimate, uncertainty in generation time, uncertainty in global mutation rate, variation in local mutation rates; etc. In addition, the pairs are correlated making it harder to get good CIs.

We agree with the reviewer's comment regarding the choice and availability of different estimators of allele age. In the Supplementary Text, we now included a paragraph explaining why we would expect that the mean and median converge on the mode of the composite posterior distribution (Section S1.4, page 24). This is further demonstrated through re-analysis of all variants in the Atlas of Variant Age, where we, in addition to the mode, also took the mean and median as point estimates. We compared the different estimators and report our results; for variants dated using TGP data (Section S7.2, page 57), SGDP data (Section S7.3, page 58), and for the combined set (Section S7.4, page 61). Briefly, we found that the correlation between estimators was very high (r2>0.99, rho>0.99) in all cases and for each clock model. Further, we computed confidence intervals for age estimates (also see Section S1.4, page 24) and make these new results available online, integrated in the Atlas of Variant Age.

> Both in simulations and inference on real data, it seems that very old alleles are all inferred to have the same age. This is most clearly evident in figures 5, S8 and S9, and is a phenomenon we encountered when trying to work with these estimates. Looking at figure S8, we can see that this is clearly an artifact. What is the source of this artifact? Can it be corrected? Could it be a result of using the mode of the posterior distribution as the point estimate?

We now expanded Section S1.4 in the Supplementary Text to describe the properties and practical limits of TMRCA inference and subsequent age estimation (pages 24–25). In particular, certain limitations arise from the loss of information over time, irrespective of the estimator. For example, the oldest alleles may be found to sit within shared haplotype segments that are only a few base-pairs long (with recombination breakpoints in between immediately neighbouring polymorphic sites). In such extremes, the variability in TMRCA (and to some extent age) is predominantly driven by the scaling parameters and the prior, as well as the mutation rate constant and variations of the recombination rate. However, we expect the prior to have less influence on more recent time scales.

Also, note that the web-application of the Atlas of Variant Age, for every variant, provides the option to "adjust scaling parameters", such as the value of the prior, to illustrate variations in time scales; for example, see https://human.genome.dating/snp/rs182549 (underneath the figure).

> GEVA's scalability for large data sets comes from sampling only a (small) subset of all possible pairs. However, doesn't this sampling also mean that method does not gain additional accuracy from increasing the sample size?

Our method scales well with larger sample sizes, in particular, because GEVA employs a sampling approach to only analyse a (small) subset of possible concordant and discordant pairs, where we sample concordant pairs at random and discordant pairs using a prioritisation algorithm. In the general case, we expect that this finds sufficiently many pairwise relationships that coalesce closely to the focal mutation event. Yet, we note that the accuracy of estimation can be further increased for particular variants, for example, if the underlying history cannot be traced back well enough within a given data set, or in data where the number of carrier haplotypes is low compared to global frequencies. As this depends on the composition of the sample and the genealogical resolution attainable from the data, accuracy could be improved by including more pairwise comparisons from additional (or larger) samples. We now extended our discussion to reflect this point, and emphasise that future applications of our method are important to update the Atlas of Variant Age (lines 371–377).

> In Section S1.1 of the supplement, the authors claim "In principle, however, it is possible to also estimate the age of an allele if we only find one allele copy (singletons) or if all chromosomes are fixed for the derived allele, but which we have not considered here" Can the authors elaborate a bit here? With only discordant pairs for a singleton, wouldn't GEVA only give an upper bound on allele age? and with only concordant pairs for a fixed derived allele, wouldn't GEVA only give an lower bound on allele age?

The time to the discordant/concordant pairs would indeed only give upper/lower bounds respectively. However, within the Bayesian framework, this information is sufficient to provide a posterior distribution for age in each case. As suggested, we now provide more detail regarding the use of only concordant pairs or only discordant pairs to obtain, in principle, an estimate for the upper or lower bound of allele age, respectively; see Section S1.1 (page 15) in the Supplementary Text.

> The authors claim that GEVA should only weakly depend on the prior on allele ages (equation 4 of the supplement). It would be nice to see this claim supported by simulations.

As alluded to in our response to a previous comment (regarding the source of putative artifacts in estimated allele ages), we now provide additional details regarding the dependence on (and limitations arising from) the prior in estimates obtained from the composite posterior distribution; see Section S1.4 (pages 24–25) in the Supplementary Text.

Though Section S2.1 of the supplement is much improved compared to the original biorxiv submission it is still extremely difficult to understand. It contains all of the technical details with none of the motivation. Figure S13, is a big step forward, but an explanation of the basic idea of recursively calculation the maximum likelihood is missing. We took the liberty of sketching such an explanation, that is we wrote down what would have helped us understand this section faster (see below).
Also, with a large enough sample, wouldn't the calculation of the matrix A run into underflow problems? Isn't it better to work with log probabilities and replace the multiplication in equation (33) with a sum?

Motivation for the DP in Section S2.1

The probability of observing $\{\omega\}_{i=0}^{M}$ is

$$P(\{\omega\}_{i=0}^{M-1}|\{\delta(\phi_i)\}_{i=0}^{M-1}) = \prod_i P(\omega_i|\delta(\phi_i))$$

with $P(\omega_i|\delta(\phi_i)) = \omega_i\delta(\phi_i) + (1-\omega_i)(1-\delta(\phi_i))$ and $0 \leq \delta(\phi_1) \leq \delta(\phi_2)\cdot \leq \delta(\phi_{M-1}) \leq 1$.
The maximum likelihood is therefore

$$ML(\{\delta(\phi_i)\}_{i=0}^{M-1}) = \max_{\{\delta(\phi_i)\}_{i=0}^{M-1}} P(\{\omega\}_{i=0}^{M-1}|\{\delta(\phi_i)\}_{i=0}^{M-1}).$$

We can define a set of functions with the following recursion relation

$$A_m(\delta) \equiv ML(\{\delta(\phi_i)\}_{i=0}^{m}|\delta_m = \delta) = \max_{\delta_m|0\leq\delta_m\leq\delta} P(\omega_m|\delta_m)A_{m-1}(\delta_m)$$

such that $ML(\{\delta\}_{i=0}^{M-1}) = A_{M-1}(1)$.
If we then discretize δ, we can calculate A recursively starting from $A_0(\delta) \equiv P(\omega_0|\delta_0)$ and building our way to $A_{M-1}(\delta)$. Once we have calculated $A_m(\delta)$ for all values of m we can trace back through A while maximizing the likelihood for each δ_i.

We thank the reviewers for providing us with a set of equations, which we have studied in detail. We agree that the motivation behind the dynamic programming approach may not directly follow from the technical description. We therefore made an effort to improve Section S2.1 of the Supplementary Text (pages 28–29). We included additional paragraphs that describe the goal of the method, the problems arising from working with both unknown and continuous parameters in terms of the likelihood, and our solution to discretise parameters and use dynamic programming to find the most likely path through the parameter space. Likewise, we attempted to improve the description of the steps of the algorithm. Also, regarding the potential to encounter underflow problems in computational applications, we now mention that our C++ implementation already performs certain steps of the algorithm on log-scale.

> The emission probabilities of the HMM shared haplotype estimation were estimated using a specific demographic model. The same demographic model was also used in the simulations verifying the method. What's the sensitivity of GEVA for model misspecification here? (Also, did the authors try to rescale the mutation rate (and therefore time and population size) in the Gutenkunst model to u=1.2e-8?)

We expect GEVA to be robust towards misspecification of the underlying simulation model. We have demonstrated this through analysis in the Supplementary Text; for fixed vs frequency-dependent transition probabilities (see results in Section S5.1, page 43, Figure S19B), and for different thresholds when constructing the emission model (see results in Section S5.2, page 46, Figure S21). Also, note that the definition of the emission model is time-independent, as we use a time threshold to distinguish emission rates for the concordant and discordant case. While variations in mutation rate are expected to shift emission probabilities, dependent on the threshold, our analysis shows that the model is robust when different thresholds are used, which also implies that the model would be robust under different mutation rates (within similar orders of magnitude).

> In Section S6.1, the mean of the posterior distribution is used as the point estimate of TMRCAs. Why use mean here and mode for point estimation of allele ages?

The composite posterior distribution (from which allele age is estimated) is bounded by the properties of the pairwise TMRCA posteriors (as we now explain in the Supplementary Text, Section S1.4, pages 24–25). However, these distributions are conceptually different. We use the Gamma distribution to infer the pairwise TMRCA posterior distribution, for which the mean has a simple closed form. Whereas the composite posterior is the result of combining TMRCA information across hundreds or thousands of Gamma distributions, using an approach similar to existing composite likelihood methods. Hence, the properties of the composite posterior, unlike the TMRCA posterior, cannot be predicted by a (simple closed) mathematical function. Likewise, the mean of the TMRCA posterior has no relation to the mean (or mode or median) of the composite posterior. Regardless, we now provide additional results for all variants in the Atlas of Variant Age, where we also report the mean and median (in addition to the mode) as point estimates of allele age.

> In Section S8, wouldn't restricting analysis to variants for which the ancestral allele is the reference allele create a bias?

We chose to apply this filter because our analysis of the quality of inference of age as a function of variant covariates identified this group as consistently having a higher quality than others. The effect of this filter will be to select against rare variants that the reference genome (which is of several ancestries) carries. This is a small fraction of all variant sites, so the impact of the bias is expected to be small, and, we felt, likely to be more than compensated for by the improved quality of the input data. We have not tested this assumption in great detail, but we now explain the filter and emphasise this point in in the Supplementary Text (Section S8, page 62).

> In Figure 6, the population labels add little to the figure since it's very difficult to track any single population in the plots. Perhaps it's better to only label the broad ancestry groups (Africa, America etc.). This would also allow the label to be applied directly near the corresponding color in the 4 panels.

We appreciate the reviewer's suggestion to reduce the complexity of Figure 6. However, we prefer to retain the labels for the different population groups; unless this is flagged as a major concern by the reviewer or the editors. We make several references to Figure 6 in the results presented in the manuscript (lines 277—304), where we mention several population groups by name. We would therefore argue that removing the labels would make it more difficult for readers to interpret the figure in the context of the results. Readers familiar with particular population groups may also want to inspect Figure 6 more closely to identify patterns of interest, which would be prohibited if the labels were removed.

Reviewer #3

> Just some quick comments from me. I like this paper, found it very interesting, and look forward to seeing it published. No major issues to raise, but I do have a couple of observations regarding the emphasis of the text which I think the authors should consider addressing.
>
> Firstly, it seems a bit odd that the first section of the paper, and indeed the first figure, places such emphasis on the comparison with using PSMC to estimate allele ages. This feels like inside baseball -- interesting from the perspective of algorithm development but not so important for understanding how it works. Perhaps I am missing something, but there didn't seem to be anything particularly surprising; using PSMC the method performed similarly, as one would expect, barring discretisation, in terms of accuracy if not speed, and the take-away message is that the authors' tailored approach was faster without adding bias or error. This is good but I think the simulation comparison alone without PSMC would be more meaningful for most readers, in terms of convincing the reader that the method does a good job at recovering allele age, which I think is the main task at this point in the paper. I'd put the PSMC aspect in the supplement.

Although we welcome the opportunity to reduce the length of the manuscript by moving the comparison of our method to PSMC into the supplements, we would like to express our preference for keeping it in the "Simulation study" section of the manuscript. That is, unless having this comparison in the main text is flagged as a major concern by the reviewer or the editors. We think that this comparison is relevant, because PSMC represents a principled (but computationally more demanding) approach for inference of demographic parameters. PSMC has been used in numerous studies and can be regarded as one of the integral methods in statistical and population genetics. The methodology of PSMC also forms the baseline on which several recently proposed methods have been developed. We therefore find it important to provide the results of this analysis in the main text. In particular, we think that by following the presentation of these results, readers are able to gain a better understanding of how our method works. To emphasise this point, we now mention why we think the comparison to PSMC is important (lines 70–71).

Also, to clarify a statement in the reviewer's comment, the first figure of the manuscript (Figure 1) only illustrates the methodology of GEVA, but contains no elements that refer (or compare our method) to PSMC. We assume that this comment alluded to Figure 2, which shows a direct comparison of results obtained through GEVA and PSMC, and which is the only figure in the manuscript that refers to (or shows results from) PSMC. We now state more clearly that Figure 1 only describes the approach within GEVA (line 43).

> Secondly, and perhaps more importantly, I'd like to see the paper give a bit more focus to the comparison with allele frequencies. A lot of people will approach this study and the resources presented as a more accurate way to incorporate allele age into their analyses than simply using allele frequency as a proxy. Figures 4A and S6 give some useful information, but I think the presentation and discussion is a bit limited. One thing to note, for example, is how contingent allele age is on population of ascertainment. For example the rarest alleles (<1%) if ascertained in Africa are on average about twice the age of equivalent alleles ascertained in Europe or East Asia. More pertinently, are there any cases where using frequency as a proxy might be misleading or biased, relative to using the ages estimated here? My overall impression is that frequency is a pretty good proxy for age if ascertained straightforwardly in a single G1k population - but the mapping is nonlinear and does not transfer between populations.

One aspect we intend to show is how contingent allele frequency is on population of ascertainment (as opposed to the age), because (unlike frequency) the age of an allele in principle is not expected to vary with the population or sample cohort in which its frequency is measured. We extended our discussion to better highlight the comparison between age and frequency (lines 345–355), and also mention that the examples provided (Figure 3) already suggest that frequency may not be a reliable proxy for the age. For example, Figure 3C shows an age estimate of around 5800 generations for a lower-frequency allele, whereas Figure 3A shows an age estimate of around 700 generations for a higher-frequency allele, and Figure 3B shows an age estimate of around 1500 generations, but where the allele is observed at both very high and low frequencies in different populations.

> There are also some interesting signals in figure S6. The text points out the striking signal of recent African admixture in America, but there is also an apparent sign of recent gene flow from Africa into Europe, manifesting as an excess of old but very rare alleles there. At least, that is how I read the plot in the fourth panel of the top row -- do the authors concur? A consequence of this is presumably also seen in figure 4A, where European variants older than ~25000 years are apparently more likely to be in the rarest frequency bin than the next most common.

This is an interesting observation that we had not picked up on, and we thank the reviewer for drawing it to our attention. We have now included the observation and the potential explanation in the main text (lines 189–192).
